# Mechanistic Insights Into Zn‐Promoted CO_2_ Electroreduction on α‐CuZn Alloys in Aprotic Media

**DOI:** 10.1002/cssc.70800

**Published:** 2026-10-30

**Authors:** Niklas Oppel, Liana Savintseva, Philipp Röse, Georg Kastlunger, Felix Studt, Ulrike Krewer

**Affiliations:** ^1^ Institute for Applied Materials – Electrochemical Technologies, Karlsruhe Institute of Technology Karlsruhe Germany; ^2^ Institute of Catalysis Research and Technology, Karlsruhe Institute of Technology Eggenstein‐Leopoldshafen Germany; ^3^ Department of Physics Technical University of Denmark Kongens Lyngby Denmark; ^4^ Institute for Chemical Technology and Polymer Chemistry, Karlsruhe Institute of Technology Karlsruhe Germany

**Keywords:** bimetallic catalyst, brass, cyclic voltammetry, density functional calculations, kinetic modeling

## Abstract

How Zn enhances the performance of Cu electrocatalysts for ${\mathrm{CO}}_{2}$RR in nonaqueous electrolytes remains an open question. Herein, we address this question by combining electrochemical measurements, kinetic modeling, and DFT calculations to elucidate Zn's role in boosting the electrocatalytic activity of Cu. Cyclic and linear sweep voltammetry reveal enhanced ${\mathrm{CO}}_{2}$ reduction activity with increasing Zn content, up to threefold higher current density at 25 wt.% Zn. Kinetic modeling attributes this to improved ${\mathrm{CO}}_{2}$ activation, while DFT confirms that Zn sites energetically stabilize Cu‐adsorbed ${\mathrm{CO}}_{2}$. This work uncovers the mechanism behind CuZn alloys’ superior performance, guiding design of efficient ${\mathrm{CO}}_{2}$RR catalysts for sustainable energy conversion.

## Graphical abstract

Elucidating nonaqueous CO_2_ reduction: Zinc boosts copper activity by tuning surface interactions for improved CO_2_ activation.

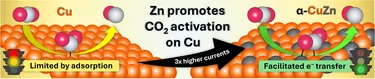

## 1 Introduction

Electrocatalytic ${\mathrm{CO}}_{2}$ reduction (${\mathrm{CO}}_{2}$R) offers significant potential for facilitating the transition to a carbon‐neutral economy by converting excess ${\mathrm{CO}}_{2}$ into valuable feedstock for the chemical industry [1]. The aqueous electrolytes typically employed for ${\mathrm{CO}}_{2}$R have multiple drawbacks, including limited ${\mathrm{CO}}_{2}$ transport to the electrode due to low ${\mathrm{CO}}_{2}$ solubility in aqueous solution [2, 3], catalyst instability leading to changes in product selectivity [4, 5], low carbon efficiency due to alkaline carbonate formation [6, 7], and the competing hydrogen evolution reaction (HER). The local reaction environment, including pH [8, 9, 10], cations [11, 12], and solvent [13, 14], has a tremendous impact on the activity of the ${\mathrm{CO}}_{2}$ reduction reaction (${\mathrm{CO}}_{2}$RR).

Aprotic organic electrolytes offer higher ${\mathrm{CO}}_{2}$ solubility and effectively eliminate the HER compared to aqueous electrolytes [15], yet they have received considerably less research attention [16]. The suppression of the HER allows for pure CO production without $H_{2}$ by‐products, thus distinguishing aprotic ${\mathrm{CO}}_{2}$R favorably from alternative ${\mathrm{CO}}_{2}$R techniques. For example, ${\mathrm{CO}}_{2}$R in solid oxide electrolysis cells typically comprises the HER because they cannot presently be operated without water [17]. The classical chemical ${\mathrm{CO}}_{2}$ to CO pathway via the reverse water gas shift reaction requires $H_{2}$ as the reducing agent [18]. Realizing pure CO production at a commercial scale via ${\mathrm{CO}}_{2}$R in aprotic organic electrolytes is thus highly attractive.

Due to the absence of protons, aprotic organic electrolytes enable distinct reaction pathways toward either CO with Faradaic efficiency (FE) beyond 95% [19] or oxalate with a FE of up to 80% [20]. One of the main drivers in deciding product selectivity is the choice of the aprotic solvent [21]. Among these, acetonitrile stands out due to its eightfold increased ${\mathrm{CO}}_{2}$ solubility compared to water, a wide electrochemical stability window, and its tendency to favor CO formation over oxalate as the main product [22].

In addition to the solvent, the salt of the supporting electrolyte significantly affects ${\mathrm{CO}}_{2}$R by modulating the interfacial environment. Although electric double layer and cation effects have been well studied in aqueous ${\mathrm{CO}}_{2}$R [23], their role in aprotic systems remains less explored. In aprotic solvents, typically quaternary alkylammonium salts with weakly coordinating anions are utilized, as alkali cations, such as Cs^+^, have been shown to passivate the electrode surface [24]. Recent work has revealed a size effect of these alkylammonium cations on ${\mathrm{CO}}_{2}$RR rates, attributed to changes in interfacial electric field strength: Smaller cations are closer to the electrode, thereby increasing the electric field strength and facilitating the kinetically limiting ${\mathrm{CO}}_{2}$ activation [25].

Beyond modifying the local reaction environment, the structure and composition of the catalyst material are of major importance for ${\mathrm{CO}}_{2}$R performance. Binary metal catalysts offer a promising approach for tuning catalytic properties by combining two elements with complementary behavior [26, 27]. Notably, the combination of earth‐abundant Cu and Zn presents a compelling choice that has already been used in several electrochemical ${\mathrm{CO}}_{2}$R studies over the last few years [28, 29, 30]. Additionally, the well‐established use of CuZn based‐catalysts in thermo‐catalytic ${\mathrm{CO}}_{2}$ hydrogenation to methanol underscores the potential of this catalytic system for ${\mathrm{CO}}_{2}$ conversion [31, 32].

In aqueous electrolytes, Cu is known for its ability to reduce ${\mathrm{CO}}_{2}$ to a broad spectrum of hydrocarbons and oxygenates with limited selectivity, while Zn primarily produces CO [33]. In combination with Cu, Zn has been reported to enhance C─C coupling rates through the transfer of adsorbed CO from Zn to neighboring Cu sites, resulting in higher CO coverages that are beneficial for C─C coupling [34, 35]. However, the precise role of Zn in CuZn catalysts remains under debate in aqueous ${\mathrm{CO}}_{2}$ electrocatalysis and in thermal catalysis [36], and is even less understood in aprotic ${\mathrm{CO}}_{2}$ electrolysis, for which only a single experimental study from our group has been reported to date [37].

Our previous model‐assisted study of the ${\mathrm{CO}}_{2}$RR over Cu in acetonitrile suggested that ${\mathrm{CO}}_{2}$ adsorption on the cathode is the primary kinetic limitation, as opposed to the transport limitations of ${\mathrm{CO}}_{2}$ observed in aqueous electrolytes [3, 38]. Although increasing surface roughness and elevated pressure have been shown to partially mitigate this limitation [38], the intrinsic kinetic inhibition of Cu persists. Recent experimental work on CuZn catalysts in aprotic acetonitrile has demonstrated that incorporating Zn into Cu‐based catalyst nanoparticles significantly enhances the catalysts ${\mathrm{CO}}_{2}$ reduction activity toward CO production [37]. This enhancement was observed exclusively for catalysts within the homogeneous α‐CuZn (α‐brass) alloy phase. In contrast, pure Zn and CuZn catalysts with a Zn atomic fraction of 0.5 or higher exhibited poor performance [37]. The authors proposed that Zn improves the binding of ${\mathrm{CO}}_{2}$ and other oxygen‐containing intermediates on the catalyst surface, thus overcoming the identified limitation of ${\mathrm{CO}}_{2}$ adsorption [37]. However, the underlying mechanisms responsible for this promotional effect are not yet fully understood. Nevertheless, their elucidation is essential for the rational design of improved ${\mathrm{CO}}_{2}$RR catalysts and, thus, for efficient and high‐performing cells for ${\mathrm{CO}}_{2}$R in organic electrolytes.

To address this knowledge gap and elucidate the role of Zn in CuZn alloys on the kinetic limitations of the ${\mathrm{CO}}_{2}$RR in nonaqueous electrolytes, we conduct an in‐depth multiscale kinetic investigation of the ${\mathrm{CO}}_{2}$RR on Cu and CuZn alloy catalysts. We combine cyclic voltammetry (CV) with dynamic kinetic modeling to enable the microkinetic analysis of reaction rates and surface coverages. This approach provides comprehensive insight into the thermodynamic and kinetic limitations, as well as the catalyst state during operation, revealing two distinct mechanistic scenarios. Finally, we employ density functional theory (DFT) calculations to probe the atomistic origins of ${\mathrm{CO}}_{2}$RR on CuZn alloys and to resolve these two mechanistic scenarios.

Dynamic kinetic modeling that reproduces experimental measurements using a set of species and charge balances is a powerful tool for unraveling complex electrocatalytic reactions [2]. It has proven effective in identifying kinetic effects, such as surface coverages [39], elucidating the interplay between ${\mathrm{CO}}_{2}$RR, HER, carbonation, and transport [3], and assessing the catalyst state [40]. This methodology, further extended by Geppert et al. to binary oxide catalysts [41], can aid in understanding the role of Zn in CuZn alloys, especially when combined with DFT calculations. In our previous study, we employed dynamic kinetic modeling to identify ${\mathrm{CO}}_{2}$ adsorption as the rate‐limiting step of the ${\mathrm{CO}}_{2}$RR on Cu in acetonitrile [38]. However, the experimentally observed performance improvements of binary catalysts, such as CuZn, have not yet been systematically analyzed or mechanistically understood.

Atomistic simulations, particularly those based on DFT, are crucial to gain mechanistic insights into electrochemical reactions at electrode surfaces, such as the ${\mathrm{CO}}_{2}$RR [42, 43]. The widely used computational hydrogen electrode approach excels in aqueous systems but fails to capture kinetics or apply to aprotic solvents due to its reliance on proton activity in water [44, 45, 46, 47]. To address this, we employ DFT calculations within the so‐called sawtooth potential framework as the simplest model of an electrochemical interface [48], followed by analysis using grand canonical DFT (GC‐DFT), implemented through a constant charge methodology with an implicit solvation model [49]. This approach was extended by the recently proposed computational ferrocene electrode (CFE) model [50, 51], which enables a direct connection between the electrochemical potential and the electrode potential on the experimental scale. Combined with dynamic kinetic modeling, our DFT calculations yield adsorption energetics for ${\mathrm{CO}}_{2}$ and CO, as well as ${\mathrm{CO}}_{2}$ activation onset potentials on Cu and CuZn surfaces. Crucially, we demonstrate for the first time that the CFE approach provides a physically meaningful reference for electrocatalytic processes in aprotic media, thereby resolving ambiguous kinetic scenarios and enabling a more accurate investigation of the ${\mathrm{CO}}_{2}$RR mechanism.

This work presents the first in‐depth model‐assisted analysis of the ${\mathrm{CO}}_{2}$RR kinetics in aprotic electrolyte on CuZn alloys in the α‐phase to reveal the intrinsic performance‐enhancing role of Zn in the binary alloy. First, we present the experimental performance of α‐CuZn electrocatalysts for ${\mathrm{CO}}_{2}$R in aprotic electrolyte, followed by an in‐depth analysis using our dynamic kinetic model to elucidate the influence of the Zn content on the rates and surface coverages of intermediates. DFT calculations complement the experimental and modeling results by exploring ${\mathrm{CO}}_{2}$ and CO adsorbate properties and electron transfer energetics on Cu and Zn sites of the alloys. This approach yields a holistic, quantitative understanding of Zn's role on the kinetic limitations during ${\mathrm{CO}}_{2}$RR.

## 2 Results and Discussion

This study investigates the ${\mathrm{CO}}_{2}$ reduction on α‐CuZn alloy electrocatalysts with 10%, 20% and 25% Zn mass fractions, denoted as CuZn10, CuZn20, and CuZn25, respectively. These correspond to approximate atomic compositions of Cu
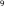
Zn, Cu
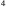
Zn, and Cu
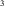
Zn. The electrochemical performance of pure Cu and Zn serves as benchmark. X‐ray diffraction patterns confirm a single‐phase, face‐centered cubic crystal structure for all α‐CuZn alloy samples (Figure S1). The structural stability of the α‐brass phase under these specific aprotic reaction conditions is confirmed by our recent operando X‐ray absorption spectroscopy study on CuZn nanoparticles [37], which showed no significant changes in the bulk oxidation state or local coordination during cathodic polarization. However, as this technique does not resolve the surface layer, reaction‐induced changes in surface roughness or near‐surface Zn redistribution of the planar electrodes used in this work cannot be entirely excluded.

### 2.1 Experimental Performance of α‐CuZn Electrocatalysts

The electrochemical behavior of planar Cu, Zn, and α‐CuZn alloys was investigated via CV and LSV in cathodic direction from −0.8 to −2.4 V in ${\mathrm{CO}}_{2}$‐saturated 0.1 mol L^−1^ TBABF_4_ in acetonitrile electrolyte at room temperature (Figure 1, see Experimental Section for details). All potentials in this work are referenced to Ag/AgCl (KCl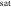 in $H_{2}O$).

**FIGURE 1:**
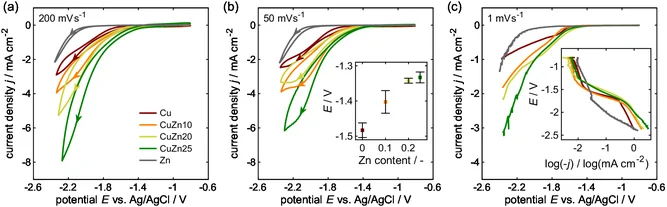
Electrochemical characterization of Cu, Zn, and α‐CuZn electrocatalysts in stagnant ${\mathrm{CO}}_{2}$‐saturated 0.1 mol L^−1^ TBABF_4_ acetonitrile electrolyte: (a) Cyclic voltammograms (CVs) obtained at 200 mV s^−1^, (b) CVs obtained at 50 mV s^−1^ with inset showing the onset potential as a function of Zn content, (c) LSV curves obtained at 1 mV s^−1^ with inset showing the corresponding Tafel plot. The CVs represent the averages of the third cycle from three independent measurements. For the full experimental dataset, see Figures S4–S9. Potentials are iR‐corrected and referenced against Ag/AgCl (KCl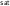 in $H_{2}O$). Arrows indicate sweep direction.

Figure 1a shows an increase in the maximum current density with an increase in Zn content for CVs at 200 mV s^−1^. In contrast, pure Zn exhibits the lowest current density and a delayed onset potential of −2.0 V compared to Cu and the CuZn alloys. A similar trend is observed at slower scan rates, e.g., 50 mV s^−1^ (Figure 1b), although the order of CuZn10 and CuZn20 is reversed. In the LSV measurements at 1 mV s^−1^ (Figure 1c), the trend of increasing current density with increasing Zn content in the CuZn alloys is preserved. Although pure Zn reaches a slightly higher current density at the most negative potential, it again shows a significantly delayed onset.

Contrary to previous reports on CuZn nanoparticle catalysts [37], we did not observe an optimum in ${\mathrm{CO}}_{2}$ reduction activity at CuZn20. Instead, CuZn25 consistently demonstrates the highest ${\mathrm{CO}}_{2}$R performance under all tested conditions.

The CVs for Cu and the α‐CuZn alloys exhibit a noticeable hysteresis that increases with a lower scan rate. As demonstrated in our previous work on Cu, this occurs because slower scan rates allow the system to spend more time at highly cathodic potentials, leading to a larger hysteresis width in the surface coverages of adsorbed intermediates [38]. In contrast, the CV of pure Zn shows minimal hysteresis.

The onset potential in the CVs, shown in the inset of Figure 1b, shifts to less negative values with increasing Zn content, indicating improved catalytic activity. This trend is also consistent with observations reported in the literature for Zn‐rich α‐CuZn nanoparticles [37]. The Tafel plots of Cu and α‐CuZn alloys (inset of Figure 1c) exhibit complex behaviors with several changes in slope as a function of potential. However, three regions of approximately linear behavior can be roughly identified: (i) an initial high‐slope region before the onset of electrochemical reactions, (ii) followed by a flatter intermediate region, and finally, (iii) another region of high slope at potentials below −1.7 V. This multi‐regime response violates the underlying assumptions of classical Tafel analysis, which presumes a single dominating rate‐determining step and constant coverage [52]. Consequently, a direct extraction of the charge transfer coefficient (α) from the experimental Tafel slopes is not feasible as the slopes do not represent a single elementary event [52, 53]. Therefore, dynamic microkinetic modeling is employed as a more robust technique to resolve changing surface coverages, identify potential‐dependent rate‐determining steps, and disentangle adsorption equilibria from transient rate profiles [2, 39, 40]. This approach allows for a determination of kinetic parameters, such as α, by accounting for the dynamic potential‐dependent behavior rather than relying on steady‐state treatments [3, 38].

For Cu and α‐CuZn alloys, the absolute values of the current densities decrease with decreasing scan rate in the CVs, indicating a transport or adsorption limitation. Investigating these limitations across the CuZn alloy series may reveal the specific contribution of Zn to enhanced catalytic performance. Therefore, in the following section, we apply a one‐dimensional dynamic kinetic model of the cathodic half‐cell to explore these limitations with the aim of identifying the origin of the enhanced performance of the CuZn alloys.

### 2.2 Kinetic Model‐Assisted Analysis

The physicochemical model and simulation framework employed in this study are based on our previous work [38] and are detailed in the Computational Methods section. The model describes the electrochemical reaction kinetics, the adsorption–desorption of ${\mathrm{CO}}_{2}$ and CO on the surface, and one‐dimensional diffusion of ${\mathrm{CO}}_{2}$, CO, and CO_3_^2−^ within the Nernst diffusion layer of a cathodic half‐cell with a planar electrode in stagnant, ${\mathrm{CO}}_{2}$‐saturated acetonitrile/TBABF_4_ electrolyte during a CV. Identifying limiting steps using steady‐state methods, such as polarization curves, is challenging because all rates are constrained to be equal. Therefore, we analyze these quantities under dynamic conditions to gain crucial insight into the prevailing limiting processes and to understand how Zn alloying influences them in the α‐CuZn alloys.

The assumed reaction mechanism involves reversible ${\mathrm{CO}}_{2}$ adsorption, one‐electron reduction to *CO_2_^−^ (where * denotes the adsorbed species), electrochemical disproportionation with nearby ${\mathrm{CO}}_{2}$ to form *CO and CO_3_^2−^, and CO desorption (Scheme 1). In line with the negligible ${\mathrm{CO}}_{2}$RR activity of pure Zn and the Cu‐like shape of the alloy CVs, the kinetic model treats only Cu‐type sites as explicitly active, while the effect of Zn is captured indirectly through the fitted kinetic parameters.

**SCHEME 1:**
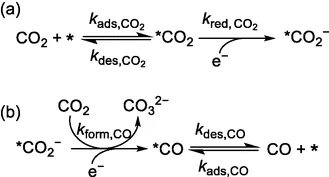
Reaction mechanism for ${\mathrm{CO}}_{2}$ reduction on Cu and α‐CuZn electrodes in dry aprotic electrolytes, as implemented in the kinetic model, and corresponding reaction and sorption rate constants $k_{i}$: (a) ${\mathrm{CO}}_{2}$ activation, and (b) CO formation and desorption.

Double‐layer capacitances were determined from CV scan‐rate dependence, electrolyte resistances were obtained from electrochemical impedance spectroscopy (EIS), and transport and solubility parameters were estimated using empirical correlations (see Table 1). The kinetic parameters were determined for each alloy composition separately by reproducing the experimental CVs at different scan rates. For pure Cu, two distinct sets of kinetic parameters were identified (Table 2), both of which reproduce the experimental CV data well for Cu, with similar normalized root mean squared errors of 0.0354 and 0.0350 (Table S7). The simulations with the corresponding parameters are labeled as Case A and Case B, respectively, and are shown in Figures 2a and 3a. As shown in Figure 4, both sets of parameters warrant further analysis as they exhibit significantly different parameters that lead to distinct conclusions about the underlying limitations.

**TABLE 1:** Model parameters used in the kinetic simulations of ${\mathrm{CO}}_{2}$ reduction on Cu‐based catalysts in 0.1 mol L^−1^ TBABF_4_ in acetonitrile.

Parameter	Symbol	Value	Unit
Geometric surface area of electrode	A	1.9635 × 10^−5^	$m^{2}$
Areal double‐layer capacitance of Cu electrode^a^	$C_{\text{dl,Cu}}$	0.124	$\mathrm{mF}\;{\mathrm{cm}}^{-2}$
Areal double‐layer capacitance of CuZn10 electrode^a^	$C_{\text{dl,CuZn10}}$	0.050	$\mathrm{mF}\;{\mathrm{cm}}^{-2}$
Areal double‐layer capacitance of CuZn20 electrode^a^	$C_{\text{dl,CuZn20}}$	0.031	$\mathrm{mF}\;{\mathrm{cm}}^{-2}$
Areal double‐layer capacitance of CuZn25 electrode^a^	$C_{\text{dl,CuZn25}}$	0.027	$\mathrm{mF}\;{\mathrm{cm}}^{-2}$
Uncompensated internal resistance^b^	$R_{u}$	86	Ω
Initial ${\mathrm{CO}}_{2}$ concentration (*p *= 1013.25 hPa, *T *= 298 K)^c^	$c_{{\mathrm{CO}}_{2},0}$	0.278	$\mathrm{mol}\;L^{-1}$
Henry solubility coefficient of ${\mathrm{CO}}_{2}$ in acetonitrile^c^	$H_{{\mathrm{CO}}_{2}}$	0.3113	$\mathrm{mol}\;L^{-1}\;{\mathrm{bar}}^{-1}$
Binary liquid diffusion coefficient of ${\mathrm{CO}}_{2}$ in acetonitrile^d^	$D_{{\mathrm{CO}}_{2}}$	4.617 × 10^−9^	$m^{2}\;s^{-1}$
Binary liquid diffusion coefficient of CO in acetonitrile^d^	$D_{\mathrm{CO}}$	5.043 × 10^−9^	$m^{2}\;s^{-1}$
Binary liquid diffusion coefficient of CO 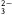 in acetonitrile^e^	$D_{{\mathrm{CO}}_{3}^{2-}}$	4.352 × 10^−9^	$m^{2}\;s^{-1}$

a From CV measurement (Figure S10).

b From EIS measurement (cf. section Electrochemical measurements).

c Calculated from Ref. [54].

d Calculated from Ref. [55].

e Estimated by linear extrapolation of the diffusion coefficients of ${\mathrm{CO}}_{2}$ and CO, based on their respective molecular weight.

**TABLE 2:** Kinetic parameters $k_{j}$, charge transfer coefficients $\alpha_{i}$, and areal concentration of adsorption sites Γ for Cases A and B identified from the analysis of CV data. A graphical overview of the identified parameters is shown in Figure S20.

		$\alpha_{\mathrm{red},{\mathrm{CO}}_{2}}$	$\alpha_{\text{form,CO}}$	Γ	$k_{\mathrm{ads},{\mathrm{CO}}_{2}}$	$k_{\mathrm{des},{\mathrm{CO}}_{2}}$	$k_{\mathrm{red},{\mathrm{CO}}_{2}}$	$k_{\text{form,CO}}$	$k_{\text{des,CO}}$	$k_{\text{ads,CO}}$
		–	–	$\mathrm{mol}\;m^{-2}$	$\mathrm{mol}\;s^{-1}\;m^{-2}$	$\mathrm{mol}\;s^{-1}\;m^{-2}$	$\mathrm{mol}\;s^{-1}\;m^{-2}$	$\mathrm{mol}\;s^{-1}\;m^{-2}$	$\mathrm{mol}\;s^{-1}\;m^{-2}$	$\mathrm{mol}\;s^{-1}\;m^{-2}$
Case A	Cu	0.8261	0.9144	1.834 × 10^−3^	5.424 × 10^−4^	4.314 × 10^−3^	4.208 × 10^−9^	1.229 × 10^−5^	2.300	1.027 × 10^−5^
	CuZn10	0.8261	0.8571	2.208 × 10^−3^	7.542 × 10^−4^	6.182 × 10^−3^	9.096 × 10^−9^	4.455 × 10^−8^	3.813	2.108 × 10^−6^
	CuZn20	0.8264	0.8609	2.017 × 10^−3^	1.220 × 10^−3^	9.980 × 10^−3^	1.837 × 10^−8^	3.382 × 10^−8^	3.696 × 0^−4^	6.757 × 10^−13^
	CuZn25	0.8264	0.8532	4.352 × 10^−3^	1.273 × 10^−3^	6.658 × 10^−3^	1.948 × 10^−8^	4.016 × 10^−8^	8.278	1.451 × 10^−6^
Case B	Cu	0.8470	0.7752	1.249 × 10^−4^	7.785 × 10^−2^	8.817 × 10^−3^	1.262 × 10^−9^	1.005 × 10^−10^	1.425 × 10^−4^	1.018 × 10^−9^
	CuZn10	0.8158	0.7971	1.646 × 10^−4^	9.924 × 10^−2^	9.475 × 10^−10^	1.251 × 10^−10^	1.458 × 10^−10^	2.081 × 10^−4^	3.219 × 10^−18^
	CuZn20	0.8158	0.6721	3.278 × 10^−4^	1.741 × 10^−2^	9.474 × 10^−3^	2.587 × 10^−10^	1.700 × 10^−10^	1.891 × 10^−4^	3.219 × 10^−7^
	CuZn25	0.8173	0.6096	4.293 × 10^−4^	9.531 × 10^−2^	5.328 × 10^−3^	2.587 × 10^−10^	7.170 × 10^−8^	3.495 × 10^−4^	3.459 × 10^−20^

**FIGURE 2:**
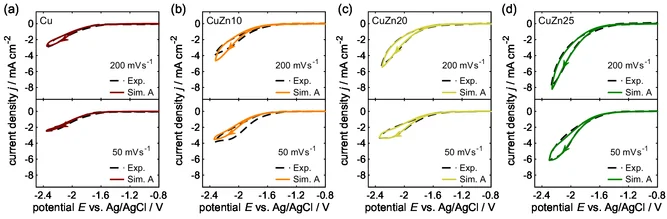
Experimental (dashed, black) and simulated (solid, colored) CVs for ${\mathrm{CO}}_{2}$ reduction on (a) Cu, (b) CuZn10, (c) CuZn20, and (d) CuZn25 electrocatalysts obtained at 200 and 50 mV s^−1^ in ${\mathrm{CO}}_{2}$‐saturated 0.1 mol L^−1^ TBABF_4_ in acetonitrile electrolyte at room temperature. Simulated curves are based on the kinetic parameter sets of Case A.

**FIGURE 3:**
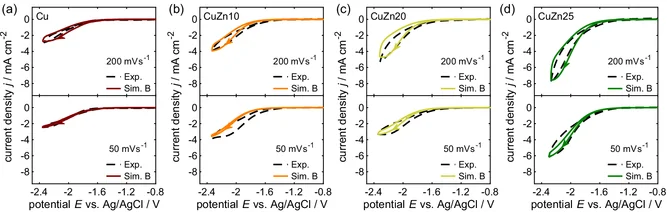
Experimental (dashed, black) and simulated (solid, colored) CVs for ${\mathrm{CO}}_{2}$ reduction on (a) Cu, (b) CuZn10, (c) CuZn20, and (d) CuZn25 electrocatalysts obtained at 200 and 50 mV s^−1^ in ${\mathrm{CO}}_{2}$‐saturated 0.1 mol L^−1^ TBABF_4_ in acetonitrile electrolyte at room temperature. Simulated curves are based on kinetic parameter sets of Case B.

**FIGURE 4:**
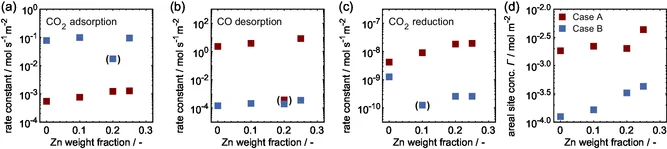
Selection of the identified parameter values for Case A (red) and B (blue) as a function of Zn weight fraction. Cases A and B refer to distinct kinetic parameter sets obtained from model optimization against CV data for CuZn alloys, representing alternative mechanistic explanations for enhanced ${\mathrm{CO}}_{2}$ reduction activity with Zn alloying. (a) ${\mathrm{CO}}_{2}$ adsorption rate constant $k_{\mathrm{red},{\mathrm{CO}}_{2}}$, (b) CO formation rate constant $k_{\mathrm{form},\mathrm{CO}}$, (c) CO desorption rate constant $k_{\mathrm{des},\mathrm{CO}}$ and (d) areal concentration of adsorption sites Γ. The complete parameter sets are shown in Table 2 and Figure S20. Outliers are marked by brackets ( ).

Case A aligns with our earlier work on Cu and features comparatively slow ${\mathrm{CO}}_{2}$ adsorption but fast CO formation and desorption. Its CV simulations predict that ${\mathrm{CO}}_{2}$ adsorption and the first electron transfer are rate limiting, while CO coverage remains negligible (Figure 5). Therefore, activity at highly cathodic potentials is mainly constrained by ${\mathrm{CO}}_{2}$ activation.

**FIGURE 5:**
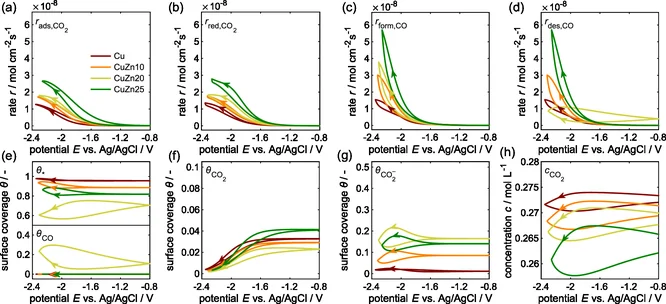
Simulated reaction rates and surface coverages during CV at 200 mV s^−1^ for Cu and α‐CuZn electrocatalysts based on Case A: (a) ${\mathrm{CO}}_{2}$ adsorption rate, (b) ${\mathrm{CO}}_{2}$ reduction rate, (c) CO formation rate, (d) CO desorption rate, (e) free surface site coverage (*) (upper part) and adsorbed CO coverage (*CO) (lower part), (f) adsorbed ${\mathrm{CO}}_{2}$ coverage (*${\mathrm{CO}}_{2}$), (g) adsorbed ${\mathrm{CO}}_{2}$ radical anion coverage (*CO_2_^−^), and (h) concentration of dissolved ${\mathrm{CO}}_{2}$ at the electrode–electrolyte interface.

In contrast, Case B exhibits a ${\mathrm{CO}}_{2}$ adsorption rate constant that is about a hundred times higher, but a substantially smaller CO desorption rate constant, i.e., $k_{\mathrm{ads},{\mathrm{CO}}_{2}}$ is about two orders of magnitude larger, while $k_{\mathrm{des},\mathrm{CO}}$ is roughly 1 × 10^4^ times smaller than in Case A (Figure 4). With these parameters, the model predicts extensive CO accumulation (>80% coverage) and a strong decline in available free sites at cathodic potentials (Figure 6). Thus, product removal, i.e., CO desorption, becomes the dominant limitation, effectively imposing CO poisoning on the Cu surface in Case B.

**FIGURE 6:**
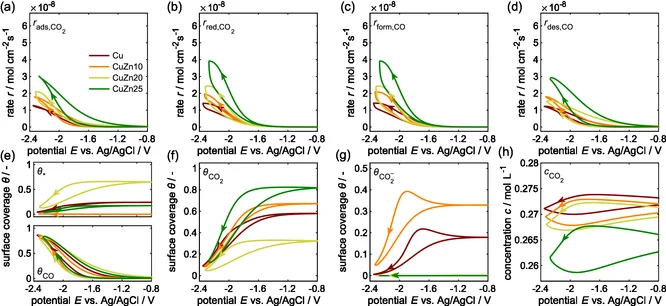
Simulated reaction rates and surface coverages during CV at 200 mV s^−1^ for Cu and α‐CuZn electrocatalysts based on Case B: (a) ${\mathrm{CO}}_{2}$ adsorption rate, (b) ${\mathrm{CO}}_{2}$ reduction rate, (c) CO formation rate, (d) CO desorption rate, (e) free surface site coverage (*) (upper part) and adsorbed CO coverage (*CO) (lower part), (f) adsorbed ${\mathrm{CO}}_{2}$ coverage (*${\mathrm{CO}}_{2}$), (g) adsorbed ${\mathrm{CO}}_{2}$ radical anion coverage (*CO_2_^−^), and (h) concentration of dissolved ${\mathrm{CO}}_{2}$ at the electrode–electrolyte interface.

Since CuZn catalysts outperform pure Cu, the alloys must at least partially overcome these identified limitations. The next step is, therefore, to examine whether the Cu parameter sets can be transferred to the CuZn alloys with only modest adjustments, and which parameters, and thus kinetic process steps, are primarily responsible for the kinetic change. If reproducing the data requires larger changes in the parameters, this Case is regarded less mechanistically likely. To test whether the changes in performance with Zn content could be captured by improving a single elementary step, individual kinetic parameters were varied, and the resulting CVs were compared to those of the CuZn alloys (Figures S22 and S23). The analyzed behavior comprises simultaneous increases in current density, shift of the onset potential, and nearly unchanged hysteresis with increasing Zn content. As no single parameter reproduces the experimentally observed effect of Zn content, this indicates the need for multiparameter optimization. Consequently, for each Case (A and B), separate multiparameter optimizations were carried out to obtain composition‐specific kinetic parameter sets for every CuZn alloy (Table 2).

Exemplarily shown for 50 and 200 mV s^−1^ in Figure 2, the model underlying Case A reproduces the experimental data across all compositions and scan rates, including the observed trends of increasing current density with increasing Zn content and increasing scan rate, as well as changes in the characteristic hysteresis. The same holds for additional scan rates in Figures S16–S19. The largest deviations between simulated and experimental data are observed for CuZn10. This is linked to the larger experimental variability observed for both CuZn10 and CuZn25 in three independent measurements (Figures S5 and S7). These two compositions exhibited significantly higher and nearly identical mean relative standard deviations of 0.155 and 0.144, respectively, compared to Cu (0.060) and CuZn20 (0.080). The precise origin of this variance remains unclear but is likely multifactorial, potentially stemming from sensitivities during mechanical polishing combined with distinct initial crystallographic orientations (Figure S1). Notably, while CuZn25 exhibits a similarly large experimental spread to CuZn10, it is reproduced much more accurately by the model, yielding the lowest normalized root mean squared error (Table S7). This apparent discrepancy is resolved by examining the nature of the experimental scatter: The CuZn25 replicates differ mostly in overall magnitude, preserving the fundamental CV shape, which the kinetic model captures effectively. In contrast, the variance for CuZn10 includes small but critical variations in the CV shape and characteristic hysteresis. Because the optimization objective function is highly sensitive to transient shape features, this structural variance results in the larger model deviations observed for CuZn10.

Figure 3 reveals for 50 and 200 mV s^−1^ that the model underlying Case B also captures qualitative trends but fails to reproduce the magnitude of hysteresis consistently (for further scan rates, see Figures S16–S19). For CuZn10 and CuZn25 at low scan rates, hysteresis is underestimated; for CuZn20 at high scan rates, it is overestimated. Further optimization of Case B's parameters did not yield significant improvements, suggesting that it may represent a local minimum that does not fully reflect the complex interplay of factors influencing the electrocatalytic activity of α‐CuZn alloys.

The identified parameters for Cases A and B exhibit mostly smooth trends with increasing Zn content within each Case, with only minor outliers (Table 2). However, the absolute parameter values differ markedly between Case A and Case B, with some dependencies on Zn content aligning while others oppose each other, yielding contrasting interpretations of Zn's promotional role in ${\mathrm{CO}}_{2}$R. Outliers are attributed to inherent uncertainties in parameter estimation—a common challenge in kinetic modeling arising from parameter identifiability issues, model simplifications, and experimental variability [56, 57]. This was particularly evident for the CO desorption and adsorption rate constants of CuZn20 in Case A (Figures 4 and S20). Adjusting these parameters to follow a linear trend with the other alloys (as shown in Figures S24 and S25) yielded slightly poorer CV fits but restored consistency with the behavior across the series. Thus, the analysis emphasizes overall trends across the alloy series rather than individual compositions.

In Case A, the rate constant for ${\mathrm{CO}}_{2}$ adsorption ($k_{\mathrm{ads},{\mathrm{CO}}_{2}}$) increases slightly with Zn content; however, due to a similar trend in the ${\mathrm{CO}}_{2}$ desorption rate constant, the equilibrium constant $K_{\mathrm{ads},{\mathrm{CO}}_{2}}$ does not change with increasing Zn content (Figure S20). Thus, Case A suggests a negligible influence of the Zn content on the net ${\mathrm{CO}}_{2}$ adsorption rate. However, the ${\mathrm{CO}}_{2}$ reduction rate constant ($k_{\mathrm{red},{\mathrm{CO}}_{2}}$) increases with increasing Zn content as well (Figure 4c), suggesting that improved ${\mathrm{CO}}_{2}$ activation plays a significant role in Case A.

In contrast, in Case B, the rate constant for ${\mathrm{CO}}_{2}$ reduction ($k_{\mathrm{red},{\mathrm{CO}}_{2}}$) decreases with Zn content, while the rate constants for CO formation ($k_{\mathrm{form},\mathrm{CO}}$) and CO desorption ($k_{\mathrm{des},\mathrm{CO}}$), including the equilibrium constant (Figure S20), show a slight increase. In this Case, therefore, Zn would improve performance via faster CO formation and desorption. This difference is also reflected in the sensitivity plots: Unlike Case A, where the simulated CV current is most sensitive to changes in the ${\mathrm{CO}}_{2}$ adsorption rate constant (Figure S22), the model output for Case B is most sensitive to an increase in the CO desorption rate constant (Figure S23).

Furthermore, both Cases predict a higher areal concentration of adsorption sites (Γ) for the CuZn25 alloy compared to pure Cu (Figure 4), namely a 2.4‐fold increase for Case A and a 3.4‐fold increase for Case B. In Case B, Γ increases systematically with Zn content, whereas in Case A, the apparent increase is largely driven by single data point (CuZn25), whereas CuZn10 and CuZn20 have similar values compared to Cu, such that a nearly constant Γ throughout the series cannot be excluded if CuZn25 is an outlier. The trend of increasing Γ with Zn content appears counterintuitive when compared to the experimental double‐layer capacitance measurements (Table 1), which show a decreasing trend in the order Cu > CuZn10 > CuZn20 > CuZn25. However, this downward trend primarily reflects a change in the specific capacitance of the material rather than a geometric reduction in available surface atoms. In our kinetic model Γ represents the areal concentration of sites capable of ${\mathrm{CO}}_{2}$ activation. The identified increase in Γ suggests that while the macroscopic capacitance decreases due to shifting electronic properties of the double layer, Zn alloying activates a higher fraction of the surface Cu atoms for ${\mathrm{CO}}_{2}$ adsorption. Crucially, this increase in Γ alone cannot explain the enhanced activity with Zn content. As demonstrated in our sensitivity analysis (Figures S22 and S23), increasing only the areal concentration of active sites reproduces higher currents but simultaneously suppresses the characteristic hysteresis observed in the CV experiments (Figure 1).

In summary, the contradictory trends for Cases A and B regarding the impact of Zn suggest fundamentally different mechanistic explanations for the enhanced activity of CuZn alloys. Case A emphasizes the role of Zn in the ${\mathrm{CO}}_{2}$ activation step, while Case B emphasizes the role of Zn on the formation and desorption of CO. To further evaluate these hypotheses, we carry out a detailed analysis of rates and coverages for both Cases, followed by DFT calculations to clarify Zn's mechanistic role at the atomistic level.

#### 2.2.1 Case A

Case A provides an excellent match to the experimental data and, therefore, is a plausible explanation for the observed enhancement in electrocatalytic activity with increasing Zn content. To gain a deeper mechanistic understanding of the predicted underlying kinetic improvements, we conducted a detailed kinetic analysis of Case A. This analysis focuses on examining the evolution of adsorption and desorption rates, reaction rates, as well as surface coverages of intermediates as functions of the applied potential during dynamic cycling.

Figure 5a–d presents the simulated reaction and sorption rates for a scan rate of 200 mV s^−1^ for Case A. Notably, the ${\mathrm{CO}}_{2}$ adsorption and reduction rates of all materials exhibit an S‐shaped curve, characterized by an initial region of low activity, followed by a gradual increase starting around −1.6 V. The activity then plateaus at potentials more negative than −2.2 V. In contrast, the formation and desorption rates of CO continue to increase throughout the forward sweep. This difference in the rate profile suggests that the initial ${\mathrm{CO}}_{2}$ activation steps, including ${\mathrm{CO}}_{2}$ adsorption and reduction to *CO_2_^−^, are rate limiting, while the subsequent CO formation and CO desorption rates are less kinetically constrained under these conditions. In terms of the effect of the Zn content, all four reaction steps exhibit rates that increase monotonically with the Zn content, which explains the experimentally observed increase in overall catalytic activity. Only CuZn20 deviates from this trend in the CO desorption rate (Figure 5d), showing an abnormally low magnitude and pronounced hysteresis. This can be explained by the strong outlier of its CO desorption rate constant shown in Figure 4b and is confirmed by simulations using linearly interpolated parameters for CuZn20 that do not show such a deviating trend (Figure S25).

Surface coverages reveal limitations, such as blocking by prevalent species or the absence of reactants for a given reaction step; therefore, we analyze the coverages of adsorbed intermediates *${\mathrm{CO}}_{2}$, *CO_2_^−^, *CO, and free sites (*) during CV (Figure 5e–g). The coverage of free sites decreases significantly from Cu to CuZn10 and further to CuZn25, indicating improved catalyst utilization with increasing Zn content. This decrease may also result from site blocking by strongly adsorbed intermediates, which could limit the overall activity. Again, CuZn20 exhibits the most pronounced decrease in free sites but displays a distinct profile and is therefore considered an outlier, as discussed previously.

${\mathrm{CO}}_{2}$ coverage ($\theta_{{\mathrm{CO}}_{2}}$) is generally low (below 5%) and drops sharply to almost zero at potentials below −1.6 V, suggesting that the supply of *${\mathrm{CO}}_{2}$ by adsorption is smaller than its consumption in the subsequent step, i.e., the adsorption of ${\mathrm{CO}}_{2}$, is rate limiting at high cathodic potentials, not only for Cu [38] but also for the CuZn alloys.

The coverage of *CO_2_^−^ is significantly increased for the CuZn alloys compared to pure Cu. It increases with Zn content (with CuZn20 as an outlier) and correlates with the observed increase in ${\mathrm{CO}}_{2}$RR activity with increasing Zn content. The increased *CO_2_^−^ coverage in the α‐CuZn alloys may be attributed to a stabilizing effect of the Zn alloy environment on *CO_2_^−^, as Zn is more oxyphilic than Cu [58]. Furthermore, the presence of Zn sites neighboring Cu steps has been reported to facilitate ${\mathrm{CO}}_{2}$ activation in thermal catalysis [31].

Our simulations of Case A predict negligible *CO coverage across the potential range studied for Cu, CuZn10, and CuZn25. This aligns with Fourier transform infrared spectroscopy (FTIR) studies of polycrystalline Cu, which reported the absence of infrared bands characteristic of adsorbed CO in acetonitrile electrolyte [14, 59]. CuZn20 is denoted as an outlier due to its significantly higher CO desorption rate constant.

To confirm the absence of mass transport limitations, the concentration profiles of dissolved ${\mathrm{CO}}_{2}$ at the electrode–electrolyte interface are given in (Figure 5h). ${\mathrm{CO}}_{2}$ concentration decreases at potentials below −2 V, with lower concentrations, indicating higher consumption rates, observed for alloys with higher Zn content. The slight increase in concentration from −0.8 to −2 V can be attributed to the slow diffusion of ${\mathrm{CO}}_{2}$ from the bulk, which replenishes the consumed ${\mathrm{CO}}_{2}$ during phases of low current density. Despite the high ${\mathrm{CO}}_{2}$ consumption rates, the ${\mathrm{CO}}_{2}$ concentration at the electrode remains above 92% of its saturation value throughout the potential window, indicating that mass transport limitations are negligible under the conditions studied. This conclusion is further supported by the nearly perfect overlap of consecutive simulated CV cycles with minimal drift. Furthermore, our experimental CVs recorded at scan rates between 50 and 300 mV s^−1^ demonstrate that the cathodic current continues to increase with scan rate and does not converge to a scan rate‐independent limiting current characteristic of a diffusion‐controlled regime (Figures S4–S7). This conclusion is also consistent with previous work on Cu in the same electrolyte, which found no ${\mathrm{CO}}_{2}$ depletion at the Cu surface under comparable conditions [38], and with McGregor's studies on similarly active Ag electrodes in aprotic media, where transport limitations were also ruled out. Steady‐state simulations extended to potentials beyond −2.4 V further confirm that the depletion of dissolved ${\mathrm{CO}}_{2}$, and thus a transport‐limited current, does not occur for the planar CuZn electrodes (Figure S21). However, for structured electrodes with a high specific area, such as meshes or nanoparticle‐based catalysts, transport limitations may arise, as shown in Figure S21.

In summary, for Case A, the improvements in the rates observed for the higher alloyed α‐CuZn catalysts can be primarily attributed to an improvement in ${\mathrm{CO}}_{2}$ activation. The observed increase in both ${\mathrm{CO}}_{2}$ adsorption and ${\mathrm{CO}}_{2}$ reduction rates, coupled with the enhanced coverage of *CO_2_^−^, suggests that Zn alloying facilitates the initial steps of the ${\mathrm{CO}}_{2}$ reduction pathway. The increased coverages do not appear to displace other adsorbed species, indicating improved utilization of the available sites.

#### 2.2.2 Case B

Case B contains significantly different kinetic parameters and therefore may offer an alternative mechanistic explanation for the enhanced electrocatalytic activity observed in α‐CuZn alloys.

The calculated reaction rates (Figure 6a–d) show that all steps exhibit increased rates with higher Zn content, mirroring the trends observed in Case A. The most significant increases with Zn content are observed in the ${\mathrm{CO}}_{2}$ reduction and CO formation rates, suggesting that Zn facilitates electron transfer in both these steps. The rates of ${\mathrm{CO}}_{2}$ adsorption and CO desorption exhibit less pronounced increases. Furthermore, compared to Case A, the CO desorption rate is slower, indicating that the desorption of CO may limit ${\mathrm{CO}}_{2}$RR.

The corresponding surface coverages (Figure 6e–h) exhibit significant differences compared to Case A. The percentage of free sites is considerably lower across the CuZn alloy series, remaining below 20% and approaching near‐zero values at potentials more negative than −2 V, with the exception of CuZn20, which again displays anomalous behavior. The coverage of ${\mathrm{CO}}_{2}$ is significantly higher compared to Case A, but also decreases at potentials more negative than −1.8 V, approaching zero between −2.4 and −2.5 V. This indicates that, in addition to CO desorption, ${\mathrm{CO}}_{2}$ adsorption also contributes to slow ${\mathrm{CO}}_{2}$ reduction kinetics.

*CO_2_^−^ is absent from the surface at high Zn content, i.e., CuZn20 and CuZn25, indicating that it reacts more rapidly than it is produced. However, at low Zn content, i.e., CuZn10 and Cu, a significant amount of adsorbed *CO_2_^−^ accumulates, which rapidly disappears at potentials below −2.0 V.

Finally, the CO coverage shows the most significant difference compared to Case A. While CO coverage was negligible in Case A, Case B predicts a rapid increase in CO coverage to approximately 80% below −1.6 V. This substantial CO coverage at elevated cathodic potentials, coupled with the fact that the estimated parameter value for the areal concentration of adsorption sites Γ in Case B is one order of magnitude smaller compared to Case A, suggests saturation of the surface with adsorbed CO during ${\mathrm{CO}}_{2}$RR. This effectively blocks a large fraction of the catalyst surface and limits the available sites for further ${\mathrm{CO}}_{2}$ conversion. Although the accumulation of CO on the surface is observed in all materials, higher alloyed CuZn materials appear to partially mitigate this effect through enhanced CO desorption rates. Furthermore, the complete removal of adsorbed CO during the anodic scan suggests that the high coverage by *CO stems from CO formed by ${\mathrm{CO}}_{2}$RR rather than re‐adsorption from solution. The high CO coverage predicted contrasts with the aforementioned reports of low CO coverage of Cu in acetonitrile by FTIR spectroscopy [14, 59]. However, experimental uncertainties due to the low sensitivity of FTIR for detecting CO bands in acetonitrile. In contrast, in aqueous electrolytes, Cu typically exhibits distinct IR bands for adsorbed CO species during ${\mathrm{CO}}_{2}$RR [60].

In summary, Case B suggests that the catalytic performance of Cu and CuZn alloys is limited by the saturation of the surface with CO exceeding 80% coverage. Although Zn may facilitate the electron transfer reactions, as evidenced by the enhanced ${\mathrm{CO}}_{2}$ reduction and CO formation rates, the primary mechanism for the improved activity of CuZn alloys, according to Case B, is the improved removal of adsorbed CO at elevated potentials.

#### 2.2.3 Assessing Adsorbed CO Accumulation

To assess the likelihood of CO poisoning, and thus of Case B, we performed CV experiments in CO‐saturated and 50:50 (v/v) CO:CO_2_‐saturated electrolyte on Cu and CuZn25. The experimental results are shown in Figure 7 along with the corresponding simulations based on Cases A and B.

**FIGURE 7:**
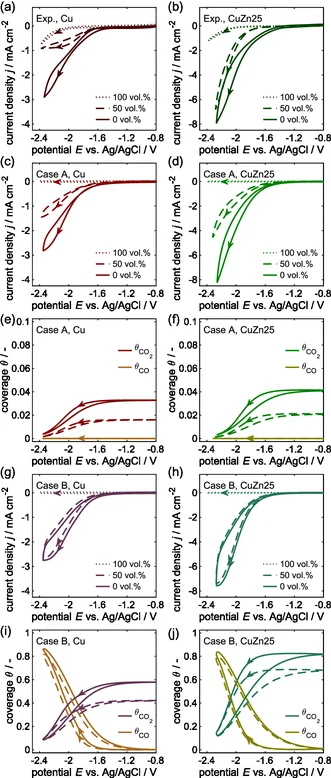
Experimental and simulated CVs and corresponding surface coverages on Cu and CuZn25 electrodes in aprotic 0.1 mol L^−1^ TBABF_4_ acetonitrile electrolyte at room temperature and a scan rate of 200 mV s^−1^ under CO atmosphere (100 vol.% CO), 50:50 (v/v) $\mathrm{CO}/{\mathrm{CO}}_{2}$ atmosphere (50vol.% CO), and ${\mathrm{CO}}_{2}$ atmosphere (0vol.% CO). Panels (a) and (b) show experimental CVs for Cu and CuZn25, respectively, panels (c) and (d) show Case A simulated CVs, and panels (g) and (h) show Case B simulated CVs. Panels (e) and (f) show Case A simulated surface coverages of $\theta_{{\mathrm{CO}}_{2}}$ and $\theta_{\mathrm{CO}}$ for Cu and CuZn25, respectively, while panels (i) and (j) show the corresponding coverages for Case B.

In the absence of ${\mathrm{CO}}_{2}$, i.e., 100 vol.% CO atmosphere, the CVs of Cu and CuZn25 exhibit negligible reduction currents until cathodic potentials below −2.0 V, where minor activity likely arises from the decomposition of acetonitrile rather than CO reduction, as there is no proton source [61]. We do not explicitly include electrolyte decomposition in the kinetic model, because the acetonitrile decomposition kinetics and pathway is highly complex as it depends on the gas atmosphere, e.g., for Ar‐ and CO_2_‐saturated conditions (for Ar CVs, see Figure S29) [22]. As such, the CVs cannot be corrected by subtracting the Ar contribution from the full spectrum. Consequently, simulations for both catalysts predict no activity in this regime. Although electrolyte decomposition may overlap with the measurements under ${\mathrm{CO}}_{2}$ atmosphere at potentials below −2.0 V, additional analysis shows that the mechanistic conclusions of our kinetic model remain unchanged even when accounting for acetonitrile reduction. As detailed in Section S11, the kinetic parameters for Case A were re‐parameterized using background‐corrected data, which was obtained by subtracting the CVs recorded under 100 vol.% CO from the original ${\mathrm{CO}}_{2}$ CVs. This reevaluation yielded the same mechanistic interpretation and kinetic trends as the original model (see Figures S26–S28), confirming that our conclusions are robust with respect to potential electrolyte decomposition.

The 50:50 (v/v) CO:${\mathrm{CO}}_{2}$ experiment shows no clear evidence of CO poisoning on CuZn25; the observed current density was approximately 14% lower compared to ${\mathrm{CO}}_{2}$ atmosphere. This is attributed to the reduced ${\mathrm{CO}}_{2}$ partial pressure and, thus, ${\mathrm{CO}}_{2}$ concentration, although a shift in the onset to more negative potentials is observed. In contrast, Cu shows a more substantial decrease in current density under 50:50 (v/v) CO:${\mathrm{CO}}_{2}$ conditions. This may be attributed to CO poisoning or to the lower ${\mathrm{CO}}_{2}$ concentration, which has a stronger effect on ${\mathrm{CO}}_{2}$ adsorption on pure Cu. CO poisoning is unlikely here as well, as molecular dynamics simulations showed facile CO desorption from Cu into bulk acetonitrile solution [62].

Simulations confirm that Case A reproduces the 50:50 (v/v) CO:${\mathrm{CO}}_{2}$ experiment much better, while Case B fails to reproduce the experimental trend. As shown in Figure 7e and f, Case A captures the observed gas‐composition dependence of the CV via reduced ${\mathrm{CO}}_{2}$ coverage, i.e., lower $\theta_{{\mathrm{CO}}_{2}}$, rather than changes in CO coverage, and shows no signs of CO poisoning, as $\theta_{\mathrm{CO}}$ remains negligible for all gas mixtures. In contrast, the simulated CVs based on Case B are largely insensitive to variations in CO and ${\mathrm{CO}}_{2}$ concentrations, contradicting the experiments and thus rendering the parameter set of Case B implausible.

## 3 Density Functional Theory Results: Potential‐Dependent Adsorption

To corroborate the indications from the kinetic analysis favoring Case A over Case B for the observed enhancement in ${\mathrm{CO}}_{2}$ reduction activity, we performed DFT calculations probing ${\mathrm{CO}}_{2}$ and CO adsorption energies on Cu(111) and ${\mathrm{Cu}}_{3}\mathrm{Zn}$(111) surfaces. We chose the ${\mathrm{Cu}}_{3}\mathrm{Zn}$(111) surface as a representative model for the α‐CuZn alloy surfaces as this provides a reasonable model with $\frac{1}{4}$ Zn atoms in the first surface layer. We also note that we expect Zn to be present in the surface layer due to its segregation energy in copper (see Figure S13).

The free energies of adsorption are shown in Table 3 (enthalpies, see Table S2). ${\mathrm{CO}}_{2}$ is physisorbed on all investigated sites with a metal–carbon distance above 3.5 Å and an O─C─O angle of 180°. In contrast, CO is chemisorbed at the on‐top sites on Cu with a metal–carbon distance of 1.9 Å. On Zn sites, CO exhibits weaker adsorption (physisorbed), characterized by an elongated metal–carbon distance (>2.1 Å). Additional analysis of the dispersion contribution to the adsorption energies further supports this conclusion (Table S3). This suggest that CO potentially blocks the surface of pure Cu, seemingly consistent with Case B of our kinetic modeling, whereas for the ${\mathrm{Cu}}_{3}\mathrm{Zn}$ alloy, such a problem should not arise. Indeed, the calculated coverage difference derived from the corresponding free‐energy differences amounts to about three orders of magnitude between Cu and ${\mathrm{Cu}}_{3}\mathrm{Zn}$ (refer to the Supporting Information).

**TABLE 3:** DFT‐calculated Gibbs free energies $\Delta G_{\mathrm{ads}}$ at 298.15 K of ${\mathrm{CO}}_{2}$ and CO adsorption on the top sites in vacuum.

Surface	Site	**Δ*G*** **_ads_** **,** **eV**	
		* **CO** **_2_**	* **CO**
Cu(111)	Cu	+0.19	−0.02
Cu_3_Zn(111)	Cu	+0.25	+0.16
	Zn	+0.34	+0.65

To move toward a more realistic description of the electrochemical interface, sawtooth slab calculations were performed (see Supporting Information for details). These preliminary results indicate that Zn exhibits a stronger affinity toward the oxygen atoms of ${\mathrm{CO}}_{2}$, leading to a reduced adsorbate–surface distance. This may enhance the reduction rate by increasing the orbital overlap between the electrode states and ${\mathrm{CO}}_{2}$. However, no true chemisorbed state is observed in this simplified vacuum model as the interaction remains strictly governed by dispersion forces (Table S3). This absence of spontaneous chemisorption, contrary to experimental observations, indicates that a more complete representation of the electrochemical interface is required to accurately capture the active state.

### 3.1 Grand Canonical Approach

The simulations in vacuum are a rather simplified model of an electrochemical interface. A step toward improving this approach is to include an implicit solvent and explicitly charge the interface. As a result, GC adsorption energies, i.e., the constant‐potential free energy Ω, of ${\mathrm{CO}}_{2}$ and CO were obtained (Figure 8). The presence of the solvent stabilizes the chemisorbed state on the surface, characterized by a reduction of the O–C–O angle to 130° and a decrease in the distance between the surface and the molecule to 2.1 Å. This chemical binding can occur either through a single C─M bond or through two bonds, C─M and O─M, giving rise to two chemisorption modes of ${\mathrm{CO}}_{2}$, denoted as *${\mathrm{CO}}_{2}$ and *O‐*CO, respectively, as shown in the inset of Figure 8a. This observation is consistent with previous reports for aqueous electrochemical environments [63]. In both Cases, the stabilization is caused by the charge transferred from the slab to ${\mathrm{CO}}_{2}$, making it negatively charged. The transferred charge Δq therefore depends on the applied potential (Figure 8d).

**FIGURE 8:**
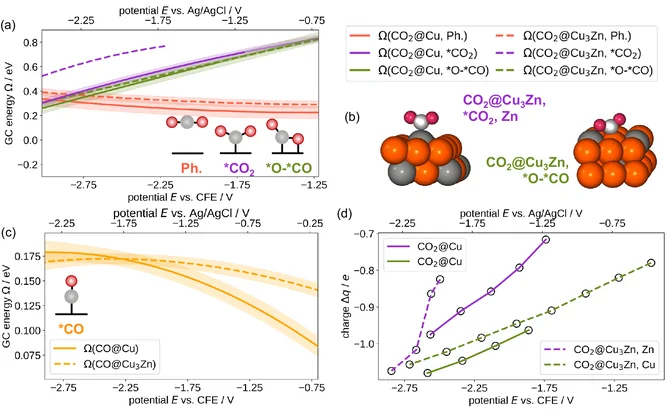
VASPsol Results. (a,c) GC adsorption energies of ${\mathrm{CO}}_{2}$ and CO on Cu and ${\mathrm{Cu}}_{3}\mathrm{Zn}$ surfaces. The insets schematically show the main modes of adsorption (Ph.: physisorbed). The upper x‐axis indicates the potential values versus Ag/AgCl, derived from the experimentally determined ferrocene redox potential. The light sections of the lines indicate regions where this type of adsorption has not been observed. 2σ confidence intervals shown as shaded regions. (b) Structure of chemisorbed ${\mathrm{CO}}_{2}$ on ${\mathrm{Cu}}_{3}\mathrm{Zn}$ (the slab was truncated for clarity). (d) Transferred charge Δq to the ${\mathrm{CO}}_{2}$ molecule in the chemisorbed state as a function of the applied potential on both surfaces, obtained with Bader charge analysis.

Based on the assumption that electroactivation (in this Case, chemisorption) of ${\mathrm{CO}}_{2}$ is the rate‐limiting step of electroreduction [64, 65], we therefore consider the intersection between the physisorbed and chemisorbed states as the upper limit of onset potential for ${\mathrm{CO}}_{2}$ activation. This yields the intersection potentials of −2.43 and −2.33 V versus Ag/AgCl for Cu, and −2.72 and −2.27 V versus Ag/AgCl for ${\mathrm{Cu}}_{3}\mathrm{Zn}$, corresponding to the one‐bond and two‐bond modes, respectively. While this observation of ${\mathrm{CO}}_{2}$ activation is consistent with Case A, the calculated difference between Cu and ${\mathrm{Cu}}_{3}\mathrm{Zn}$ is within the error of DFT, giving no clear trend.

Moreover, although the one‐bond adsorption mode of ${\mathrm{CO}}_{2}$ on ${\mathrm{Cu}}_{3}\mathrm{Zn}$ reaches its intersection potential relatively late, it becomes more favorable than all other modes at very high overpotentials (Figure 8a). This additional adsorption mode could further enhance the ${\mathrm{CO}}_{2}\mathrm{RR}$ rate at very negative potentials. An additional advantage of this scenario is that CO does not adsorb on zinc, which enables the rapid release of the reduction product and provides access for new ${\mathrm{CO}}_{2}$ molecules.

CO adsorption exhibits a slightly more pronounced curvature on the Cu surface (Figure 8c), with the adsorption energy of CO displaying a convex parabolic behavior in agreement with Cheng et al. [66]. At relevant cathodic potentials, below −1.6 V versus Ag/AgCl, the adsorption energies remain comparable between Cu and ${\mathrm{Cu}}_{3}\mathrm{Zn}$ with an average difference of 0.02 eV, indicating that alloying has a negligible influence on CO binding and desorption, and thus cannot explain the activity enhancement proposed by Case B. We note that another recent study reports an opposite curvature of CO adsorption energetics [67], where CO adsorption initially stabilizes with increasingly negative potential before destabilizing again. These contrasting reports suggest that our CO adsorption results should be interpreted with caution.

Nevertheless, we can estimate the contributions to the enhancement of ${\mathrm{CO}}_{2}\mathrm{RR}$ from ${\mathrm{CO}}_{2}$ activation and CO desorption by calculating the difference between the adsorption energy curves on ${\mathrm{Cu}}_{3}\mathrm{Zn}$ and Cu surfaces as $\Delta \Omega ({\mathrm{CO}}_{2}/\mathrm{CO})=\Omega ({\mathrm{CO}}_{2}/\text{CO@}{\mathrm{Cu}}_{3}\mathrm{Zn})-\Omega ({\mathrm{CO}}_{2}/\text{CO@Cu})$ for ${\mathrm{CO}}_{2}$ and CO, respectively (Figure 9), where the *O–*CO configuration is used for $\Omega ({\mathrm{CO}}_{2}\text{@Cu})$, as it is the most favorable conformation on Cu. As shown in Figure 9, the difference in the ${\mathrm{CO}}_{2}$ adsorption energetics is slightly more pronounced over a broad potential range, although no strong effect of Zn is observed, whereas the corresponding changes in CO adsorption remain small.

**FIGURE 9:**
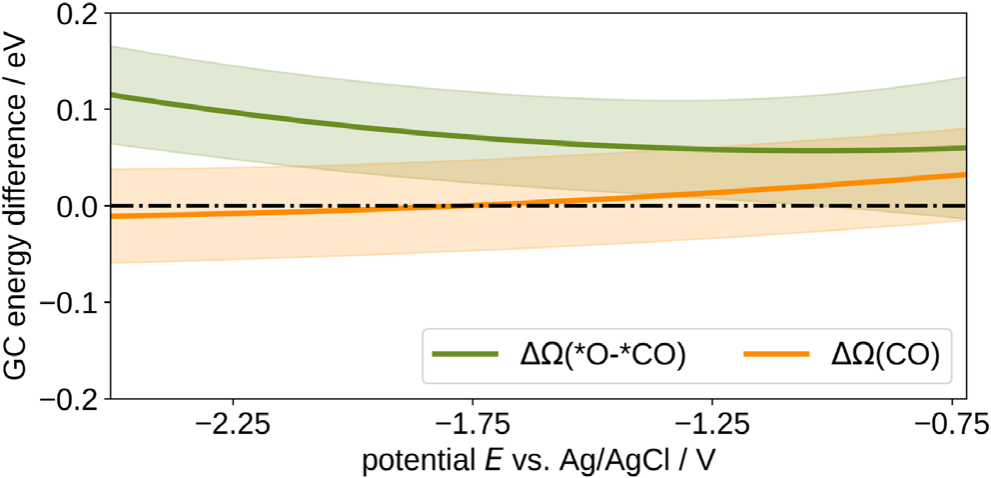
GC adsorption energy difference for ${\mathrm{CO}}_{2}$ and CO on ${\mathrm{Cu}}_{3}\mathrm{Zn}$ versus the most stable conformation on Cu, namely the two‐bond chemisorption of ${\mathrm{CO}}_{2}$ and on‐top adsorption of CO. The same color scheme as in Figure 8 is used.

## 4 Conclusion

This study presented a comprehensive investigation of α‐CuZn alloy catalysts for ${\mathrm{CO}}_{2}$ electroreduction in an aprotic acetonitrile‐based electrolyte, combining electrochemical experiments, kinetic modeling, and DFT calculations to unravel the role of Zn in enhancing catalytic performance.

Experimental cyclic and LSV measurements demonstrated a significant increase in catalytic activity with increasing Zn content in the α‐CuZn alloys, with CuZn25 exhibiting the highest current density across all measurement conditions. This enhancement was accompanied by a shift in the onset potential toward more positive potentials and pronounced hysteresis, indicating the involvement of adsorbed reaction intermediates. The performance of pure Zn, by contrast, remained poor, with a delayed onset and lower currents, suggesting that Zn acts as a promoter when alloyed with Cu rather than as an independent active site.

To interpret these trends, we applied a dynamic kinetic model describing ${\mathrm{CO}}_{2}$ activation, electron transfer, and product desorption steps. Two plausible mechanistic Cases were identified. In Case A, Zn was found to enhance ${\mathrm{CO}}_{2}$ activation by increasing the rate of the initial electron transfer step to *CO_2_^−^. This Case predicted improved reaction rates and intermediate coverages in agreement with experimental observations. In Case B, the model attributed the activity enhancement primarily to facilitated CO desorption. However, it also predicted excessive CO coverage that was inconsistent with spectroscopic and molecular dynamics simulation studies.

Finally, DFT calculations provided atomistic support for Case A over Case B by demonstrating that Zn incorporation into the Cu lattice stabilizes ${\mathrm{CO}}_{2}$ chemisorption to ${\mathrm{CO}}_{2}^{-}$ at lower overpotentials, while showing similar CO adsorption energies for Cu and ${\mathrm{Cu}}_{3}\mathrm{Zn}$ at relevant cathodic potentials. A bidentate binding configuration of ${\mathrm{CO}}_{2}$ was identified as the most stable at intermediate potentials. At elevated overpotentials, a further monodentate binding mode located on Zn has been identified.

Case A is therefore favored over Case B based on the combined agreement with CV hysteresis, scan‐rate dependence, CO atmosphere experiments, and DFT adsorption energetics. While Case B predicts excessive CO accumulation and weak gas‐composition dependence that are not observed experimentally, Case A correctly attributes the promotional effect of Zn to facilitated ${\mathrm{CO}}_{2}$ activation.

The combined experimental, kinetic, and computational insights presented here establish a coherent mechanistic understanding of Zn promotion in CuZn alloys. While this advancement improves ${\mathrm{CO}}_{2}$ activation, ${\mathrm{CO}}_{2}$ adsorption remains relatively slow, limiting overall rates and suggesting opportunities for further system optimization that combine Zn‐promoted Cu alloys with high‐surface‐area architectures or elevated ${\mathrm{CO}}_{2}$ pressures.

## 5 Experimental Section

### 5.1 Electrode Preparation

CuZn electrodes were prepared by machining commercially purchased metal rods into planar disk electrodes; no alloy synthesis was performed in‐house. Cu and Zn rods (both 99.99%, Thermo Fisher Scientific Inc.) and α‐brass metal rods with Zn mass contents of 10%, 20% and 25% (99.99%, Rich Special Materials Co., Ltd.) were used. The metal rods were lathed to a uniform diameter of 5 mm and cut into cylindrical pieces with a height of 4 mm. For initial preparation, the top side of the disk electrode was sanded with 2000‐grit SiC paper and sequentially polished with 9, 3, and 1 μm water‐based diamond suspensions (Struers GmbH). Afterward, the disks were mounted in PEEK shrouds (E5TQPK, Pine Research Instrumentation, Inc.). To minimize the influence of surface oxidation and remove the native oxide layer, the disk electrodes were freshly polished before each measurement with 0.25 μm diamond paste (Kemet International Ltd.), rinsed with ultrapure water, ultrasonicated for 5 min in ultrapure water, rinsed again with ultrapure water, and finally dried by blowing them with compressed air.

### 5.2 Electrochemical Measurements

Measurements were conducted in a 250‐mL three‐electrode glass cell setup equipped with a Ag/AgCl (${\mathrm{KCl}}_{\mathrm{sat}}$ in $H_{2}O$) reference electrode (Sensortechnik Meinsberg) and a Pt‐coil counter electrode (∅ 0.5 mm, Heraeus Precious Metals GmbH & Co. KG) (Figure S2). A volume of 150 mL electrolyte solution consisting of 0.1 mol L^−1^ tetrabutylammonium tetrafluoroborate (${\mathrm{TBABF}}_{4}$) in acetonitrile was prepared using ${\mathrm{TBABF}}_{4}$ (98%, abcr GmbH) and acetonitrile (≥99.9% max. $100\;\text{ppm}\;H_{2}O$, VWR Chemicals). To ensure thermodynamic consistency in the aprotic electrolyte, the Ag/AgCl reference electrode was calibrated against the ferrocene/ferrocenium (${\text{Fc}}^{0}/{\text{Fc}}^{+}$) redox couple (see Figure S3). The electrolyte was saturated with ${\mathrm{CO}}_{2}$ gas (≥99.995%, Air Liquide Deutschland GmbH) by bubbling at a constant flow rate of 60 mL min^−1^ for 15 min through a fritted bubbler directly before the measurements. During the measurements, a ${\mathrm{CO}}_{2}$ gas atmosphere was maintained in the headspace above the solution by using a bypass flow of ${\mathrm{CO}}_{2}$ gas in the headspace at a rate of 30 mL min^−1^. For the CO and Ar experiments, CO gas (≥99.5%, Air Liquide Deutschland GmbH) or Ar gas (≥99.999%, Air Liquide Deutschland GmbH) was used, respectively, to saturate the electrolyte solution and headspace, following the same procedure as for ${\mathrm{CO}}_{2}$. At the start of each measurement, a preconditioning step consisting of 20 consecutive CV cycles recorded at 100 mV s^−1^ between −0.8 and −2.4 V versus Ag/AgCl was performed. This procedure serves to stabilize the interface and minimize the influence of residual water. Subsequently, CV measurements were conducted at scan rates of 300, 200, 150, 100, 75, and 50 mV s^−1^ in descending order. LSV was performed directly afterward at a scan rate of 1 mV s^−1^. For both CV and LSV, the potential range was set from −0.8 to −2.4 V versus Ag/AgCl to avoid electrode oxidation and electrolyte degradation. As we observed no transport limitation in our previous study [38], no rotation was applied. All electrochemical measurements were performed at ambient conditions (25 °C, 101.3 kPa) using a Gamry Interface 1010E potentiostat and were repeated three times in total on the same electrode. For each repetition, fresh electrolyte solution was used, and the electrode was freshly polished using 0.25 μm diamond paste, cleaned, and dried as described above. After the preconditioning step, the uncompensated resistance *R*_u_ was determined by identifying the high‐frequency resistance (100 kHz to 1 MHz, phase angle φ=0°) using potentiostatic EIS at −0.8 V with an input amplitude of 10 mV. Post‐run *iR* correction was applied to all data.

## 6 Computational Methods

### 6.1 Kinetic Model Details

#### 6.1.1 Model Description

The dynamic kinetic model describes the processes at the cathode half‐cell during ${\mathrm{CO}}_{2}$RR at a planar electrode immersed in a stagnant, ${\mathrm{CO}}_{2}$‐saturated 0.1 mol L^−1^ ${\mathrm{TBABF}}_{4}$ acetonitrile electrolyte at room temperature and atmospheric pressure. Consistent with previous reports on Cu and α‐CuZn catalysts in acetonitrile, which show high selectivity toward CO with only trace oxalate formation [37], the model assumes that the measured Faradaic current is dominated by the ${\mathrm{CO}}_{2}$‐to‐CO pathway. This is further supported by our control measurements in Argon (Figure S29), which exhibit significantly lower current densities than those recorded under ${\mathrm{CO}}_{2}$. To capture the influence of concentration gradients and mass transport within the simulation domain, the model contains one‐dimensional diffusive transport of CO, ${\mathrm{CO}}_{2}$, and CO_3_^2−^ within the electrolyte's Nernst diffusion layer between the electrode surface and the ideally mixed bulk electrolyte. At the electrode, reactions, adsorption, and desorption processes occur, as detailed in our previous work [38]. All calculations were performed using the ode15s solver in MATLAB 2024b.

In our previous microkinetic modeling study of the oxygen evolution reaction (OER) on binary Ir–Ru catalysts, two distinct active sites were assumed because both Ir and Ru exhibited significant OER activity as pure metal oxides and showed distinct characteristic CV features [41]. In contrast, for the ${\mathrm{CO}}_{2}$RR on CuZn alloys, Zn alone shows no activity above −2.0 V (see Figure 1), and both Cu and Zn individually exhibit lower activity than the CuZn alloys. Therefore, the catalytic behavior of the CuZn alloys cannot be described as a linear combination of Cu and Zn, indicating a synergistic promoting effect. Since the CV profiles of the CuZn alloys closely resemble those of pure Cu, albeit with enhanced activity, our modeling approach considers only Cu sites on the surface to be active for ${\mathrm{CO}}_{2}$RR, while Zn's role is incorporated indirectly through its influence on the kinetic parameters. This approach provides valuable insights into surface coverages and thus represents an important first step in the kinetic modeling of this system.

We adopted the mechanism proposed by Amatore and Savéant with the following simplification [38, 68]: ${\mathrm{CO}}_{2}$ attachment to the radical anion, subsequent electron transfer, and carbonate detachment are treated as a single effective step. For this process, the model thus utilizes an effective step representation rather than a fully elementary description as the carbonic formic anhydride radical anion is not resolved as a separate intermediate. This is justified because the species has not been experimentally confirmed and is therefore considered a short‐lived transition state. The implemented reaction mechanism thus consists of the following steps

1. ${\mathrm{CO}}_{2}$ adsorption: Reversible adsorption of ${\mathrm{CO}}_{2}$ on free active surface sites of the catalyst (∗).

(I)

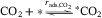

2. ${\mathrm{CO}}_{2}$ reduction: Electron transfer from the electrode to adsorbed *${\mathrm{CO}}_{2}$ forming the *CO_2_^−^ radical anion.

(II)

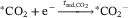

3. Electrochemical disproportionation: Electrochemical reduction of *CO_2_^−^ and a nearby solution‐phase ${\mathrm{CO}}_{2}$ to form *CO and CO_3_^2−^.

(III)

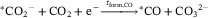

4. CO desorption: Desorption of *CO from the electrode surface.

(IV)

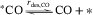




Electrochemical reaction steps are assumed to be irreversible as no positive currents were observed in the potential range studied. Previous studies have suggested that the initial step in ${\mathrm{CO}}_{2}$ activation involves the direct conversion of ${\mathrm{CO}}_{2}$ from solution to the adsorbed *CO_2_^*δ*−^, bypassing the formation of an uncharged *${\mathrm{CO}}_{2}$ adsorbate [25, 68, 69]. This is also supported by our DFT calculations, which show that only upon the application of a sufficiently negative potential, ${\mathrm{CO}}_{2}$ chemisorbs in the form of *CO_2_^*δ*−^ (Figure 8a,d). Recognizing that the activation of ${\mathrm{CO}}_{2}$ involves a shared electron density between ${\mathrm{CO}}_{2}$ and the electrode surface, we chose to model the adsorption and reduction as distinct steps in this work, analogous to our previous work [38]. This simplification avoids the complexity of incorporating fractional electron transfer within our kinetic model, which would have implications for charge balance. It is important to note that the two adsorbed states of ${\mathrm{CO}}_{2}$ in our model (*${\mathrm{CO}}_{2}$ and *CO_2_^−^) reflect different degrees of electron transfer and do not necessarily correspond to distinct physisorbed or chemisorbed states. Consequently, caution must be exercised when determining the rate‐determining step if it involves either of these closely related adsorbed states.

The rate equations corresponding to the reaction and sorption processes in Equations (I)–(IV) are expressed as follows

(1) $$\begin{array}{cc} & r_{{\mathrm{ads},\mathrm{CO}}_{2}}=k_{{\text{ads,CO}}_{2}}\;a_{{\mathrm{CO}}_{2}}\;\theta_{*}-k_{{\mathrm{des},\mathrm{CO}}_{2}}\;\theta_{{\mathrm{CO}}_{2}} \end{array}$$

(2) $$\begin{array}{cc} & r_{\text{red,}{\mathrm{CO}}_{2}}=k_{{\text{red,CO}}_{2}}\;\theta_{{\mathrm{CO}}_{2}}\exp\left(-\frac{\left(1-\alpha_{{\text{red,CO}}_{2}}\right)\;FE}{RT}\right) \end{array}$$

(3) $$\begin{array}{cc} & r_{\text{form,CO}}=k_{\text{form,CO}}\;a_{{\mathrm{CO}}_{2}}\;\theta_{{\mathrm{CO}}_{2}^{-}}\exp\left(-\frac{\left(1-\alpha_{\text{form,CO}}\right)\;FE}{RT}\right) \end{array}$$

(4) $$\begin{array}{cc} & r_{\text{des,CO}}=k_{\text{des,CO}}\;\theta_{\mathrm{CO}}-k_{\text{ads,CO}}\;a_{\text{CO}}\;\theta_{*} \end{array}$$

where $\theta_{i}$ represents the surface coverage, $\theta_{*}$ represents the fraction of surface area uncovered by any adsorbate, and $a_{i}$ represents the local activity in the solution of species i, assumed to be ideal ($a_{i}=c_{i}/c^{0}$ with $c^{0}=1\;\mathrm{Mol}\;L$). For the electrochemical steps, we assume irreversible Tafel kinetics relating the rate to the potential E versus Ag/AgCl at the electrode–electrolyte interface, with the Faraday constant F and the product of temperature and the universal gas constant RT. The parameters $\alpha_{\mathrm{red},{\mathrm{CO}}_{2}}$ and $\alpha_{\text{form,CO}}$ represent the charge transfer coefficients of each electrochemical step and were treated as free, bounded optimization variables in the interval 0.1–0.9. Sensitivity analysis (Figures S22 and S23) reveals that the model is extremely sensitive to these coefficients due to their presence in the exponential term of the rate equations. The observation that these values remain nearly constant across the CuZn series suggests that the transition state symmetry for ${\mathrm{CO}}_{2}$ activation is a robust characteristic of the Cu‐based reaction environment that remains largely unaffected by Zn alloying. The rate equations for the electrochemical steps (Equations (2) and (3)) are expressed as functions of the electrode potential E, rather than the overpotential $\eta =E-E_{\text{eq}}$. Since the equilibrium potentials $E_{\text{eq}}$ for the individual elementary steps are not explicitly known, the equilibrium contribution is incorporated into the effective rate constants $k_{i}$. This approach follows a well‐established practice [40, 70, 71] and reduces the number of unknown parameters while accurately capturing the potential‐dependent kinetic response observed in the CV experiments. Consequently, the reported values for $k_{i}$ should be interpreted as effective rate constants rather than fundamental exchange rate constants.

Mass transport of reactive species in the electrolyte between the electrode surface and the bulk electrolyte is described by a one‐dimensional diffusion model within the Nernst diffusion layer. Migration of carbonate in the electric field is neglected because an excess of supporting electrolyte (0.1 mol L^−1^) is employed [72], and only low carbonate formation rates are observed. The thickness of this layer, δ, is set to 6 mm to ensure that the arising concentration gradients are fully contained within the model domain for all experimental conditions. While this value may appear large, it is important to consider that the experiments were conducted in a stagnant solution without any forced convection. Under these conditions, the diffusion layer expands continuously over time and can be estimated based on the diffusion coefficient $D_{i}$ of species i and the experimental duration t using the following approximation, $\delta \approx \sqrt{D_{i}t}$ [73]. The species in the solution are balanced as follows

(5) $$\begin{array}{cc} \frac{\partial c_{{\mathrm{CO}}_{\text{2},n}}}{\partial t} & =\;D_{{\mathrm{CO}}_{2}}\frac{\partial^{2}c_{{\mathrm{CO}}_{2},n}(x,t)}{\partial x^{2}} \end{array}$$

(6) $$\begin{array}{cc} \frac{\partial c_{\text{CO},n}}{\partial t} & =\;D_{\text{CO}}\frac{\partial^{2}c_{\text{CO},n}(x,t)}{\partial x^{2}} \end{array}$$

(7) $$\begin{array}{cc} \frac{\partial c_{{\mathrm{CO}}_{3}^{2-}\text{,n}}}{\partial t} & =\;D_{{\mathrm{CO}}_{3}^{2-}}\frac{\partial^{2}c_{{\mathrm{CO}}_{3}^{2-}\text{,n}}(x,t)}{\partial x^{2}} \end{array}$$

We assume that ${\mathrm{CO}}_{2}$ radical anion remains adsorbed at the electrode surface (*CO_2_^−^) and does not diffuse into the electrolyte as a solvated ${\mathrm{CO}}_{2}^{•-}$ radical anion, consistent with reports showing no detectable or, at most, only trace oxalate formation on polycrystalline Cu [14, 59, 69] and CuZn [37].

The species balance for surface species is then given by

(8) $$\begin{array}{cc} \Gamma \frac{\text{d}\theta_{*}}{\text{d}t} & =\;-r_{{\mathrm{ads},\mathrm{CO}}_{2}}+r_{\mathrm{des},\mathrm{CO}} \end{array}$$

(9) $$\begin{array}{cc} \Gamma \frac{\text{d}\theta_{{\mathrm{CO}}_{2}}}{\text{d}t} & =\;r_{{\mathrm{ads},\mathrm{CO}}_{2}}-r_{{\mathrm{red},\mathrm{CO}}_{2}} \end{array}$$

(10) $$\begin{array}{cc} \Gamma \frac{\text{d}\theta_{{\mathrm{CO}}_{2}^{-}}}{\text{d}t} & =\;r_{{\mathrm{red},\mathrm{CO}}_{2}}-r_{\text{form,CO}} \end{array}$$

(11) $$\begin{array}{cc} \Gamma \frac{\text{d}\theta_{\mathrm{CO}}}{\text{d}t} & =\;r_{\text{form,CO}}-r_{\text{des,CO}} \end{array}$$

with Γ referring to the areal concentration of adsorption sites. Our kinetic model does not explicitly distinguish between specific Cu and Zn sites or different crystallographic facets on the electrode surface. Instead, Γ represents all available sites, regardless of their identity, and can therefore be seen as an effective, i.e., lumped, parameter. This simplification is justified as we assume that Zn atoms are incorporated into the Cu lattice, effectively replacing Cu atoms and allowing us to treat the alloy as a homogeneous surface. Furthermore, pure Zn exhibited poor catalytic activity for ${\mathrm{CO}}_{2}$ reduction, making it unsuitable as a reference for the behavior of Zn sites if we were to treat them separately.

At t=0, the liquid phase is homogeneously saturated with ${\mathrm{CO}}_{2}$ ($c_{{\mathrm{CO}}_{2},0}=\;0.278\;\mathrm{mol}\;L^{-1}$) and is in equilibrium with the adsorbed ${\mathrm{CO}}_{2}$ coverage, while the concentrations of CO and carbonate, as well as the surface coverages of *CO_2_^−^ and *CO, are set to zero. The bulk electrolyte boundary (x=δ) is open, with constant bulk concentrations maintained by assuming perfect mixing in a large reservoir, thereby permitting bidirectional diffusive flux. The electrode boundary (x=0) is impermeable to diffusion (no‐flux condition) but permits all surface adsorption, reaction, and desorption processes.

Charge conservation at the electrode surface is maintained through a balance between the accumulation of charge in the electrical double layer, the total current density $j_{\text{total}}$, and the Faradaic current density calculated from Faraday's law

(12) $$C_{\text{dl}}\frac{\text{d}E}{\text{d}t}=j_{\text{total}}+F(r_{{\text{red,CO}}_{2}}+r_{\text{form,CO}})$$

where $C_{\text{dl}}$ refers to the areal double‐layer capacitance of the polished electrode and E refers to the local electrode potential. The total current density $j_{\text{total}}$ is calculated via Ohm's law from the potential drop due to the uncompensated internal resistance $R_{u}$, which was measured via in situ EIS.

(13) $$E=E_{\text{ext}}\;-\;j_{\text{total}}\;A\;R_{u}$$

The externally applied potential $E_{\text{ext}}$ follows the temporal sawtooth profile of the respective CV experiment. By solving Equation (13) for the total current density $j_{\text{total}}$ and substituting it into the charge balance (Equation (12)), the model couples the Ohmic losses with the interface kinetics, allowing the solver to resolve the transient interface potential E and the resulting currents.

#### 6.1.2 Model Parameterization

Separate sets for each catalyst (Cu, CuZn10, CuZn20, and CuZn25) of the following nine parameters were determined through a multistep optimization procedure using the CV measurements as described below: $k_{\mathrm{ads},{\mathrm{CO}}_{2}}$, $k_{\mathrm{des},{\mathrm{CO}}_{2}}$, $k_{\mathrm{red},{\mathrm{CO}}_{2}}$, $k_{\text{form,CO}}$, $k_{\text{des,CO}}$, $k_{\text{ads,CO}}$, $\alpha_{\mathrm{red},{\mathrm{CO}}_{2}}$, $\alpha_{\text{form,CO}}$, and Γ.

Parameter identification was performed using a multistage global‐to‐local optimization strategy. Starting with Cu, a global search was conducted using MATLAB 2024b's particle swarm optimization algorithm. The initial swarm consisted of 90 randomly initialized particles, corresponding to ten times the number of optimization parameters. To ensure a comprehensive exploration of the parameter space, this process was repeated 100 times from independent, uniformly distributed random starting populations, representing a total of 9000 unique starting trajectories. Each run was carried out for a maximum of 18,000 iterations or until a convergence criterion was met, defined as a relative change in the objective function (Equation (14) ) of less than 10^−6^ over 20 consecutive iterations.

The 20 best‐performing parameter sets from the global search were subsequently refined using MATLAB 2024b's pattern‐search algorithm, eventually converging to the two distinct local minima discussed as Case A and Case B. These two final parameter sets effectively reproduced the experimental CV data for Cu and were selected for further analysis.

Subsequently, the identified parameter sets for Cu in Cases A and B were used as the initial parameters for a sequence of local optimizations again using MATLAB 2024b's pattern search algorithm for each CuZn alloy composition in order of increasing Zn content: Cu → CuZn10 → CuZn20 → CuZn25. In each step, the optimized parameter set from the preceding composition served as the initial guess.

CVs from three independent experiments were interpolated to a common time base of 640 equally spaced data points (1 point every 5 mV) using linear interpolation and averaged to obtain mean CVs for model parameterization (Figures S4–S7).

To mitigate the risk of overfitting while capturing processes across different time scales, scan rates of 200 and 50 mV s^−1^ were used for model parameterization (training data). The remaining scan rates (300, 150, 100, and 75 mV s^−1^) were used for model validation.

The objective function $f_{\text{obj}}$ for the optimization was the normalized root mean squared error between the simulated and experimentally determined current densities.

(14) $$f_{\text{obj}}=\frac{1}{N}\sum_{\nu =1}^{N}\frac{\sqrt{\frac{1}{N}\sum_{n=1}^{N}{\left(j_{\text{sim},\nu ,n}-j_{\text{exp},\nu ,n}\right)}^{2}}}{\max(|j_{\text{exp,}\nu }|)}$$

where N is the number of scan rates used for parameter identification, N is the number of data points, and $j_{\text{sim},n}$ and $j_{\text{exp},n}$ are the corresponding simulated and experimental values of the current density at each data point during the third CV cycle.

The normalization of the data by the maximum current density observed during that specific experiment ensured a fair comparison across different scan rates and alloy compositions, where the current density varies significantly. All parameter optimizations were performed using MATLAB 2024b.

#### 6.1.3 Density Functional Theory Calculation Details

DFT calculations with periodic boundary conditions were conducted using the Vienna Ab initio Simulation Package (VASP) version 6.3.0 [74, 75]. The calculations employed the nonlocal BEEF–vdW exchange–correlation functional [76, 77] and projector‐augmented wave pseudopotentials [78, 79]. A 4 × 4 four‐layer slab of (111) surface and an energy cutoff of 450 eV were used, in connection with a 3 × 3 × 1 *Γ*‐centered *k*‐point mesh, which we found to be well converged (Figure S12). The applied sawtooth potential, ranging from 0.25 to −1.00 V Å^−1^, follows the VASP implementation and generates a homogeneous electric field along the *z*‐axis, within which the surface is relaxed both with and without adsorbates (${\mathrm{CO}}_{2}$ and CO). The corresponding electrode potential E can be related to the field strength $E_{z}$ (refer to the Supporting Information for details) [48].

The results obtained using the sawtooth approach were then extended by calculating GC adsorption energies with VASPsol. To obtain the GC energies, a constant charge ranging from 0 to −2.25 V Å^−1^ (−3.00 V Å^−1^ for chemisorbed ${\mathrm{CO}}_{2}$) was applied using implicit solvation as implemented in VASPsol [80, 81]. To ensure meaningful values for the electron chemical potential, a symmetric slab was constructed with adsorbate molecules placed on both sides. To relate these results to the experimental scale, we used the computational ferrocenium electrode as the reference electrode [50, 51]. More computational details can be found in the Supporting Information.

## Funding

This study was supported by the Helmholtz‐Gemeinschaft, Novo Nordisk Fonden (0085735) and Bundesministerium für ­Forschung, Technologie und Raumfahrt (03ZU1205UA).

## Conflicts of Interest

The authors declare no conflicts of interest.

## Supporting information

Supplementary Material

## Data Availability

The data that support the findings of this study are openly available in the KITopen repository. The experimental measurements and the results of the kinetic modeling can be accessed at https://doi.org/10.35097/shv7ushtuddj8xz6, while the DFT‐obtained data are available at https://doi.org/10.35097/55853f8gc1b5xbu8.
