# Supplementary material for: Mechanistic Insights Into Zn‐Promoted CO2 Electroreduction on α‐CuZn Alloys in Aprotic Media
Source: ChemSusChem. 2026 Oct 30;19(21):e70800. doi: 10.1002/cssc.70800 (PMC13722219; doi:10.1002/cssc.70800)
Supplement: Supplementary file 1 [file CSSC-19-e70800-s001.pdf]

Supporting Information

Mechanistic Insights into Zn-Promoted CO_2 Electroreduction on α-CuZn Alloys in Aprotic Media

Niklas Oppel,[a] Liana Savintseva,[b] Philipp Röse,[a] Georg Kastlunger,[c] Felix Studt,[b,d] Ulrike Krewer*[a]

[a] N. Oppel, Dr. P. Röse, Prof. Dr.-Ing. U. Krewer*
Karlsruhe Institute of Technology
Institute for Applied Materials – Electrochemical Technologies
Adenauerring 20b, 76131 Karlsruhe, Germany
E-mail: ulrike.krewer@kit.edu

[b] L. Savintseva, Prof. Dr. F. Studt
Karlsruhe Institute of Technology
Institute of Catalysis Research and Technology
Hermann-von-Helmholtz-Platz 1, 76344 Eggenstein-Leopoldshafen, Germany

[c] Dr. G. Kastlunger
Technical University of Denmark
Department of Physics
Fysikvej, 2800 Kongens Lyngby, Denmark

[d] Prof. Dr. F. Studt
Karlsruhe Institute of Technology
Institute for Chemical Technology and Polymer Chemistry
Engesserstr. 18/20, 76131 Karlsruhe, Germany

Contents

1. Material characterization via XRD **3**

2. Experimental Setup **4**

3. Reference Electrode Calibration **4**

4. Experimental Cyclic Voltammetry Datasets **5**

5. Experimental Linear Sweep Voltammetry Dataset **8**

6. Determination of Double Layer Capacitance **9**

7. DFT Calculation Details **10**
7.1. General Setup 10
7.1.1. Dispersion Contribution to the Adsorption Energies 12
7.1.2. Surface Coverage 12
7.2. Saw-Tooth Approach 13
7.2.1. Theory 13
7.2.2. Results 13
7.3. Grand-Canonical Calculations 13

8. Experimental and Simulated CVs at Different Scan Rates **16**
8.1. Model Validation 18
8.2. Kinetic Parameter 19

9. Steady-state Simulation on Roughened CuZn25 **20**

10. Parameter Sensitivity Analysis **21**
10.1. Case A 21
10.2. Case B 22

11. Simulation of Case A with Trend-consistent CuZn20 Parameters **23**

12. Impact of Electrolyte Decomposition on Kinetic Modeling **24**
12.1. Background Correction for Electrolyte Decomposition 24
12.2. Cyclic Voltammograms under Ar Atmosphere 26

1. Material characterization via XRD

X-ray diffraction (XRD) patterns of the α-CuZn, pure Cu, and pure Zn samples were collected using a Bruker D8 ADVANCE X-ray diffractometer operated at 40 kV and 40 mA. Cu Kα-radiation was used as the X-ray source. Data were collected in a 2θ range of 10° to 90° with a step size of 0.01° and a step time of 1.5 s in continuous scanning mode. DIFFRAC.EVA software was employed for baseline correction.

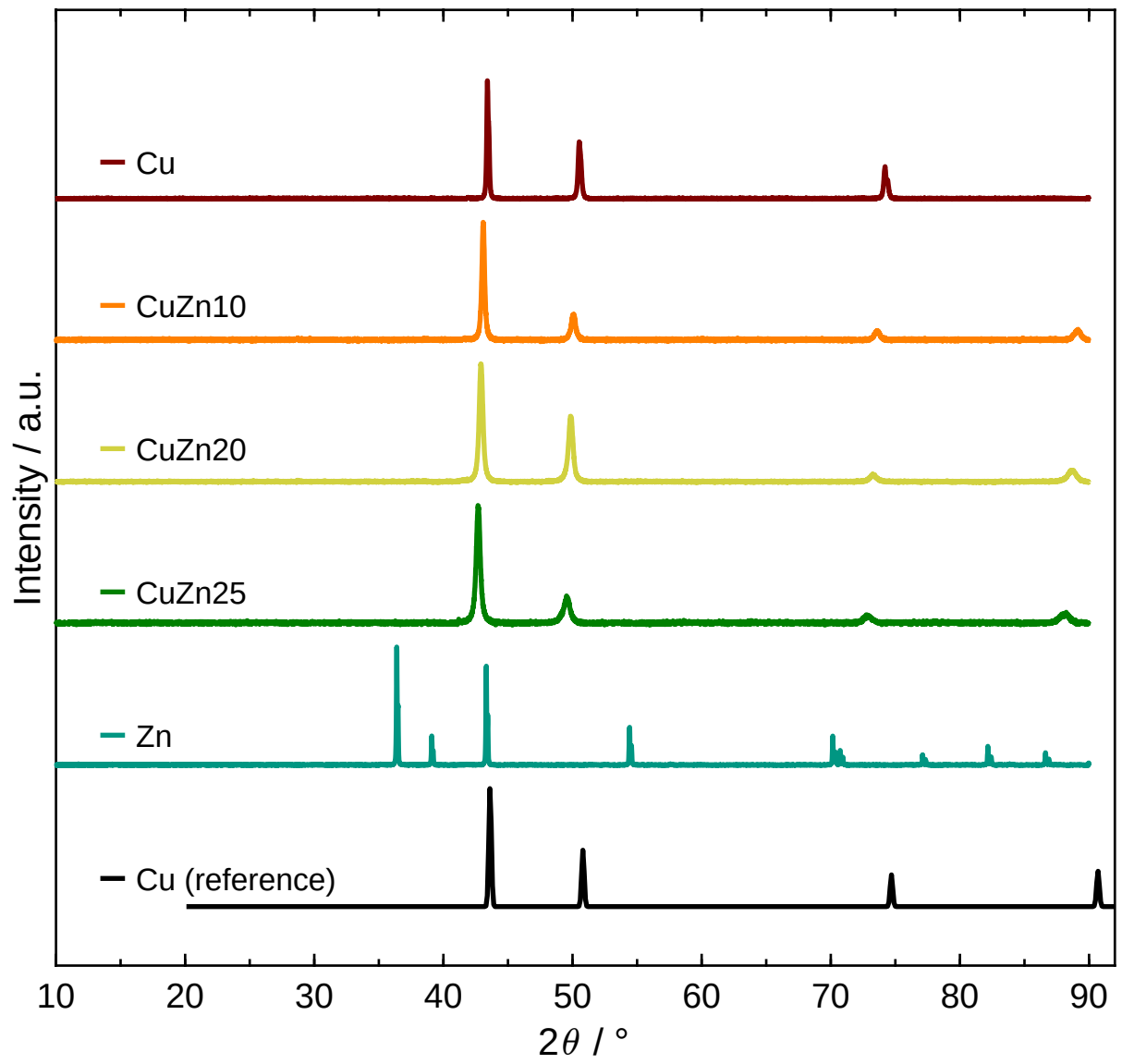

Figure S1. X-Ray diffractograms of Cu, CuZn10, CuZn20, CuZn25, and Zn electrodes. Bulk Cu shown as reference (ICSD no. 01-088-3992).

Increasing Zn content leads to a systematic shift in diffraction peaks toward lower Bragg angles, indicating lattice expansion due to the incorporation of larger Zn atoms into the Cu fcc lattice.[1]
A difference in relative peak intensities is observed across the compositions: CuZn10 and CuZn25 possess a highly suppressed (200) reflection, resulting in a different (111)-to-(200) peak ratio when compared to pure Cu and CuZn20. In terms of facet distribution, this indicates a crystallographic orientation predominantly composed of (111) facets on the CuZn10 and CuZn25 planar electrodes with a significantly reduced fraction of exposed (100) facets compared to the Cu and CuZn20 samples.

2. Experimental Setup

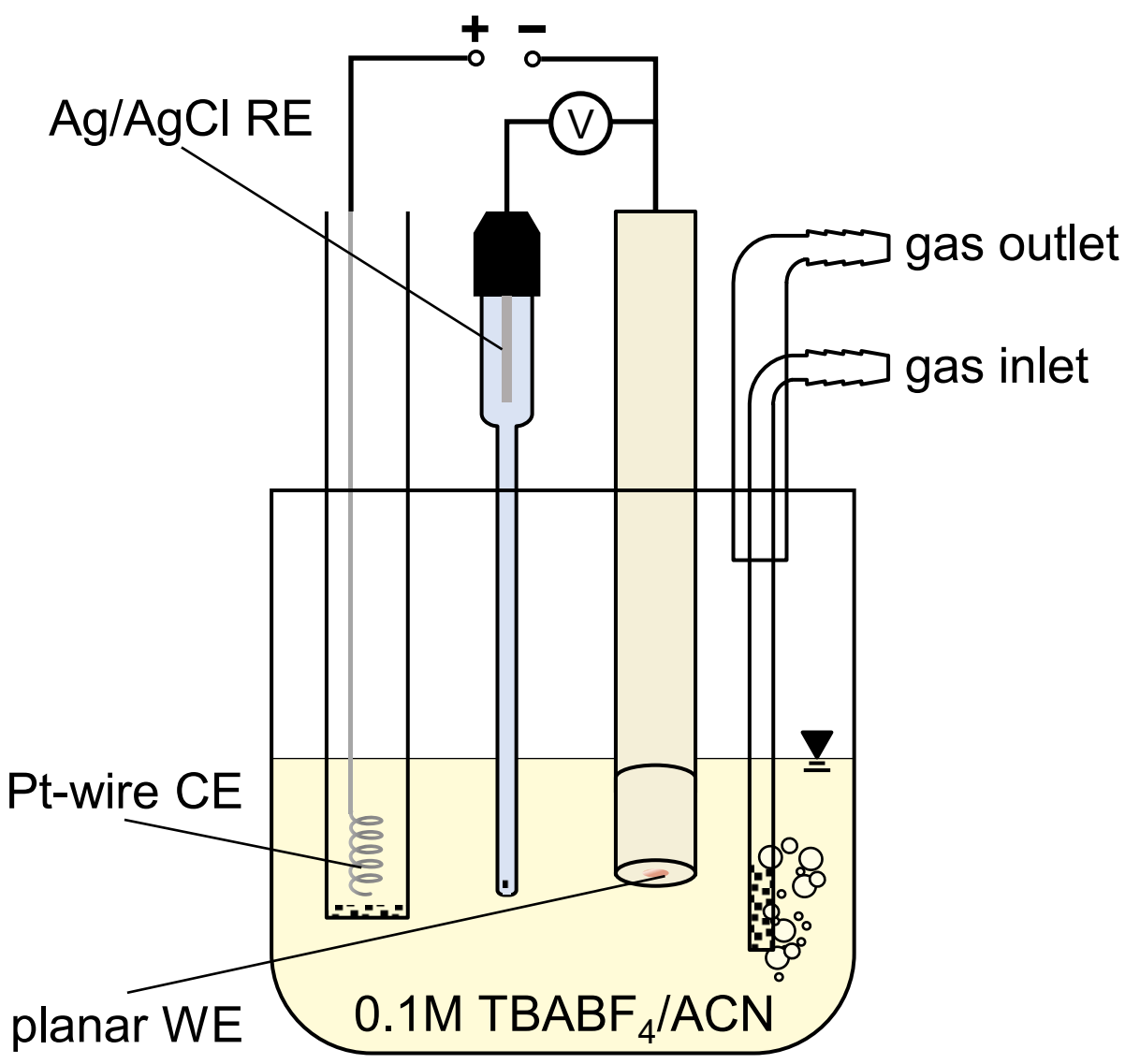

Figure S2. Schematic of the electrochemical setup used for CO_2 reduction measurements in 0.1 mol L^{-1} $TBABF_4$/acetonitrile. The configuration as detailed in the main manuscript consists of a planar working electrode (WE), Ag/AgCl reference electrode (RE), and Pt-wire counter electrode (CE), with gas inlet and outlet lines.

3. Reference Electrode Calibration

For potential conversion between the computational ferrocene electrode and the experimental Ag/AgCl reference electrode, the Ag/AgCl electrode was calibrated via cyclic voltammetry (CV) of a 1 mmol ferrocene containing 0.1 mol L^{-1} $TBABF_4$ acetonitrile solution. CV measurements were performed using a polished Pt disk working electrode, a coiled Pt wire counter electrode, and the Ag/AgCl reference electrode with a scan rate of 100 mV s^{-1}. A Pt working electrode was used since the redox potential of the ferrocene/ferrocenium (Fc^0/Fc^+) couple is more positive than the applicable potential window of the Cu and CuZn electrodes. The half-wave potential ($E_{1/2}$) of the Fc^0/Fc^+ redox couple was determined to be +0.515 V vs. Ag/AgCl.

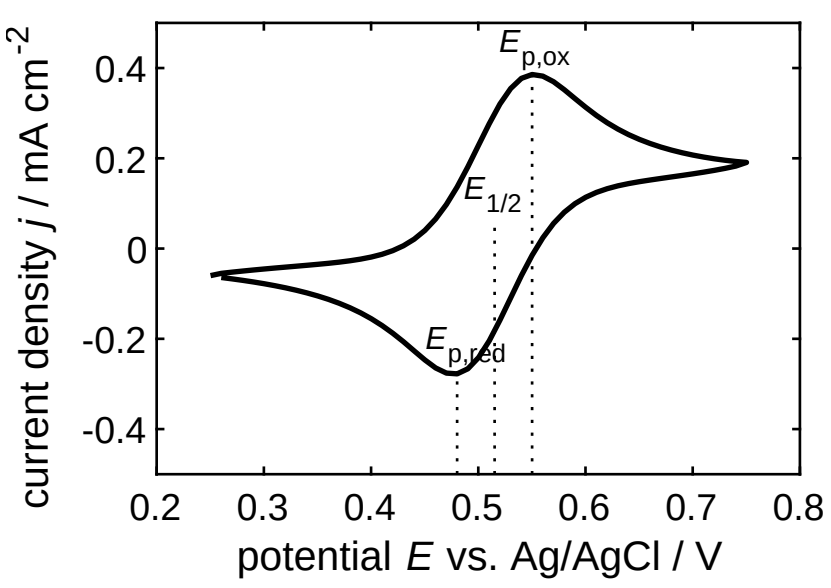

Figure S3. Cyclic voltammogram of 1 mmol ferrocene in 0.1 mol L^{-1} $TBABF_4$ acetonitrile solution at a Pt disk working electrode. Scan rate: 100 mV s^{-1}. The half-wave potential ($E_{1/2}$) was estimated to be +0.515 V vs. Ag/AgCl.

4. Experimental Cyclic Voltammetry Datasets

Cu

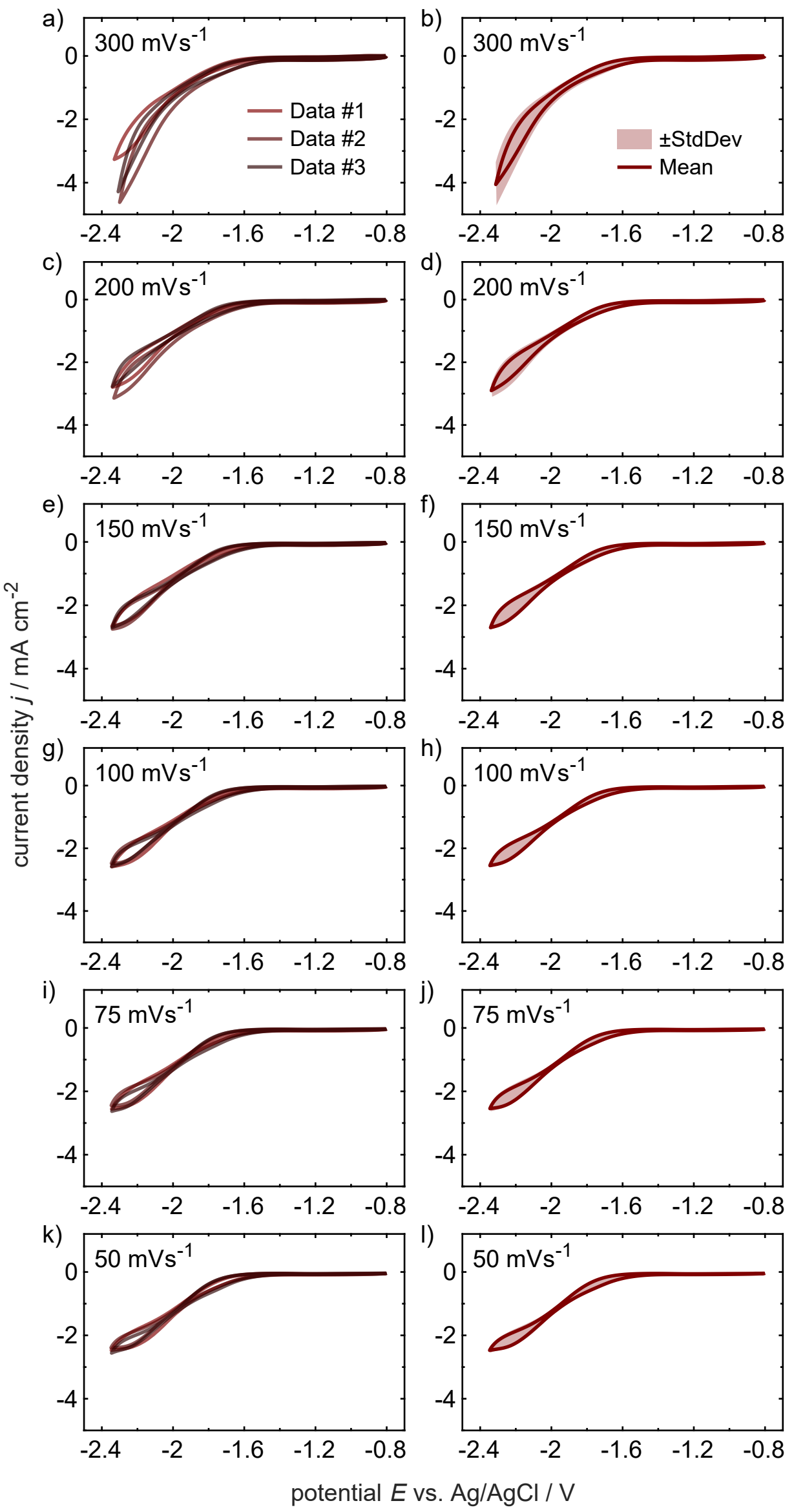

Figure S4. Experimental CVs of Cu in CO_2-saturated 0.1 mol L^{-1} $TBABF_4$/acetonitrile electrolyte at scan rates from 300 mV s^{-1} to 50 mV s^{-1}. Left panels show the individual CVs of the third cycle from three independent measurements. Right panels display the average CV from the third cycle and standard deviation. All potentials are *iR*-corrected.

CuZn10

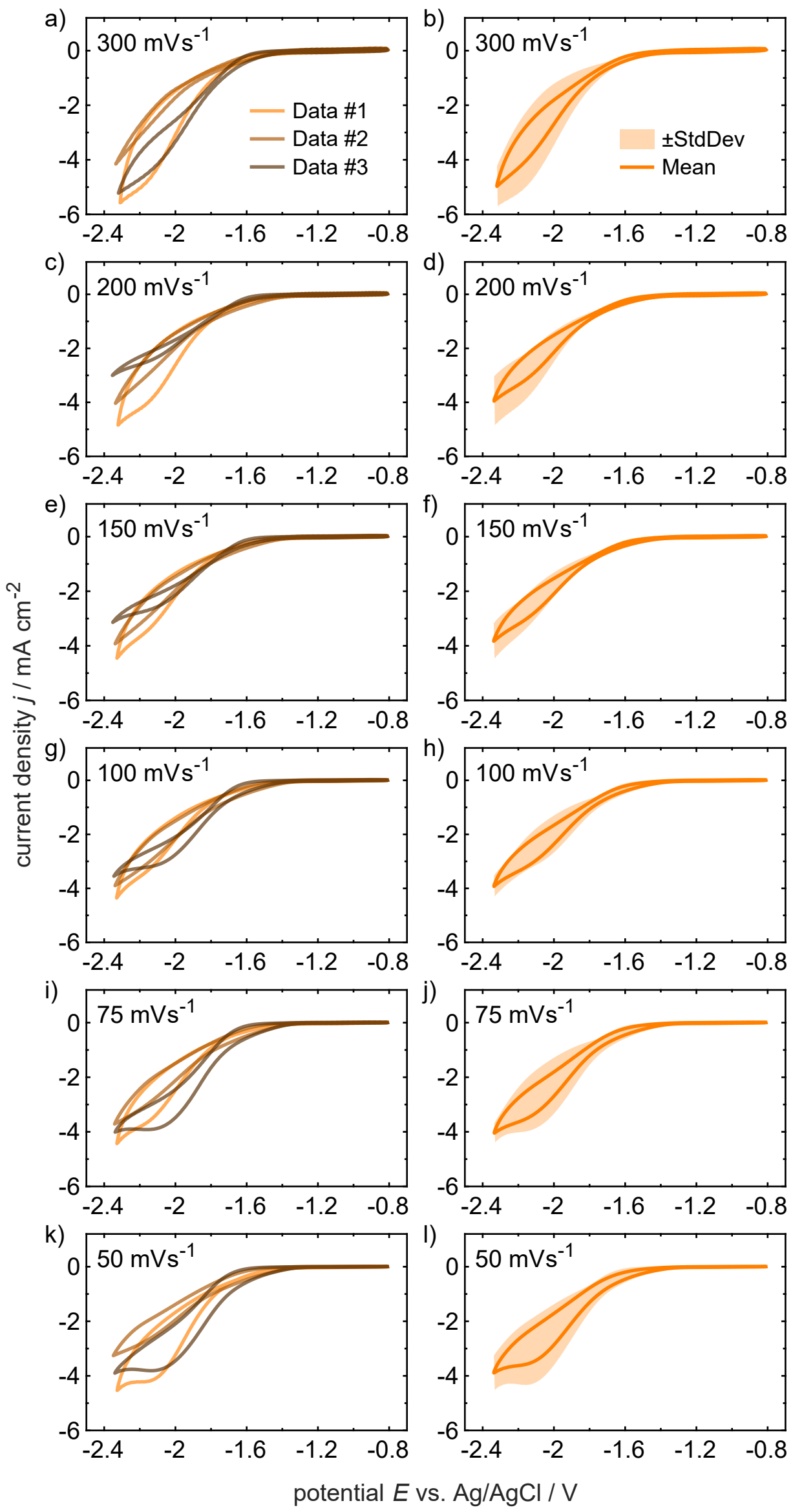

Figure S5. Experimental CVs of CuZn10 in CO_2-saturated 0.1 mol L^{-1} $TBABF_4$/acetonitrile electrolyte at scan rates from 300 mV s^{-1} to 50 mV s^{-1}. Left panels show the individual CVs of the third cycle from three independent measurements. Right panels display the average CV from the third cycle and standard deviation. All potentials are *iR*-corrected.

CuZn20

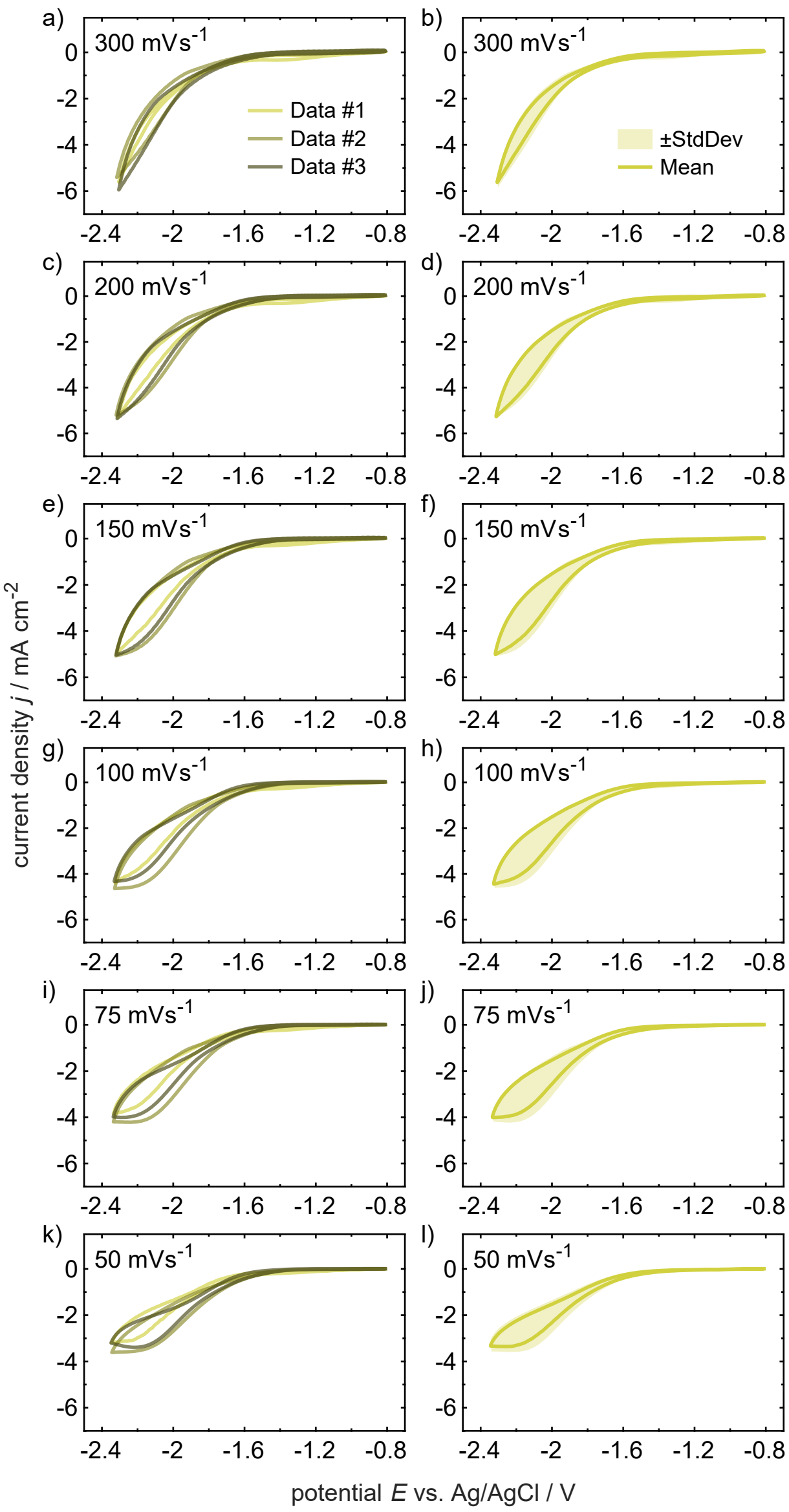

Figure S6. Experimental CVs of CuZn20 in CO_2-saturated 0.1 mol L^{-1} $TBABF_4$/acetonitrile electrolyte at scan rates from 300 mV s^{-1} to 50 mV s^{-1}. Left panels show the individual CVs of the third cycle from three independent measurements. Right panels display the average CV from the third cycle and standard deviation. All potentials are *iR*-corrected.

CuZn25

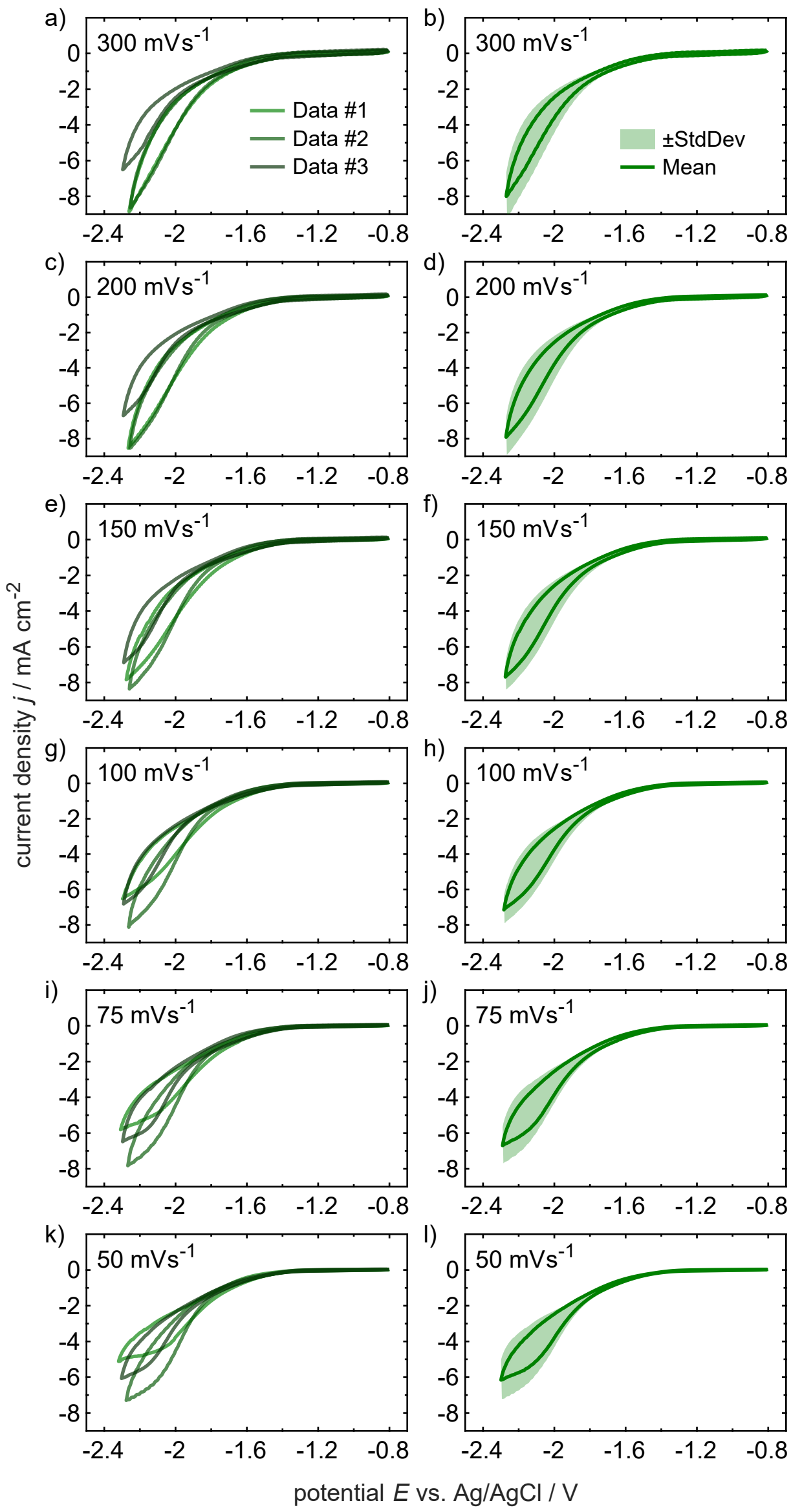

Figure S7. Experimental CVs of CuZn25 in CO_2-saturated 0.1 mol L^{-1} $TBABF_4$/acetonitrile electrolyte at scan rates from 300 mV s^{-1} to 50 mV s^{-1}. Left panels show the individual CVs of the third cycle from three independent measurements. Right panels display the average CV from the third cycle and standard deviation. All potentials are *iR*-corrected.

Zn

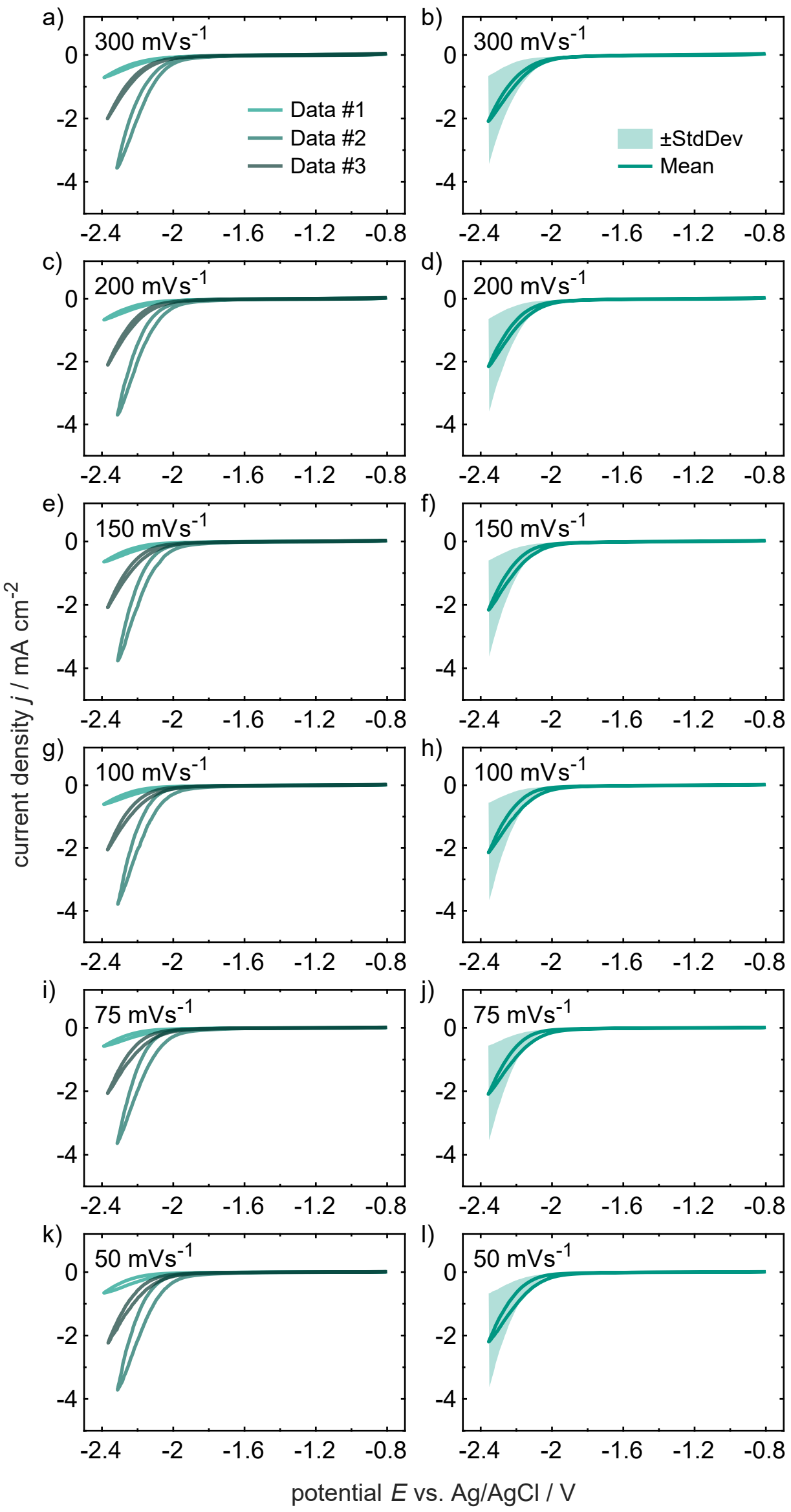

Figure S8. Experimental CVs of Zn in CO_2-saturated 0.1 mol L^{-1} $TBABF_4$/acetonitrile electrolyte at scan rates from 300 mV s^{-1} to 50 mV s^{-1}. Left panels show the individual CVs of the third cycle from three independent measurements. Right panels display the average CV from the third cycle and standard deviation. All potentials are *iR*-corrected.

5. Experimental Linear Sweep Voltammetry Dataset

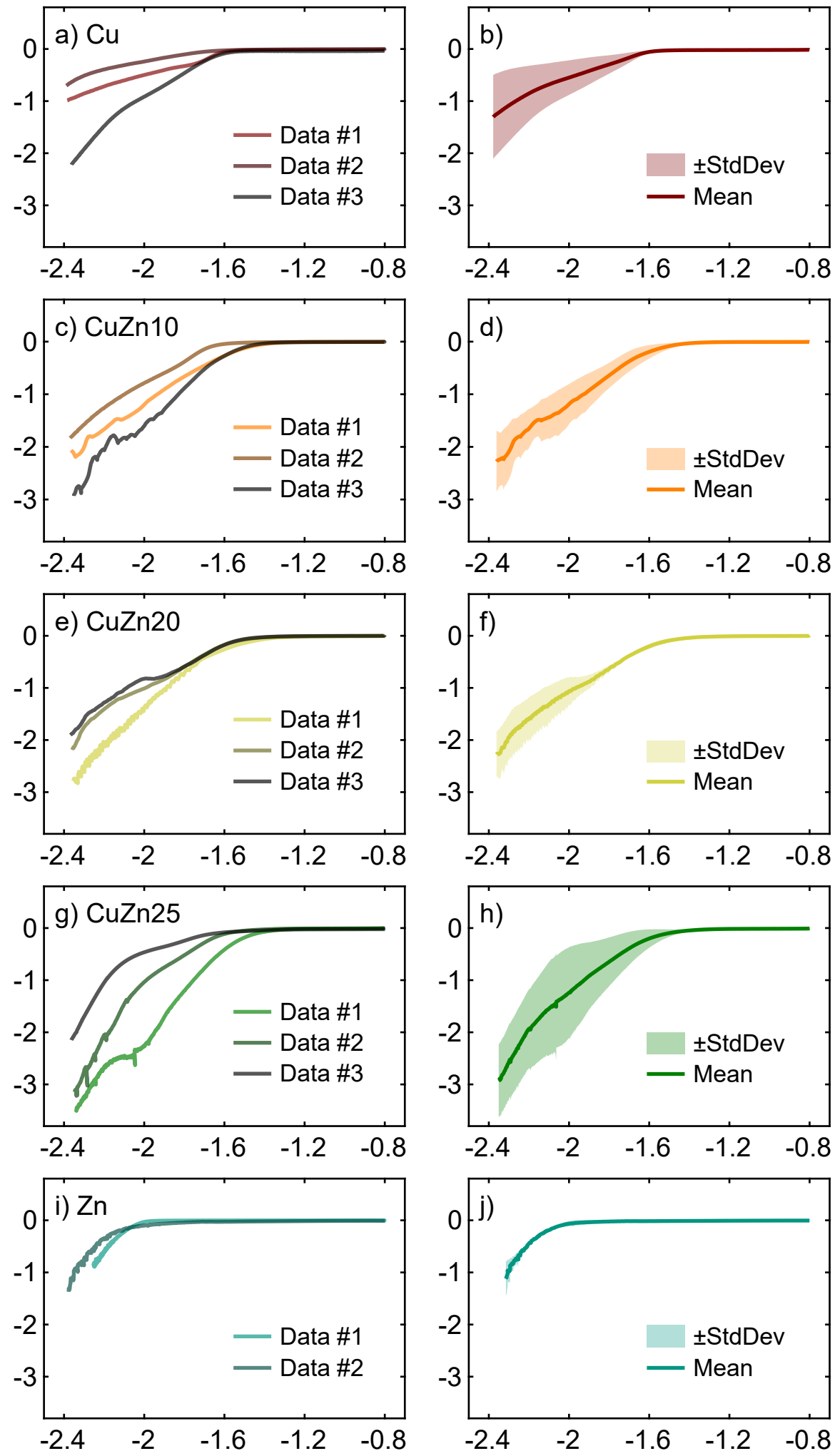

Figure S9. Experimental LSVs of Cu, CuZn10, CuZn20, CuZn25, and Zn in CO_2-saturated 0.1 mol L^{-1} $TBABF_4$/acetonitrile electrolyte at a scan rate of 1 mV s^{-1}. Left panels show the individual LVS from three independent measurements (two for Zn). Right panels display the average LSV and standard deviation. All potentials are *iR*-corrected.

6. Determination of Double Layer Capacitance

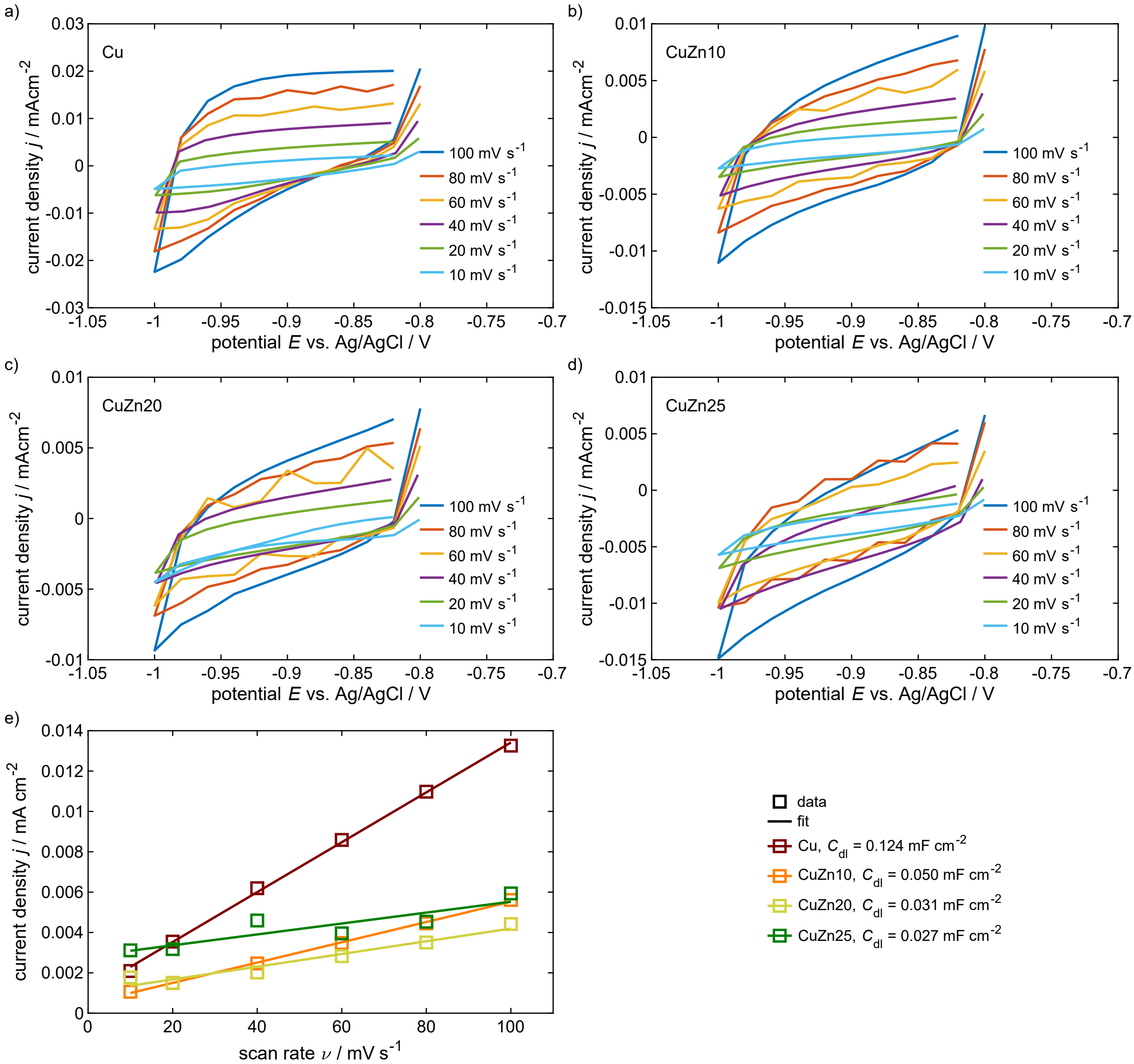

Figure S10. Cyclic voltammograms at varying scan rates (10 mV s^{-1} to 100 mV s^{-1}) in the double layer region for a) Cu, b) CuZn10, c) CuZn20, and d) CuZn25 electrodes vs. Ag/AgCl. Panel e) shows the linear fit of non-faradaic current density on scan rate used to determine the double layer capacitance C_{dl} (Cu: 0.124 mF cm^{-2}, CuZn10: 0.050 mF cm^{-2}, CuZn20: 0.031 mF cm^{-2}, CuZn25: 0.027 mF cm^{-2}). Measurements performed N_2-saturated in supporting electrolyte 0.1 mol L^{-1} $TBABF_4$ in acetonitrile to quantify capacitive currents independent of faradaic processes.

7. DFT Calculation Details

7.1. General Setup

Single unit cells of Cu and Cu_3Zn were optimized until force convergence below 0.02 eV Å^{-1}. Subsequently, a four-layer slab model representing the (111) surface was constructed using a 4×4 supercell, comprising 16 surface atoms, with 4 Zn surface atoms in the alloy configuration (Figure S11). A plane-wave basis set with a 450 eV energy cutoff and a 3×3×1 Γ-centered k-point sampling grid was used for Brillouin zone integration.

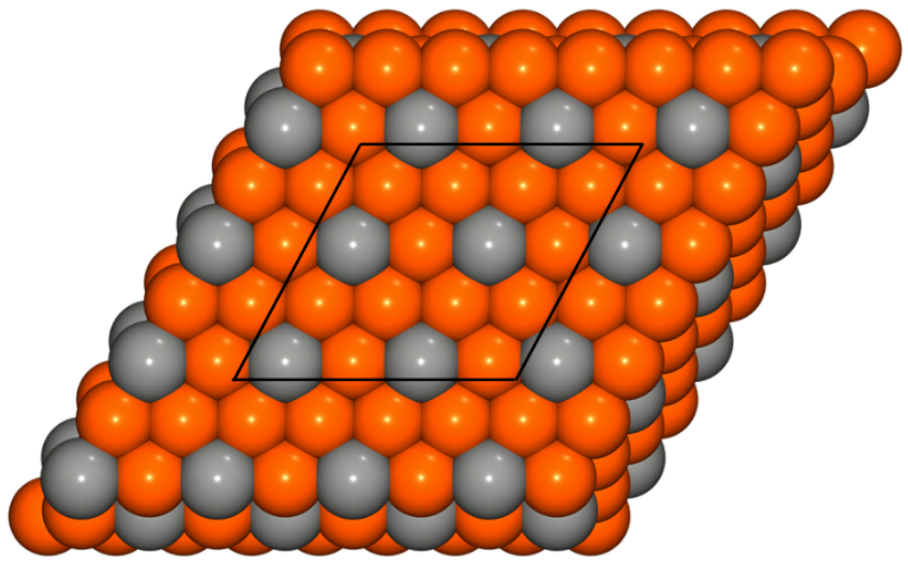

Figure S11. Top view on the Cu_3Zn surface.

k-point convergence was assessed using the adsorption energy of CO on Cu(111) as a test case. As shown in Figure S12, the adsorption energy is already well converged at the 3×3×1 mesh, and denser k-point grids lead to only negligible changes. To prevent spurious interactions, a 20 Å vacuum layer was included, perpendicular to the slab during geometry optimization.

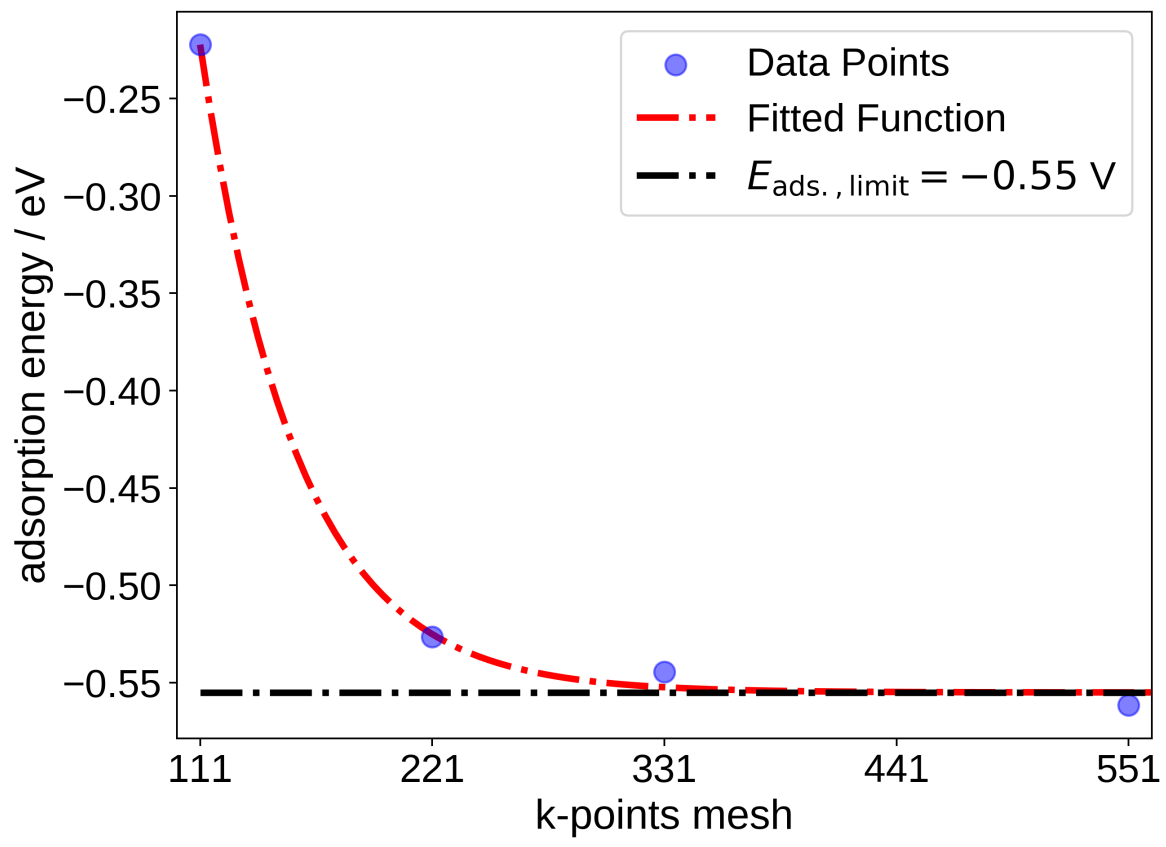

Figure S12. CO adsorption energy on Cu(111) surface as a function of k-points mesh. The structures were optimized at $3 \times 3 \times 1$ grid.

Zn segregation in the two uppermost layers was additionally examined by single-point calculations using a reduced 2×2 slab model. In contrast to the larger 4×4 slab, this truncated model only allows highly symmetric Zn arrangements. Nevertheless, it indicates that the ideal alloy configuration, in which Zn occupies one quarter of the surface sites, is among the most energetically favorable structures (Figure S13).
Adsorption energies E_{ads} of CO_2 and CO were calculated relative to the gas phase molecule in vacuum according to

$$E_{\text{ads}} = E_{\text{slab+CO}_2} - E_{\text{slab}} - E_{\text{CO}_2}, \tag{S1}$$

where $E_{\text{slab+CO}_2}$, E_{slab}, and E_{CO_2} are the potential-dependent energies of the CO_2-adsorbed system and the clean slab, and the gas-phase CO_2, respectively, with the latter being independent of the applied potential. This definition of the adsorption energy is valid for both potential-dependent energies obtained using the saw-tooth method and those obtained with the grand-canonical approach. Thermochemical properties were evaluated from vibrational frequency calculations within the harmonic approximation, as implemented in VASPkit, at a temperature of 298.15 K. The Gibbs free energies were then computed according to

$$\Delta G = \Delta E + \Delta ZPE - T\Delta S. \tag{S2}$$

The corresponding thermochemical corrections are summarized in Table S1, and the adsorption enthalpies are reported in Table S2.

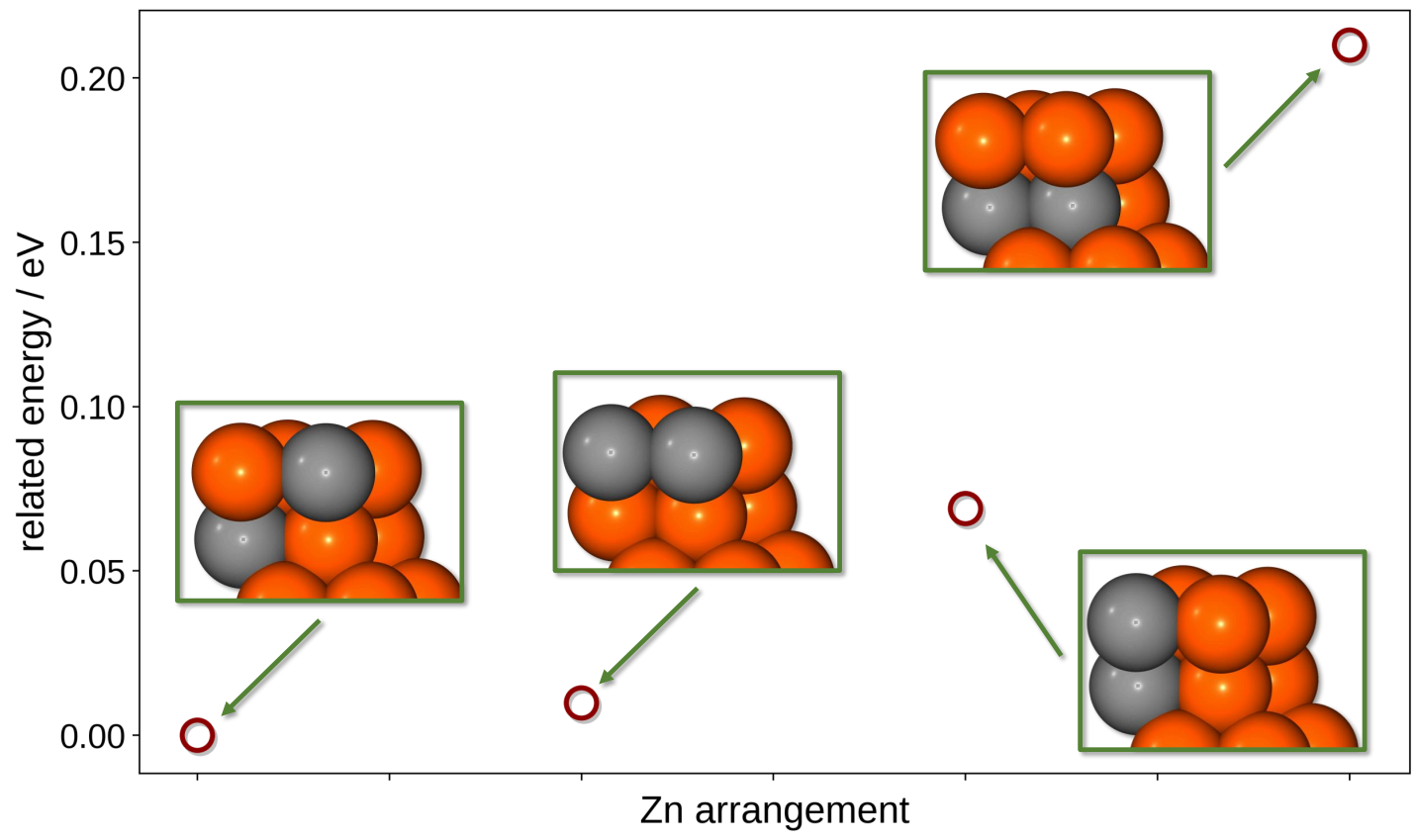

Figure S13. Relative energies of different configurations with two Zn atoms in a 2×2 Cu(111) slab representing the CuZn25 composition. Energies are given relative to the lowest-energy structure.

Table S1. Thermochemical corrections to the DFT energies for T = 298.15 K.

		CO_2		CO	
Facet	Site	ZPE / eV	TS / eV	ZPE / eV	TS / eV
Cu(111)	Cu	0.31	0.26	0.18	0.19
$Cu_3Zn(111)$	Cu	0.31	0.26	0.18	0.06
	Zn	0.31	0.20	0.16	0.09
Vacuum	-	0.31	0.66	0.13	0.61

Table S2. DFT calculated adsorption enthalpies ΔH_{ads} of CO and CO_2 on Cu(111) and $Cu_3Zn(111)$ at the on top site.

		CO_2		CO	
Facet	Site	ΔH_{ads} / eV	d / Å	ΔH_{ads} / eV	d / Å
Cu(111)	Cu	–0.21	3.60	–0.50	1.87
$Cu_3Zn(111)$	Cu	–0.16	3.70	–0.44	1.87
	Zn	–0.13	3.56	+0.10	2.09

7.1.1. Dispersion Contribution to the Adsorption Energies

Adsorbed states can be approximately classified as chemisorbed or physisorbed based on their adsorption enthalpies and adsorbate–metal distances. However, such a distinction is necessarily somewhat arbitrary, since there is no sharp boundary between these two limiting cases. To gain additional insight into the nature of adsorption, the dispersion contribution to the adsorption energies can be analyzed. Since BEEF-vdW functional does not provide an explicit decomposition of the dispersion contribution, additional calculations were therefore performed using the PBE-D3 approach. The corresponding results are summarized in Table S3. It can be seen that the dispersion contribution to CO adsorption on Cu atoms on both surfaces is rather small, whereas CO_2 adsorption and CO adsorption on Zn sites are dominated by dispersion. Although we are aware that the total adsorption energies predicted by PBE-D3 (and PBE in general) are not sufficiently accurate, this analysis is still useful on a qualitative level. These conclusions are consistent with the analysis based on adsorbate–metal distances and adsorption enthalpies.

Table S3. Adsorption enthalpies obtained with PBE-D3 and the corresponding dispersion contributions for CO_2 and CO on Cu(111) and $Cu_3Zn(111)$.

		CO_2		CO	
Facet	Site	ΔH_{ads} / eV	Dispersion / eV	ΔH_{ads} / eV	Dispersion / eV
Cu(111)	Cu	–0.25	–0.31	–0.96	–0.39
$Cu_3Zn(111)$	Cu	–0.28	–0.25	–1.08	–0.32
	Zn	–0.21	–0.29	–0.42	–0.31

7.1.2. Surface Coverage

Let us now consider the surface CO surface coverage based on the results above. Assuming that the surface population of the adsorbed state follows a Boltzmann distribution, the relative change in coverage upon changing the adsorption energy can be estimated as

$$\frac{\theta_2}{\theta_1} \approx \exp\left(-\frac{\Delta E_{ads}}{k_B T}\right), \tag{S3}$$

where θ_1 and θ_2 are the coverages corresponding to adsorption energies $E_{ads,1}$ and $E_{ads,2}$, respectively, and $\Delta E_{ads} = E_{ads,2} - E_{ads,1}$. For the present case, the adsorption energy of CO changes from -0.02 for Cu(111) to 0.16 eV for Cu_3Zn, corresponding to $\Delta E_{ads} = 0.18$ eV. At room temperature, this gives

$$\frac{\theta_2}{\theta_1} \approx \exp\left(-\frac{0.18}{k_B T}\right) \approx 9 \times 10^{-4}, \tag{S4}$$

indicating that the surface coverage of CO is expected to decrease by approximately three orders of magnitude. Thus, even a modest increase in adsorption energy by 0.18 eV can strongly reduce the equilibrium population of the adsorbed state. Moreover, as discussed above, we assume that at least one quarter of the surface atoms on the Cu_3Zn surface are Zn atoms. Since adsorption on Zn atoms is highly unfavorable (Table S2), this would make the difference in coverages even more pronounced.

7.2. Saw-Tooth Approach

7.2.1. Theory

These vacuum adsorption energies neglect electrode potential and solvent effects; thus, they provide only a simplified picture of the electrochemical interface. The subsequent analysis, therefore, focuses on the potential-dependent adsorption energy curves obtained using the saw-tooth potential method. The saw-tooth calculations were performed in vacuum, assuming that the electric double-layer capacitance is independent of the presence of adsorbates.
The applied sawtooth potential, ranging from +0.25 V Å^{-1} to −1.50 V Å^{-1}, follows the VASP implementation and generates a homogeneous electric field along the z-axis, within which the surface is relaxed both with and without adsorbates (CO_2 and CO). The corresponding electrode potential E can be related to the field strength E_z through[2]

$$E_z = \frac{C(E - E_{\text{pzc}})}{\epsilon_0 \epsilon_r} \tag{S5}$$

Here, C denotes the interfacial capacitance, which can be calculated using VASPsol as described by Gauthier et al.,[3] based on the charge density σ and the work function Φ (Figure S15):

$$\sigma = C(\Phi - \Phi_0) \tag{S6}$$

$E - E_{\text{pzc}}$ represents the electrode potential relative to the potential of zero charge. To compare with experimental electrode potentials, we use the open-circuit potential (OCP) values since the experimental determination of E_{pzc} is not possible in acetonitrile due to the properties of the solvent. The respective values are $E_{\text{OCP,Cu}} = -0.38$ V and $E_{\text{OCP,Cu}_3\text{Zn}} = -0.35$ V vs. Ag/AgCl. The relative permittivity of acetonitrile is $\epsilon_r = 37.5$, and the permittivity of the interfacial solvent was taken as $\epsilon_r = 1.5$.

7.2.2. Results

It can be seen that CO_2 is poorly adsorbed (physisorbed) over the entire potential range on both investigated model surfaces, while CO, on the contrary, shows very different adsorption modes on Cu and Zn atoms (Figure S14). Moreover, Zn exhibits a strong affinity toward oxygen, introducing an additional possible adsorption configuration via the oxygen atom of CO_2 or CO. The adsorption configuration of CO_2 via the carbon atom shows little dependence on the potential. In contrast, for the CO_2(O)@Zn configuration, the distance between the adsorbate and the surface decreases significantly with increasing potential (Figure S14d), which could enhance the electron transfer process and, as a result, the CO_2 reduction rate.

The dipole moments and polarizabilities of the adsorbates can be estimated from a polynomial decomposition of the adsorption energy as a function of the external electric field (Eq. S7). It can be seen from the polarizability values (Table S4) that on Cu sites (both Cu(111) and Cu_3Zn(111)), the adsorption energy shows a weaker dependence on the external field (low polarizability values). In contrast, on Zn sites, the adsorption energies of CO and CO_2(O) are strongly affected by the applied potential. Thus, the difference in reactivity between Cu and Zn atoms becomes evident.

$$G_{\text{ads}}(E_z) = G_{\text{ads}}^{\text{pzc}} + \bar{p}\, E_z - \frac{\alpha}{2} E_z^2 \tag{S7}$$

Table S4. Dipole moment p_z and polarizability α_{zz} components along the surface normal, extracted from the adsorption energy curves as functions of the applied electric field E_z.

Adsorbate	Surface	Site	p_z [e Å]	α_{zz} [e Å V^{-1}]
CO	Cu(111)	Cu	-0.011	0.185
	Cu_3Zn(111)	Cu	-0.030	0.163
		Zn	0.021	0.280
CO_2	Cu(111)	Cu	0.002	0.181
	Cu_3Zn(111)	Cu	0.001	0.167
		Zn–C	0.008	0.152
		Zn–O	0.025	0.263

7.3. Grand-Canonical Calculations

To obtain the grand-canonical energies, a constant charge ranging from 0 to -2.25 (up to -3.50 for chemisorbed CO_2) was applied using implicit solvation as implemented in VASPsol. To ensure meaningful values for the electron chemical potential, a symmetric slab was constructed with adsorbate molecules placed on both sides. The slope of the linear relationship between

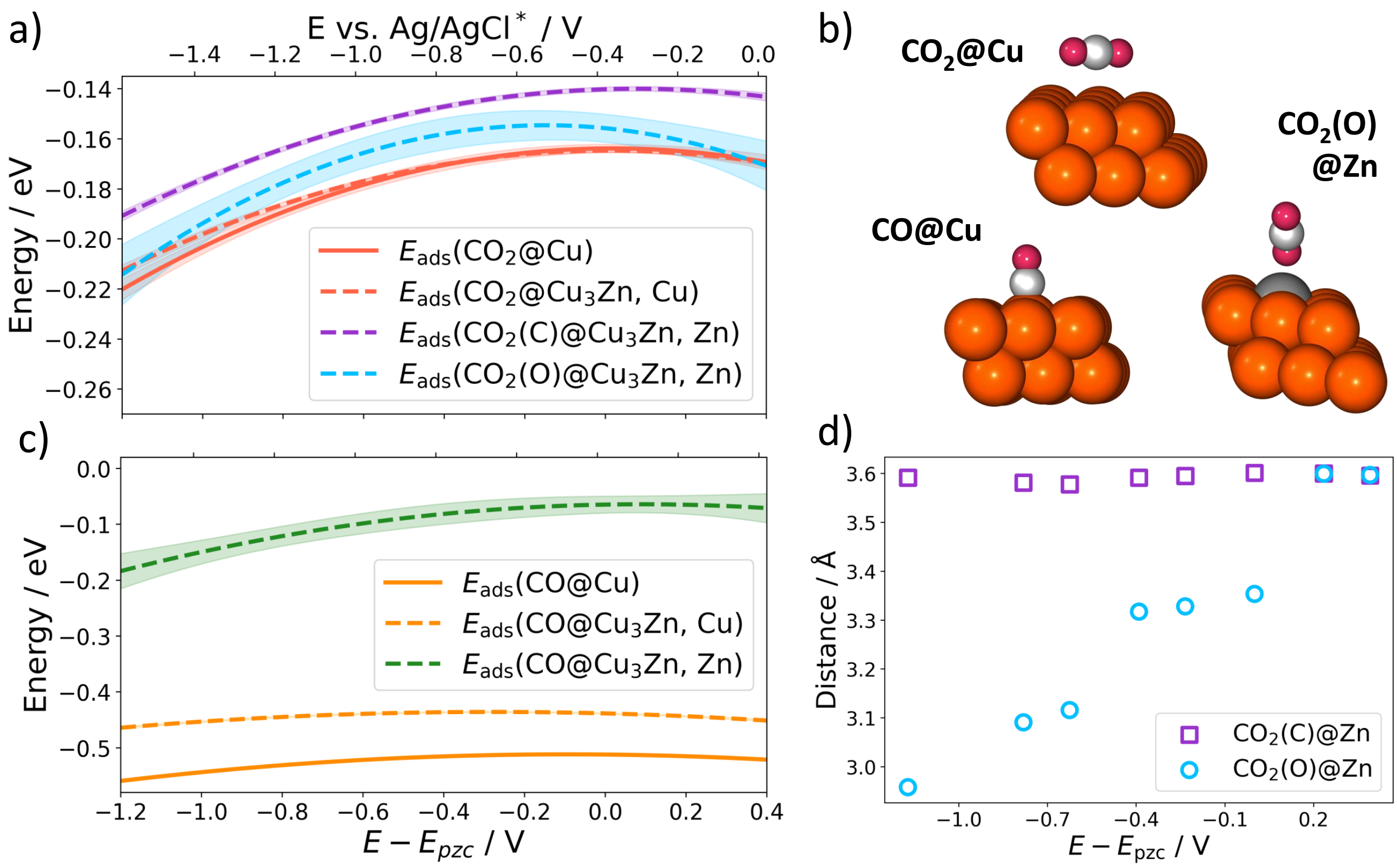

Figure S14. Saw-tooth potential results. a,c) Potential-dependent adsorption energies of CO_2 and CO, respectively at 0 K, with 2σ confidence intervals shown as shaded regions. The upper x-axis indicates the potential values versus Ag/AgCl, derived from the experimentally determined value of the open circuit potential. b) Optimized structures of the adsorbates on Cu(111) and Cu_3Zn surfaces at −1 V vs. E_{pzc} (equivalent to E_z = −0.75 V Å^{-1}); the slab size was reduced for clarity. d) The smallest distance between CO_2 molecule and the surface atoms as a function of the applied potential.

the surface charge density σ and the chemical potential μ (or work function Φ), evaluated at the value corresponding to the neutral system (Φ_0), yields the interfacial capacitance (Figure S15).
Calculations with different excess electrons yield canonical energies that are not directly comparable because the number of particles (electrons) differs between the systems. The canonical DFT energy can be straightforwardly converted into the grand-canonical potential Ω using Eq. S8.

$$\Omega = E_{\mathrm{DFT}} - N_e\mu \tag{S8}$$

The chemical potential μ is obtained as the sum of the Fermi level and the Fermi shift reported by VASPsol, following $\mu = \epsilon_F$ − FERMI_SHIFT.[3] And the canonical DFT energy in VASPsol can be obtained based on the SCF calculation energy as $E_{\mathrm{DFT}} = E_{\mathrm{SCF}} + N_e \cdot \mathrm{FERMI\ SHIFT}$. The interface capacitance was determined using the Debye length $\lambda_D = 6.7$, corresponding to a bulk ion concentration of 0.1 mol L^{-1}.

The electron chemical potential μ was converted to the electrode potential using the computational ferrocenium electrode (CFE) model via the free energy of the reaction Fc − 1 e$^-$ $\longrightarrow$ Fc$^+$, which defines the absolute potential of the CFE. In acetonitrile, this value was found to be 5.02 eV. This result agrees well with the experimental value of 5.05 eV and with our previous estimate of 4.95 eV obtained using the B3LYP hybrid functional in ORCA. Thus, the electrode potential is defined by

$$U_{\mathrm{CFE}} = -\mu - U_{\mathrm{ref}} = -\mu - 5.02 \tag{S9}$$

The determined potentials were then converted to the Ag/AgCl reference electrode by adding +0.515 V (cf. section 3: Reference Electrode Calibration). The resulting total energy and adsorption energy curves were fitted to the parabolic function as $ax^2 + bx + c$, where the coefficient a determines the capacitance. The parameters for the total energies of the systems are given in Table S5, and those for the adsorption energies in Table S6. Table S6 shows that the curvature a for CO@Cu is an order of magnitude higher than that for CO@Cu_3Zn, supporting the conclusion that the electric double-layer capacitance of the Cu electrode is more strongly affected by CO adsorption than Cu_3Zn.

Thermochemical corrections for the adsorbates were defined at each charge step and averaged. The resulting values in eV are 0.10 ± 0.04 for one-bond CO_2 Ch., 0.09 ± 0.01 for two-bond CO_2 on both surfaces, 0.11 ± 0.05 and 0.18 ± 0.03 for CO_2 Ph., and finally 0.09 ± 0.002 and 0.04 ± 0.002 for CO on Cu and Cu_3Zn surfaces, respectively. The obtained values confirm the common assumption that the thermochemical correction for adsorbates does not depend on the applied potential.

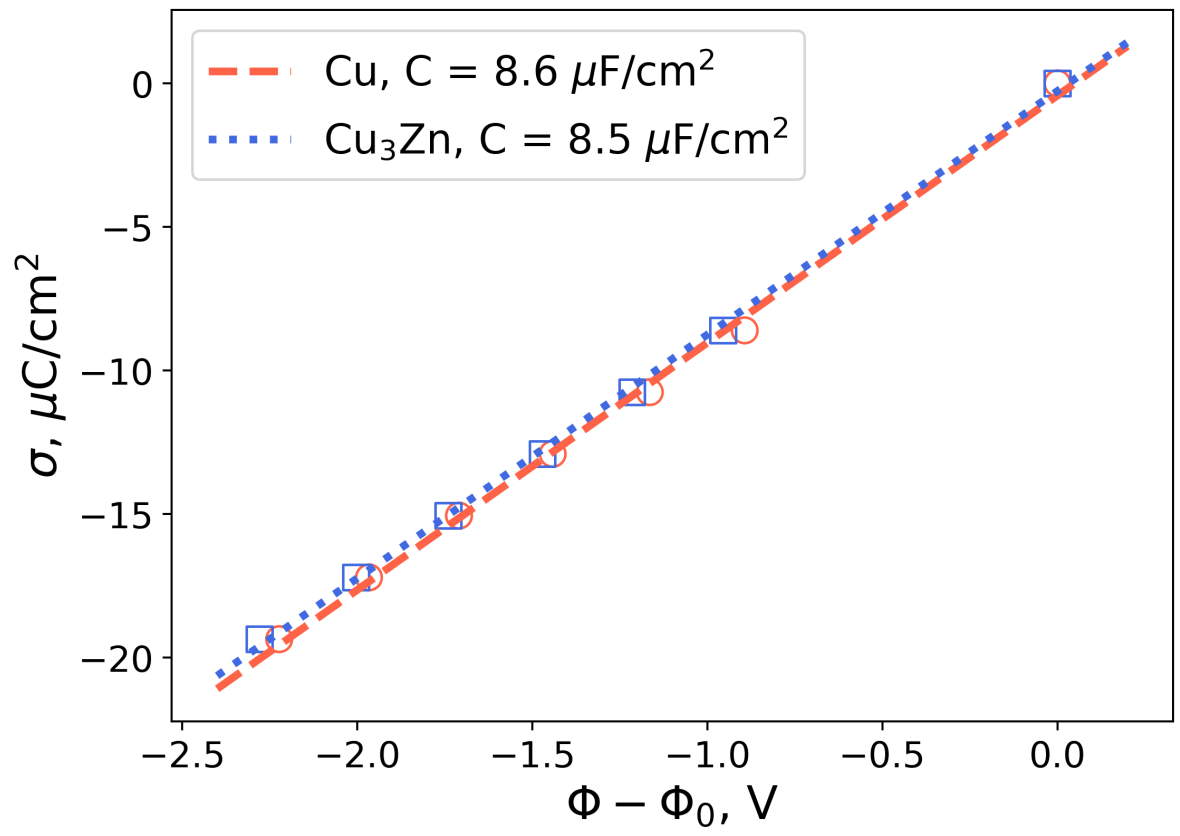

Figure S15. Theoretical charging curve of Cu and Cu3Zn (111) used to fit capacitance via $\sigma = C(\Phi - \Phi_0)$.

Table S5. Fitting parameters for the parabolic function of the total-energy curves, with energies given in eV and the electrode potential relative to the CFE given in V.

	a	b	c
Cu (111)	-0.515	-0.307	-63.961
CO@Cu	-0.548	-0.525	-51.504
CO_2@Cu, Ph.	-0.446	-0.140	-63.412
CO_2@Cu, *O-*CO	-0.529	0.282	-61.541
CO_2@Cu, *CO_2	-0.608	-0.107	-61.918
Cu_3Zn (111)	-0.504	-0.244	-26.485
CO@Cu_3Zn	-0.527	-0.351	-13.833
CO_2@Cu_3Zn, Ph.	-0.453	-0.138	-25.853
CO_2@Cu_3Zn, *O-*CO	-0.511	0.306	-24.153
CO_2@Cu_3Zn, *CO_2	-0.833	-1.353	-25.788

Table S6. Fitting parameters for the parabolic function of the adsorption-energy curves, with energies given in eV and the electrode potential relative to the CFE given in V.

	a	b	c
CO@Cu	-0.046	0.100	1.021
CO_2@Cu, Ph.	0.034	0.084	0.275
CO_2@Cu, *O-*CO	-0.007	0.294	1.210
CO_2@Cu, *CO_2	-0.046	0.100	1.021
CO@Cu_3Zn	-0.011	-0.053	0.109
CO_2@Cu_3Zn, Ph.	0.026	0.053	-5.901
CO_2@Cu_3Zn, *O-*CO	-0.003	0.274	-5.051
CO_2@Cu_3Zn, *CO_2	-0.165	-0.555	-5.869

8. Experimental and Simulated CVs at Different Scan Rates

Cu

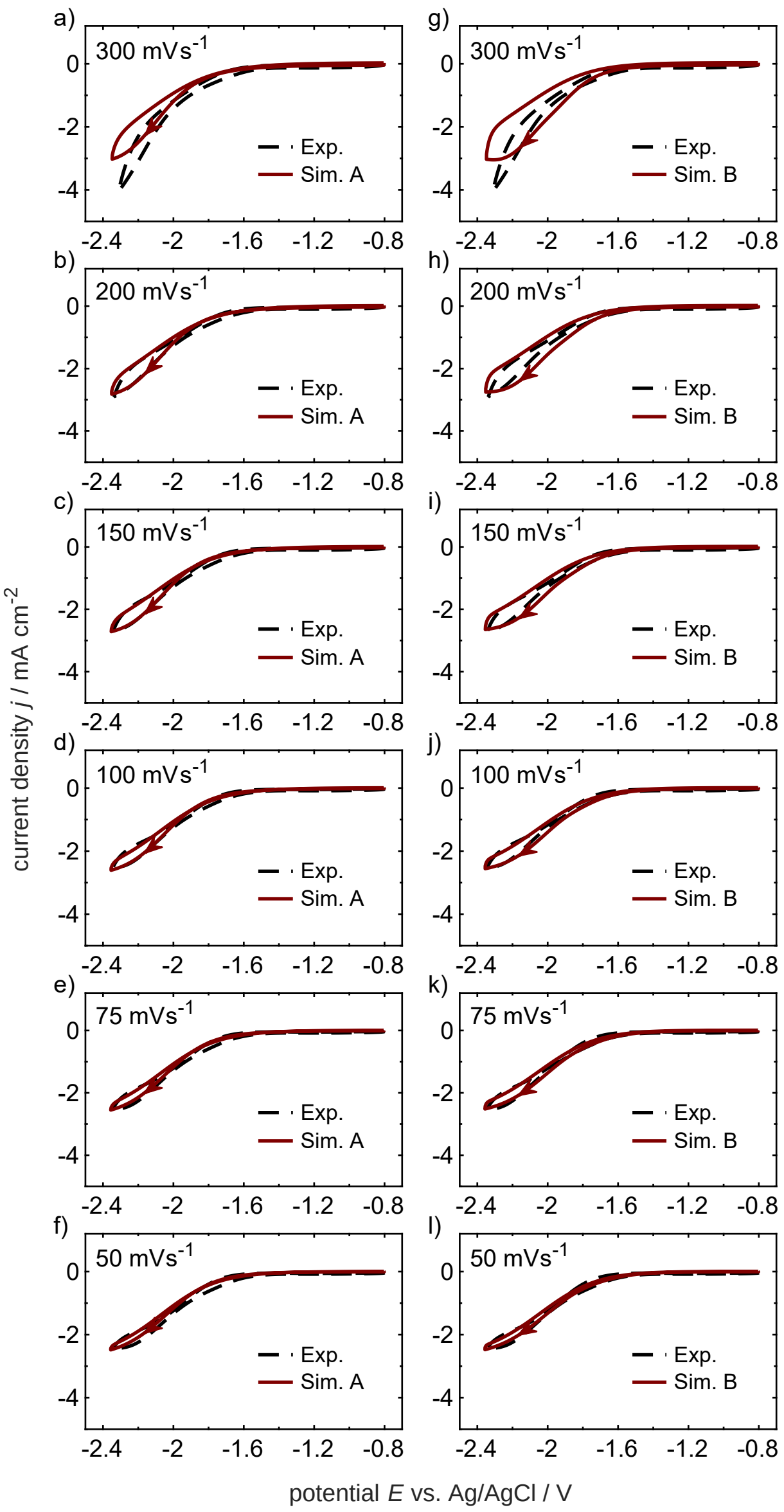

Figure S16. Comparison of simulated (solid line) and experimental (dashed line) CVs for a planar Cu electrode in CO_2-saturated 0.1 mol L^{-1} $TBABF_4$ acetonitrile electrolyte. CVs are presented for scan rates from 300 mV s^{-1} to 50 mV s^{-1} for Case A (a to f) and Case B (g to l). All CVs are *iR*-corrected and represent the third cycle of each measurement.

CuZn10

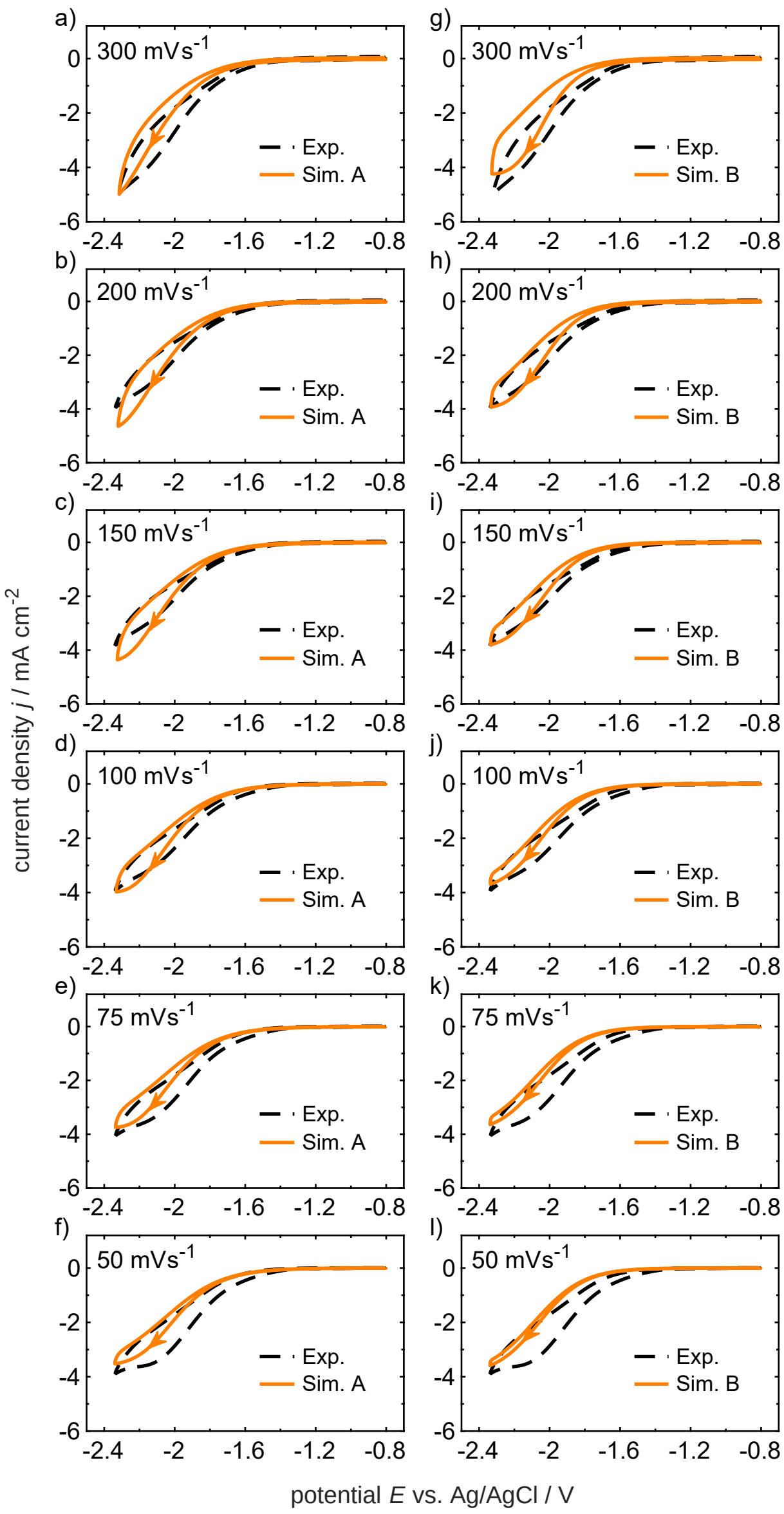

Figure S17. Comparison of simulated (solid line) and experimental (dashed line) CVs for a planar CuZn10 electrode in CO_2-saturated 0.1 mol L^{-1} $TBABF_4$ acetonitrile electrolyte. CVs are presented for scan rates from 300 mV s^{-1} to 50 mV s^{-1} for Case A (a to f) and Case B (g to l). All CVs are *iR*-corrected and represent the third cycle of each measurement.

CuZn20

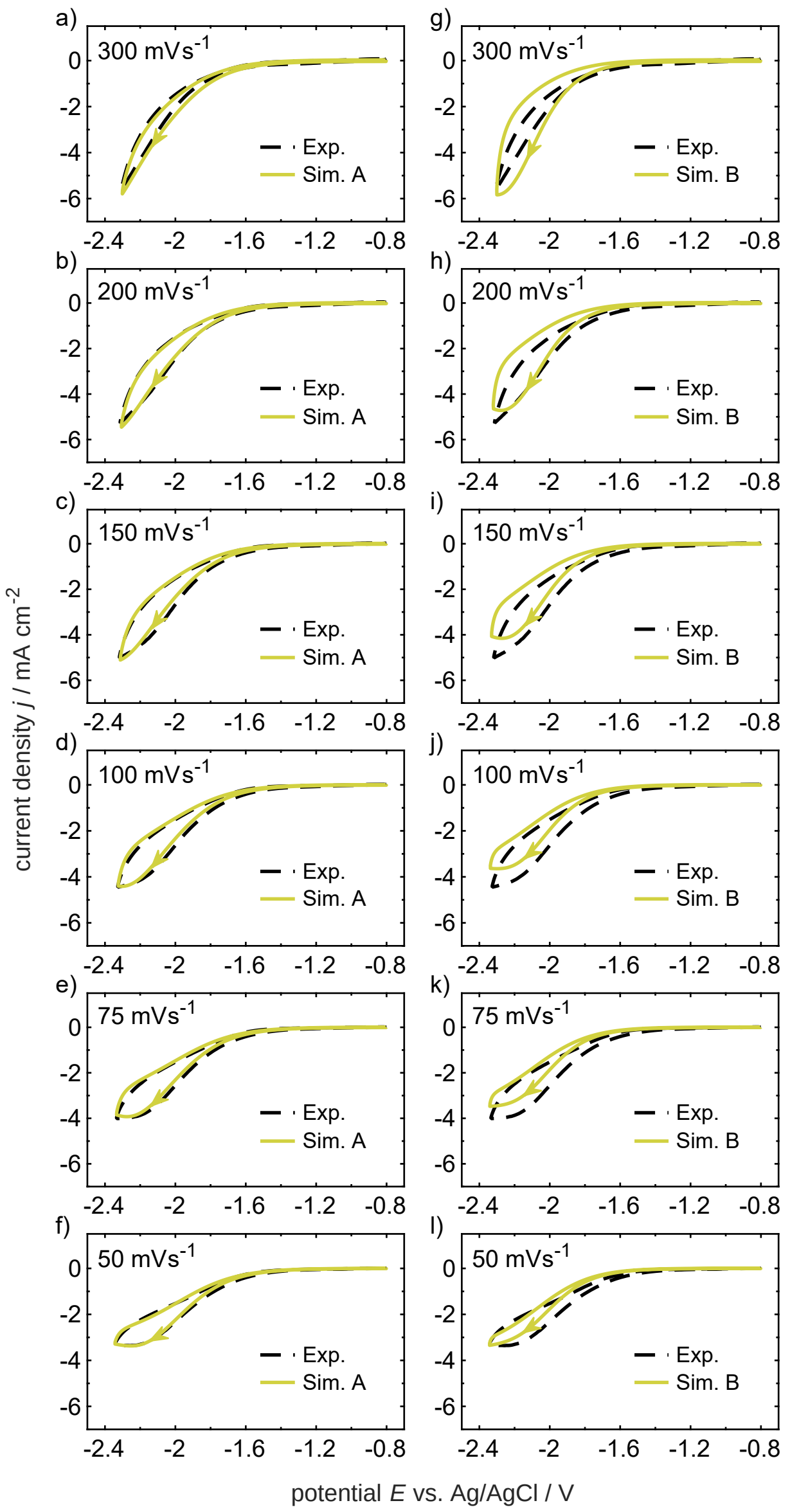

Figure S18. Comparison of simulated (solid line) and experimental (dashed line) CVs for a planar CuZn20 electrode in CO_2-saturated 0.1 mol L^{-1} TBABF$_4$ acetonitrile electrolyte. CVs are presented for scan rates from 300 mV s^{-1} to 50 mV s^{-1} for Case A (a to f) and Case B (g to l). All CVs are *iR*-corrected and represent the third cycle of each measurement.

CuZn25

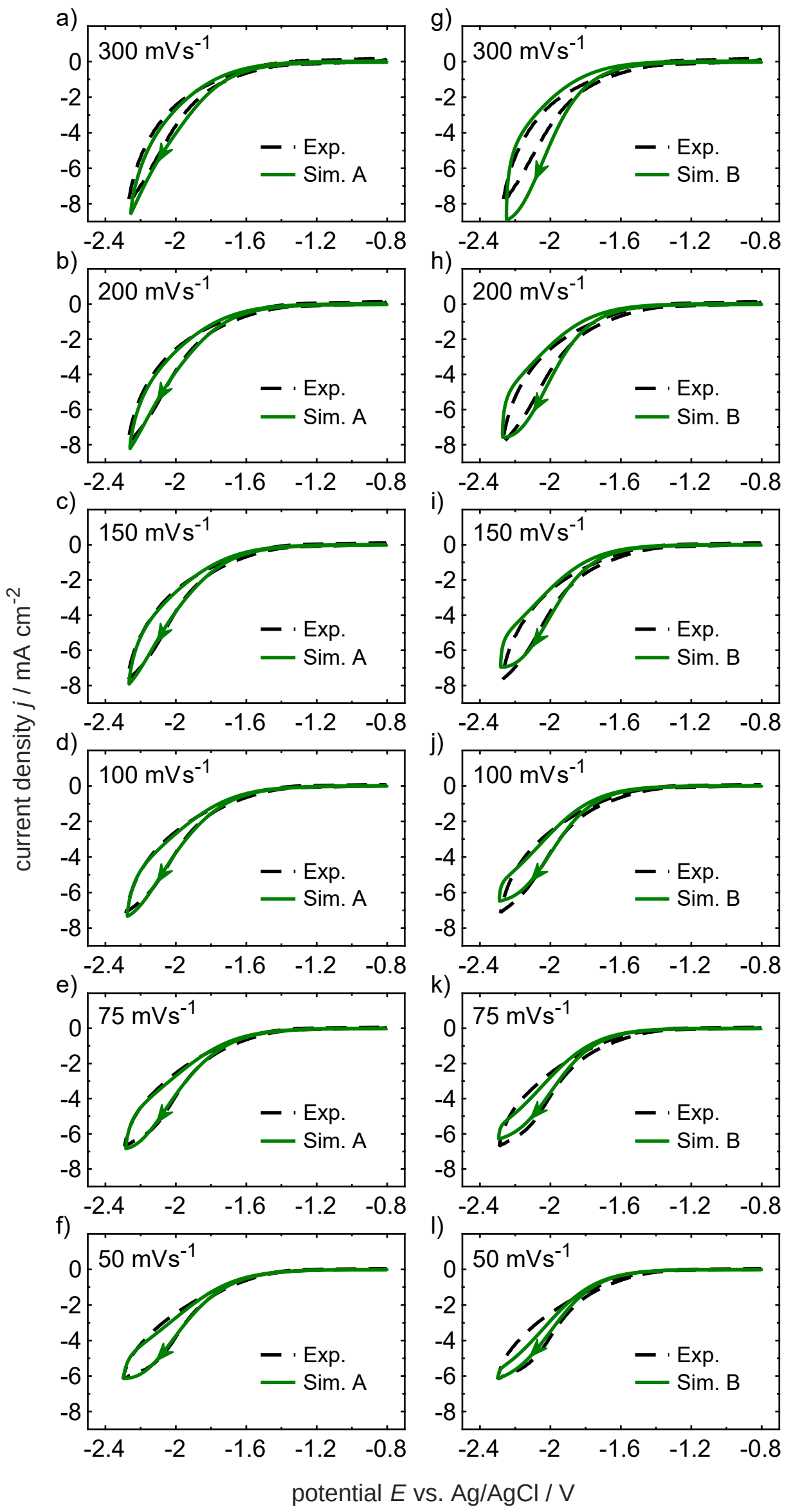

Figure S19. Comparison of simulated (solid line) and experimental (dashed line) CVs for a planar CuZn25 electrode in CO_2-saturated 0.1 mol L^{-1} TBABF$_4$ acetonitrile electrolyte. CVs are presented for scan rates from 300 mV s^{-1} to 50 mV s^{-1} for Case A (a to f) and Case B (g to l). All CVs are *iR*-corrected and represent the third cycle of each measurement.

8.1. Model Validation

Table S7. Normalized root mean squared error values (f_{obj}) for each scan rate, Case (A and B), and alloy composition, calculated based on the comparison of simulated and experimental current densities.

		f_{obj} / –						
		300 mV s^{-1}	200 mV s^{-1}	150 mV s^{-1}	100 mV s^{-1}	75 mV s^{-1}	50 mV s^{-1}	Mean
Case A	Cu	0.0693	0.0235	0.0270	0.0272	0.0290	0.0366	0.0354
	CuZn10	0.0648	0.0487	0.0459	0.0454	0.0684	0.0828	0.0593
	CuZn20	0.0182	0.0106	0.0228	0.0264	0.0278	0.0174	0.0205
	CuZn25	0.0211	0.0127	0.0108	0.0100	0.0113	0.0135	0.0133
Case B	Cu	0.0726	0.0400	0.0305	0.0246	0.0202	0.0220	0.0350
	CuZn10	0.0874	0.0519	0.0511	0.0721	0.0984	0.1124	0.0789
	CuZn20	0.0617	0.0614	0.0740	0.0771	0.0764	0.0686	0.0699
	CuZn25	0.0532	0.0324	0.0319	0.0278	0.0295	0.0377	0.0354

8.2. Kinetic Parameter

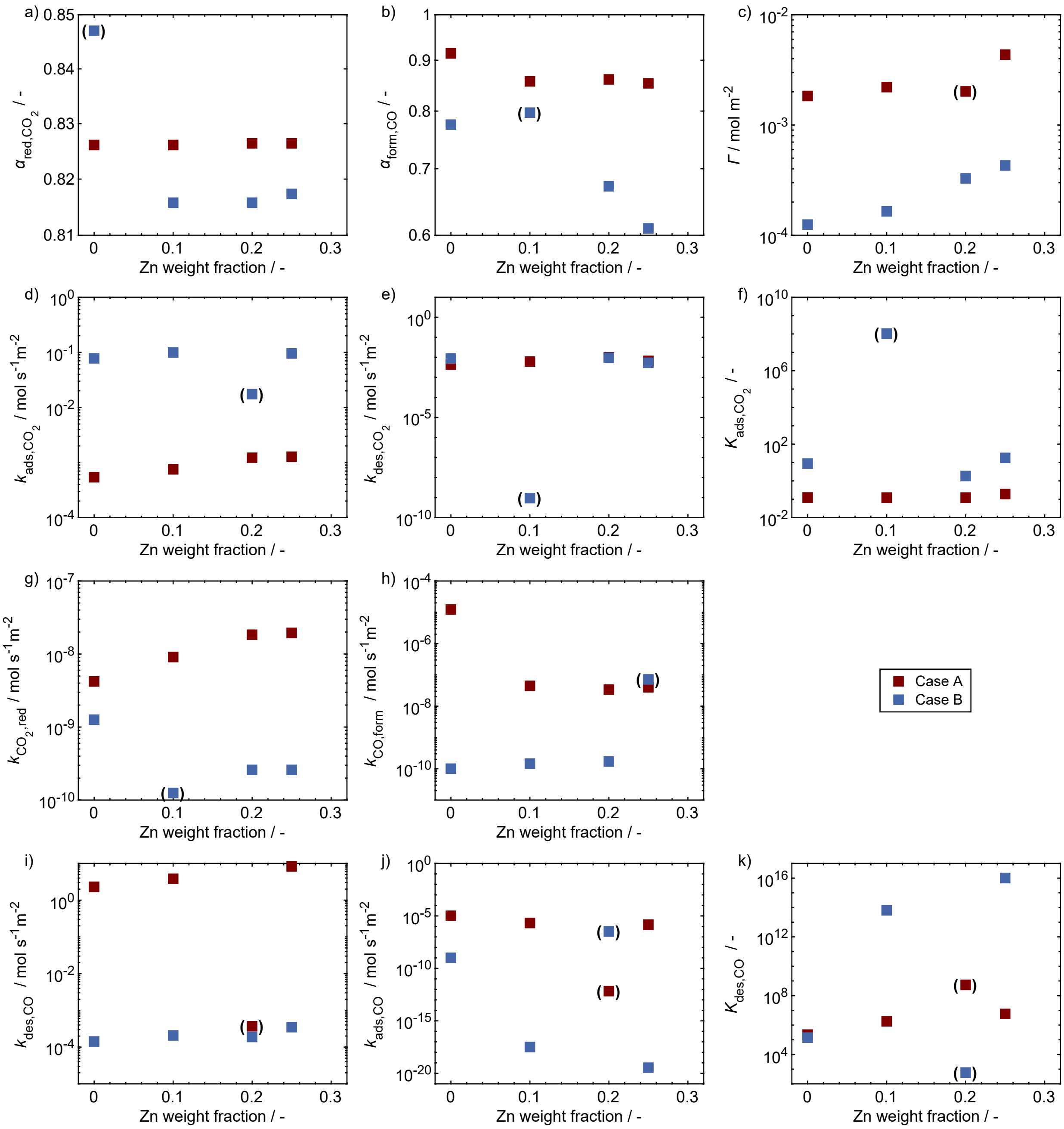

Figure S20. Complete sets of identified kinetic parameter values for Case A (red) and Case B (blue) as a function of Zn mass fraction. K_{ads,CO_2} and $K_{des,CO}$ represent the equilibrium constant of CO_2 adsorption and CO desorption, respectively. Outliers are marked by brackets ( ).

9. Steady-state Simulation on Roughened CuZn25

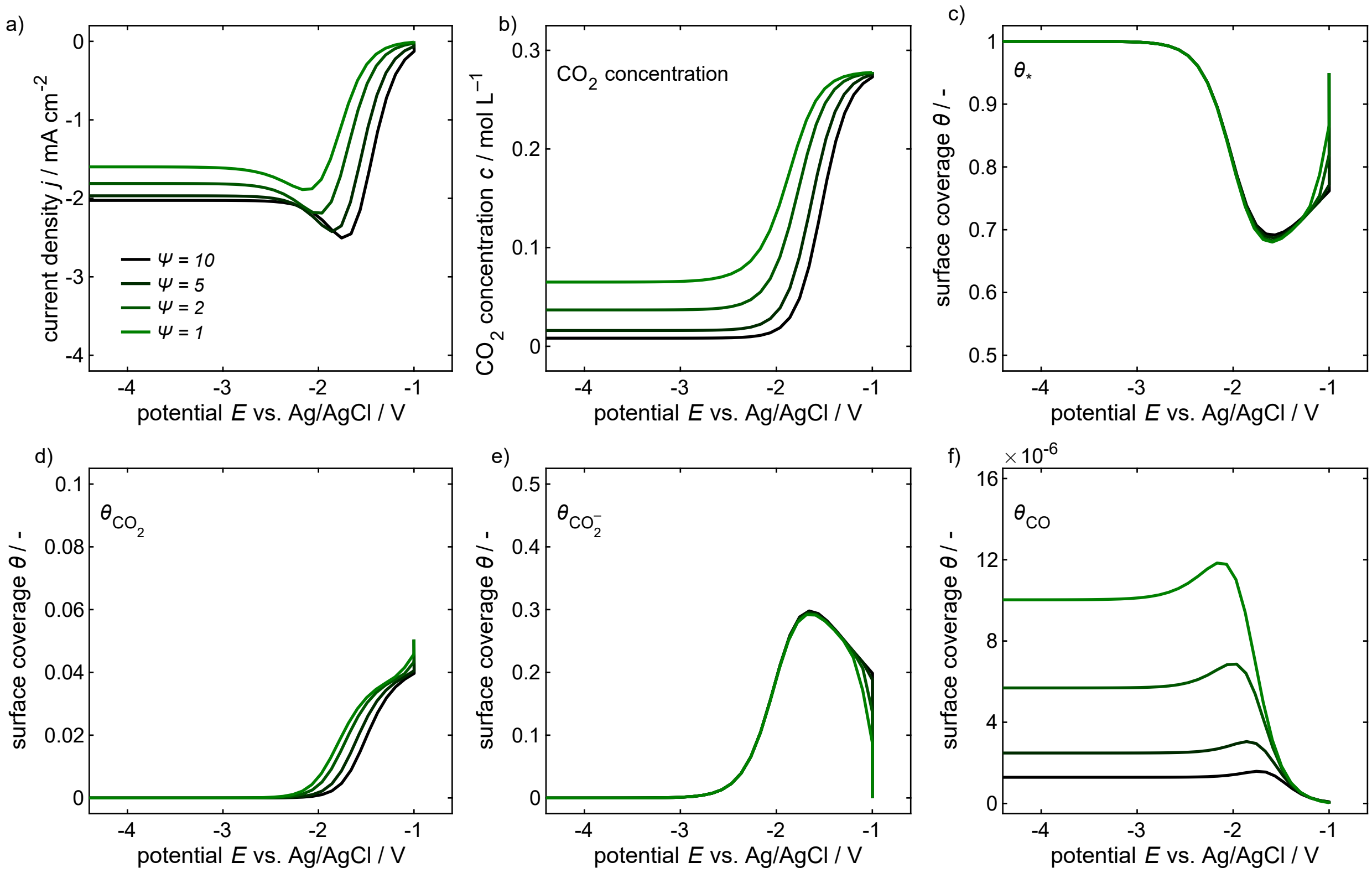

Figure S21. Simulated steady-state polarization curve for CuZn25 using parameters from Case A, demonstrating the effect of surface roughness via the geometric factor Ψ (ratio of geometric to electrochemically active surface area), varied from 1 to 10. $\Psi = 1$ corresponds to the planar CuZn25 electrode in the main manuscript. Panels show: a) current densities j, b) interfacial CO_2 concentration c_{CO_2}, c) free surface coverage θ_*, d) CO_2 coverage θ_{CO_2}, e) $^*CO_2^-$ coverage $\theta_{CO_2^-}$, and f) *CO coverage on a magnified scale (10^{-6}). Potentials stepped in 0.1 V increments, each held for 600 s to reach steady-state.

10. Parameter Sensitivity Analysis

To investigate the influence of kinetic parameters (α_{red,CO_2}, $\alpha_{form,CO}$, Γ, k_{ads,CO_2}, k_{des,CO_2}, k_{red,CO_2}, $k_{form,CO}$, $k_{des,CO}$, and k_{adsCO}) on the simulated cyclic voltammograms for the CuZn electrocatalysts, a sensitivity analysis was conducted based on the parameter sets for pure Cu from each Case. Each kinetic parameter in our model was independently varied by factors of 0.01, 0.1, 10 and 100 while the other parameters were held constant. For charge transfer coefficients α_{red,CO_2} and $\alpha_{form,CO}$, which appear within exponential functions, the variation was more nuanced, with factors of 0.9, 0.99, 1.01 and 1.1 used to assess their sensitivity.

10.1. Case A

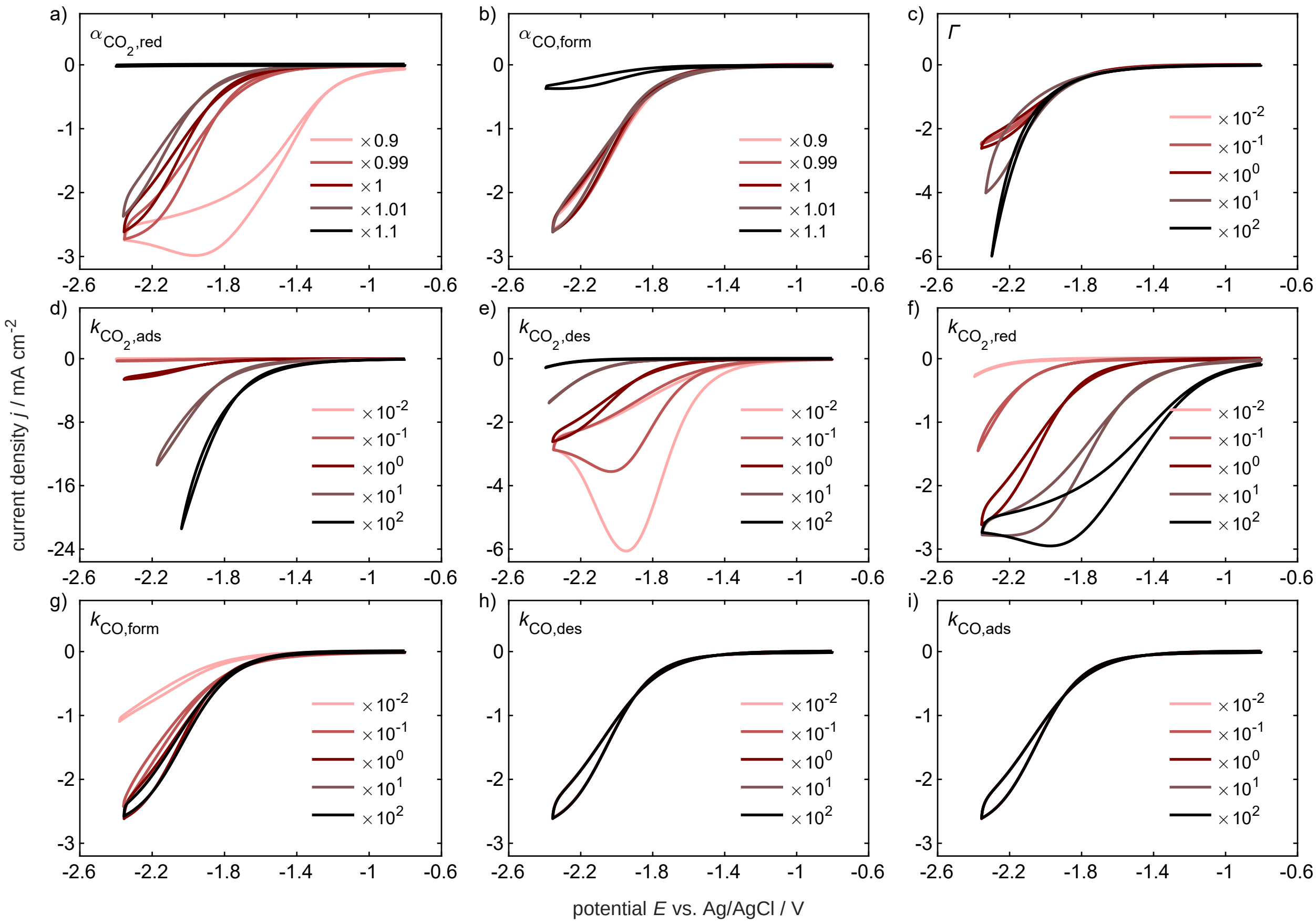

Figure S22. Sensitivity analysis of simulated CVs to variations in kinetic parameters for pure Cu based on Case A. Each subfigure shows the simulated CVs for different variations of a specific kinetic parameter, while keeping all other parameters constant. a) Charge transfer coefficient for CO_2 reduction (α_{red,CO_2}). b) Charge transfer coefficient for CO formation ($\alpha_{form,CO}$). c) Concentration of adsorption sites (Γ). d) Rate constant for CO_2 adsorption (k_{ads,CO_2}). e) Rate constant for CO_2 desorption (k_{des,CO_2}). f) Rate constant for CO_2 reduction (k_{red,CO_2}). g) Rate constant for CO formation ($k_{form,CO}$). h) Rate constant for CO desorption ($k_{des,CO}$). i) Rate constant for CO adsorption ($k_{ads,CO}$).

10.2. Case B

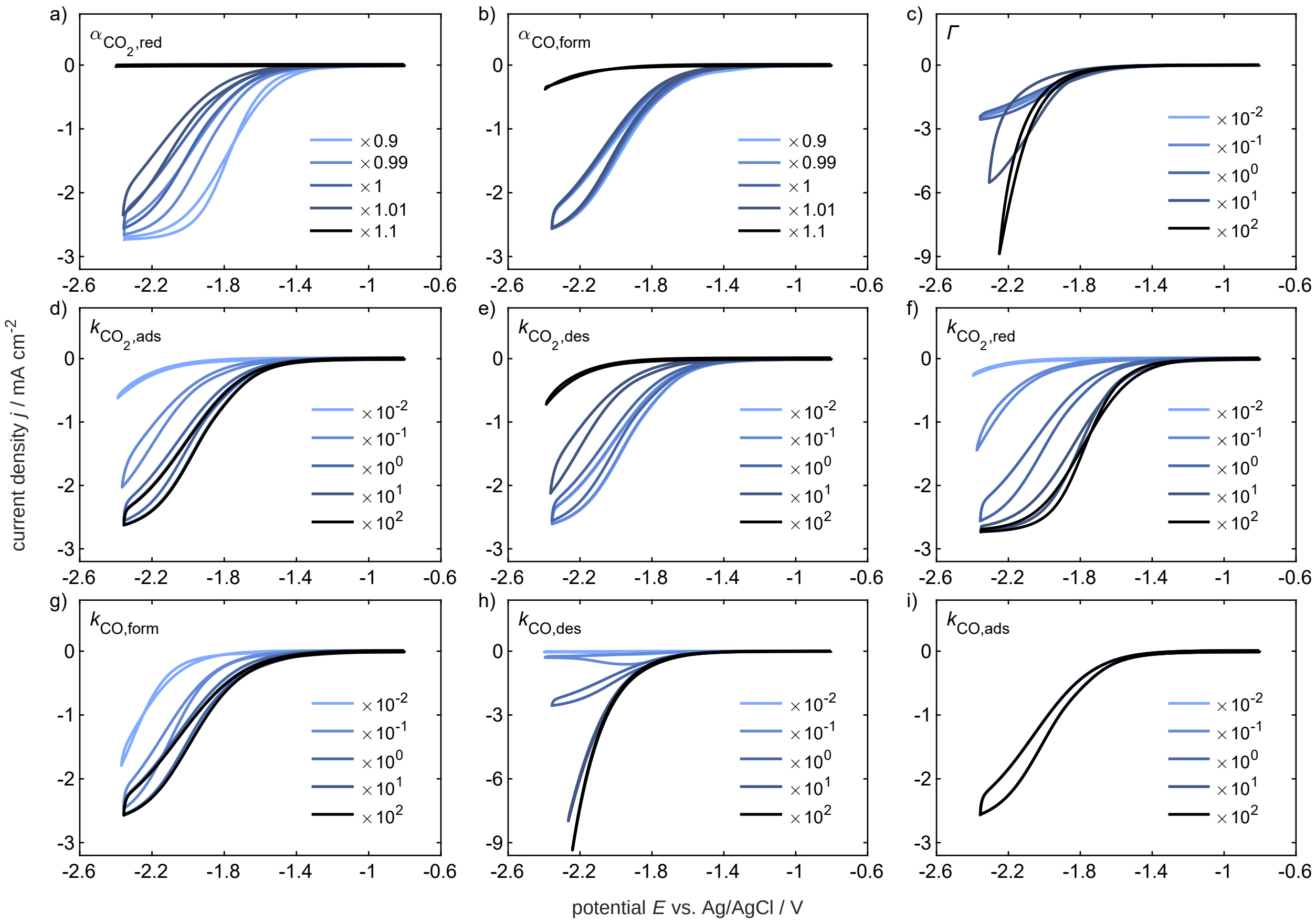

Figure S23. Sensitivity analysis of simulated CVs to variations in kinetic parameters for pure Cu based on Case B. Each subfigure shows the simulated CVs for different variations of a specific kinetic parameter, while keeping all other parameters constant. a) Charge transfer coefficient for CO_2 reduction (α_{red,CO_2}). b) Charge transfer coefficient for CO formation ($\alpha_{form,CO}$). c) Concentration of adsorption sites (Γ). d) Rate constant for CO_2 adsorption (k_{ads,CO_2}). e) Rate constant for CO_2 desorption (k_{des,CO_2}). f) Rate constant for CO_2 reduction (k_{red,CO_2}). g) Rate constant for CO formation ($k_{form,CO}$). h) Rate constant for CO desorption ($k_{des,CO}$). i) Rate constant for CO adsorption ($k_{ads,CO}$).

11. Simulation of Case A with Trend-consistent CuZn20 Parameters

In this section, a parameter set is constructed for CuZn20 in which the originally identified kinetic outliers from Case A are adjusted to follow the composition-dependent trends of Cu, CuZn10, and CuZn25. The following parameters were replaced from the originally identified parameter set of CuZn20: $\Gamma = 3.665 \times 10^{-3}$ mol m^{-2}, $k_{\mathrm{des,CO}} = 6.828$ mol s^{-1} m^{-2}, and $k_{\mathrm{ads,CO}} = 1.873 \times 10^{-6}$ mol s^{-1} m^{-2}

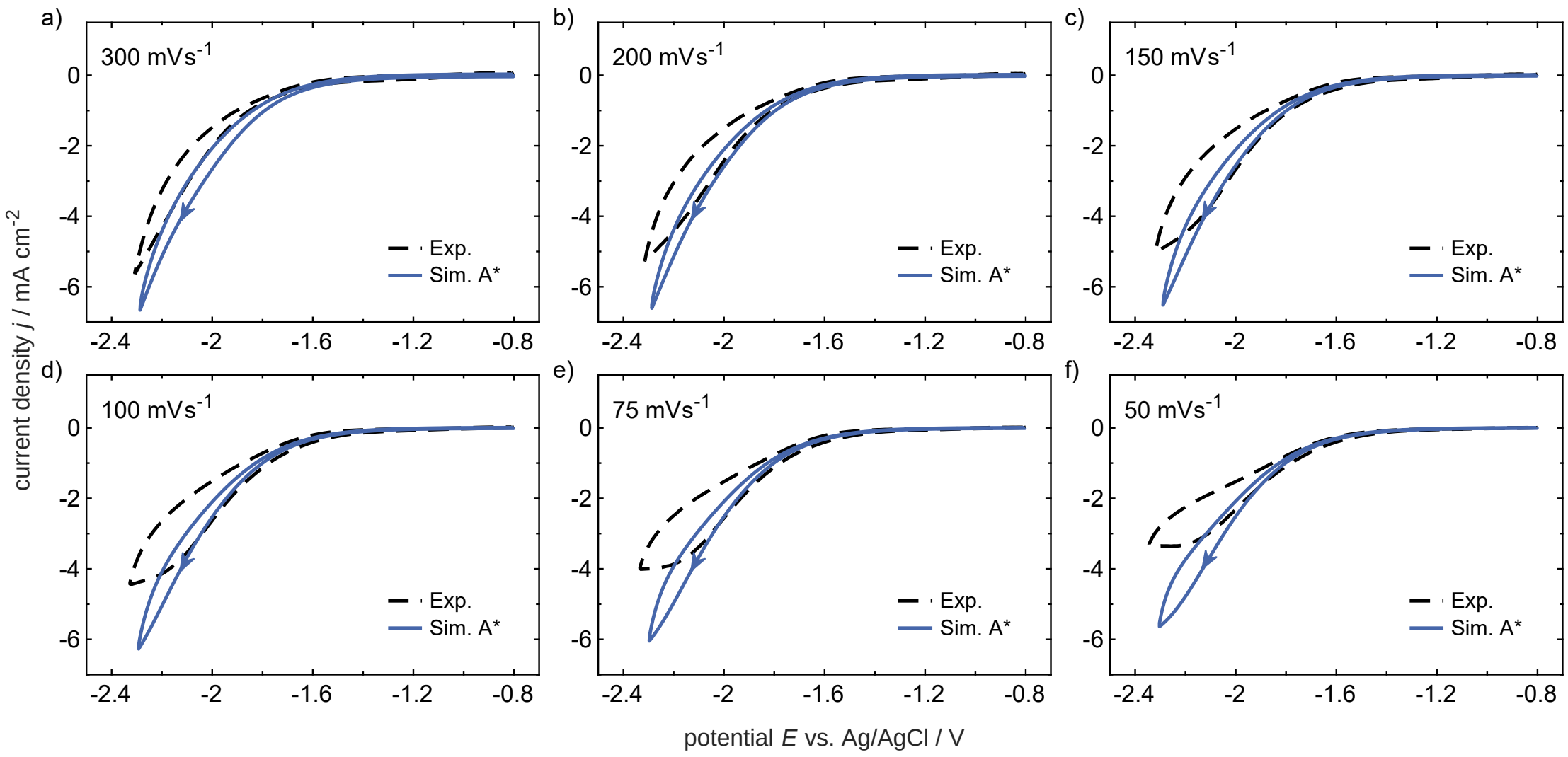

Figure S24. Comparison of simulated (solid line), using the adjusted parameter set (see text above) and experimental (dashed line) CVs for a planar CuZn20 electrode in CO_2-saturated 0.1 mol L^{-1} TBABF$_4$ acetonitrile electrolyte. CVs are presented for scan rates from 300 mV s^{-1} to 50 mV s^{-1} for Case A (a to f). All CVs are *iR*-corrected and represent the third cycle of each measurement.

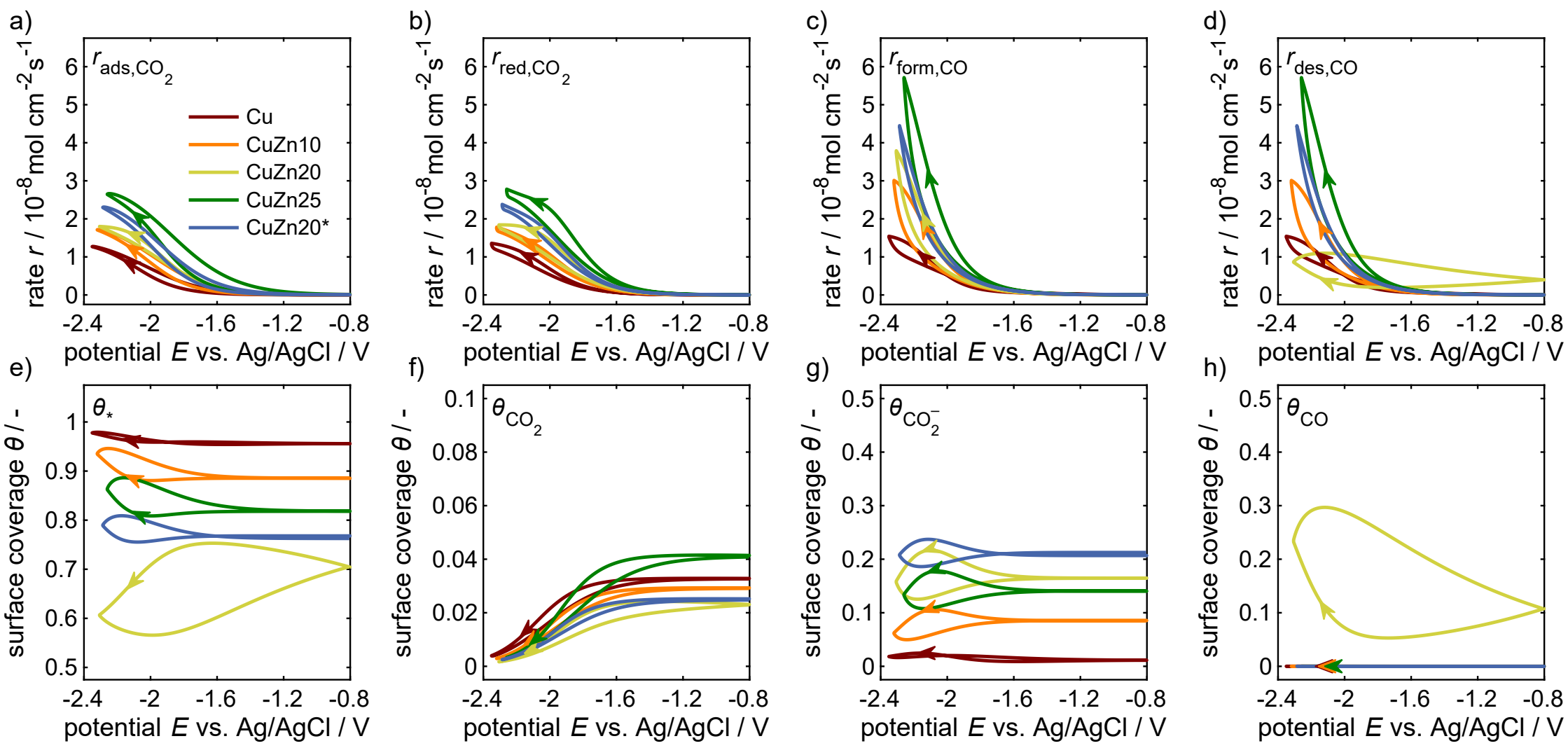

Figure S25. Simulated reaction rates and surface coverages during cyclic voltammetry at 200 mV s^{-1} for Cu and α-CuZn electrocatalysts based on Case A with the adjusted parameter set (see text above) labeled as CuZn20* in blue: a) CO_2 adsorption rate, b) CO_2 reduction rate, c) CO formation rate, d) CO desorption rate, e) free surface site coverage (*), f) adsorbed CO_2 coverage (*CO_2), g) adsorbed CO_2 radical anion coverage (*CO_2^-), and h) and adsorbed CO coverage (*CO).

12. Impact of Electrolyte Decomposition on Kinetic Modeling

12.1. Background Correction for Electrolyte Decomposition

To assess whether electrolyte decomposition also overlaps with the previous measurements under CO_2 atmosphere, the CVs recorded under pure CO atmosphere (denoted as 100 vol.% CO in the main article) were used as a background and subtracted from the corresponding CO_2 CVs (0 vol.% CO) for Cu and CuZn25.
Subsequently, the kinetic parameters of Case A were re-fitted to these background-corrected data. The resulting analysis shows the same mechanistic interpretation as the original fit in the main article, indicating that our conclusions are robust with respect to the contribution of a potential electrolyte decomposition.

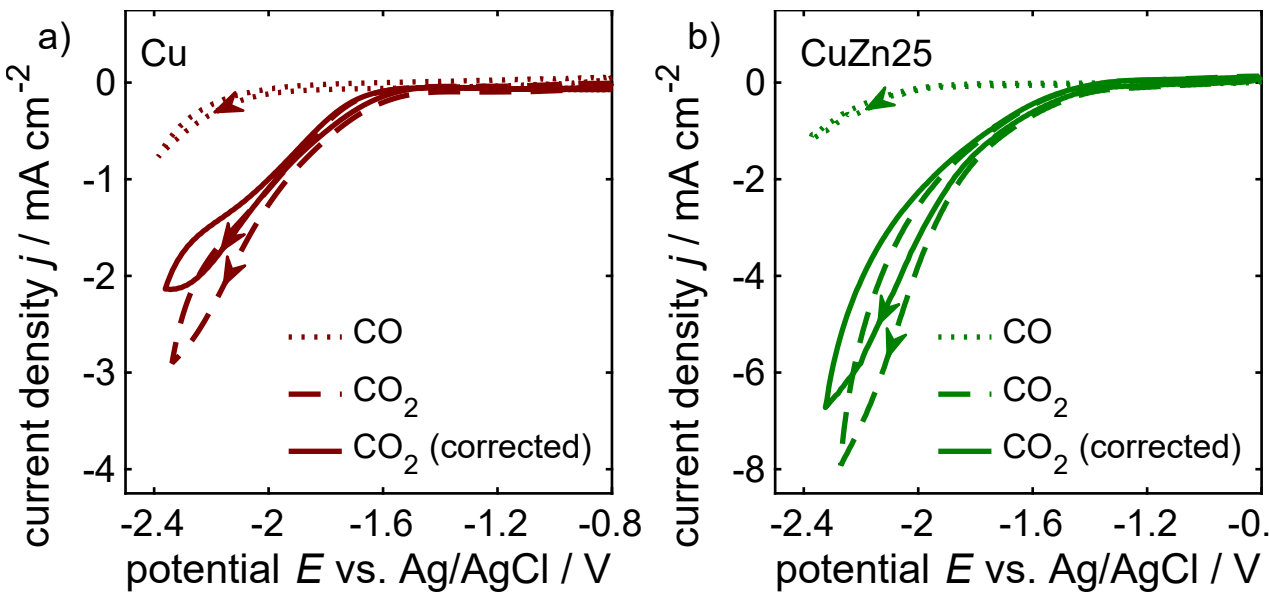

Figure S26. Background-corrected cyclic voltammograms (CVs) for Cu and CuZn25 in 0.1 mol L^{-1} $TBABF_4$ acetonitrile electrolyte at room temperature and a scan rate of 200 mV s^{-1}. For each catalyst, the CV under 100 vol.% CO was subtracted from the corresponding CV under 100 vol.% CO_2.

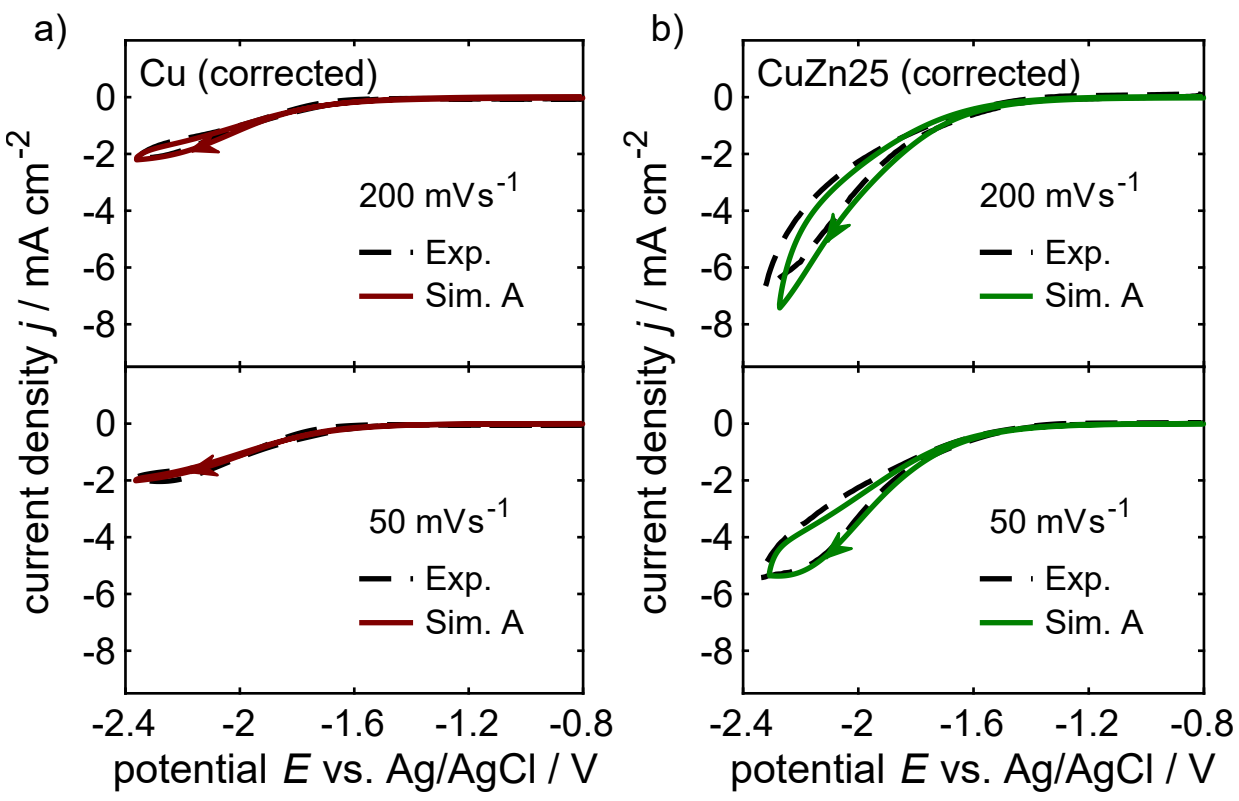

Figure S27. Background-corrected experimental (dashed, black) and simulated (solid, colored) CVs for CO_2 reduction on Cu and CuZn25 at 200 mV s^{-1} and 50 mV s^{-1} in CO_2-saturated 0.1 mol L^{-1} $TBABF_4$ in acetonitrile at room temperature. Simulations use the re-fitted Case A parameters.

Table S8. Re-fitted kinetic parameters of Case A for Cu and CuZn25 obtained from background-corrected CVs.

Parameter	Unit	Cu	CuZn25
α_{red,CO_2}	–	0.8260	0.8264
$\alpha_{form,CO}$	–	0.9129	0.8540
Γ	mol m^{-2}	4.2865×10^{-4}	3.6825×10^{-3}
k_{ads,CO_2}	mol s^{-1} m^{-2}	5.2948×10^{-4}	1.1448×10^{-3}
k_{des,CO_2}	mol s^{-1} m^{-2}	3.0684×10^{-3}	6.5983×10^{-3}
k_{red,CO_2}	mol s^{-1} m^{-2}	4.2080×10^{-9}	2.3653×10^{-8}
$k_{form,CO}$	mol s^{-1} m^{-2}	2.2440×10^{-6}	3.7040×10^{-8}
$k_{des,CO}$	mol s^{-1} m^{-2}	6.8285×10^{-4}	7.0291×10^{-3}
$k_{ads,CO}$	mol s^{-1} m^{-2}	2.1259×10^{-19}	4.6703×10^{-6}

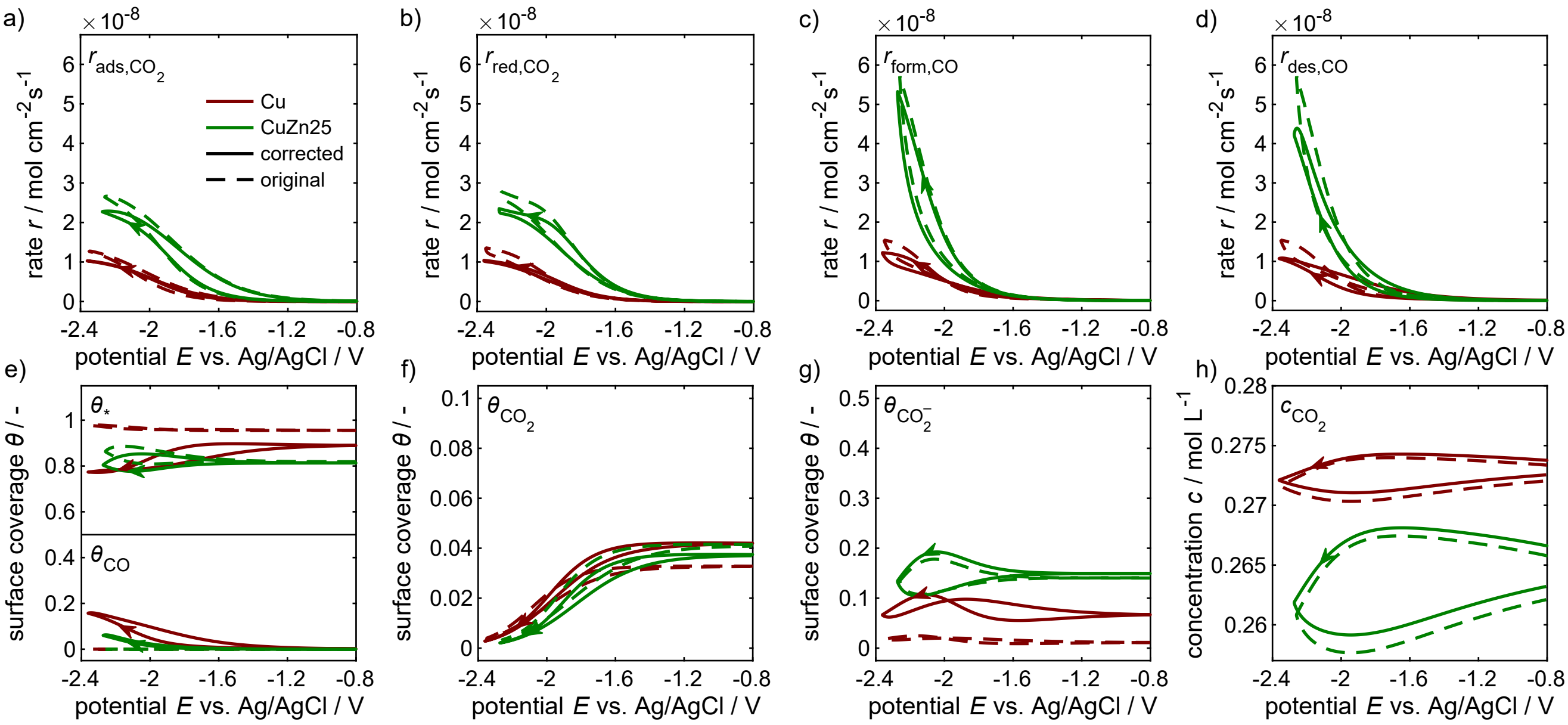

Figure S28. Simulated reaction rates and surface coverages during cyclic voltammetry at 200 mV s^{-1} for Cu and CuZn25 electrocatalysts, comparing simulations based on the re-fitted kinetic parameters of Case A (background-corrected) with simulations using the original parameter set from the main article: a) CO_2 adsorption rate, b) CO_2 reduction rate, c) CO formation rate, d) CO desorption rate, e) free surface site coverage (*) (upper part) and adsorbed CO coverage (*CO) (lower part), f) adsorbed CO_2 coverage (*CO_2), g) adsorbed CO_2 radical anion coverage (*CO_2^{-}), and h) concentration of dissolved CO_2 at the electrode-electrolyte interface.

12.2. Cyclic Voltammograms under Ar Atmosphere

To characterize the electrolyte decomposition behavior independently of CO_2 or CO reduction, additional CVs were recorded under an Ar atmosphere. These measurements reveal that, while the onset potential for electrolyte decomposition is similar to that observed under pure CO (100 vol.% CO), the CVs in Ar-saturated electrolyte exhibit additional peaks and features that do not appear under CO_2 or CO. The processes responsible for these additional peaks may be dominated by impurities or surface changes, which are likely negligible when sufficient CO_2 or CO is supplied. As a result, Ar-based CVs are unsuitable for background correction of the CO_2 measurements. A reliable and transferable kinetic description of solvent breakdown would require a separate dedicated study, which is beyond the scope of the present work.

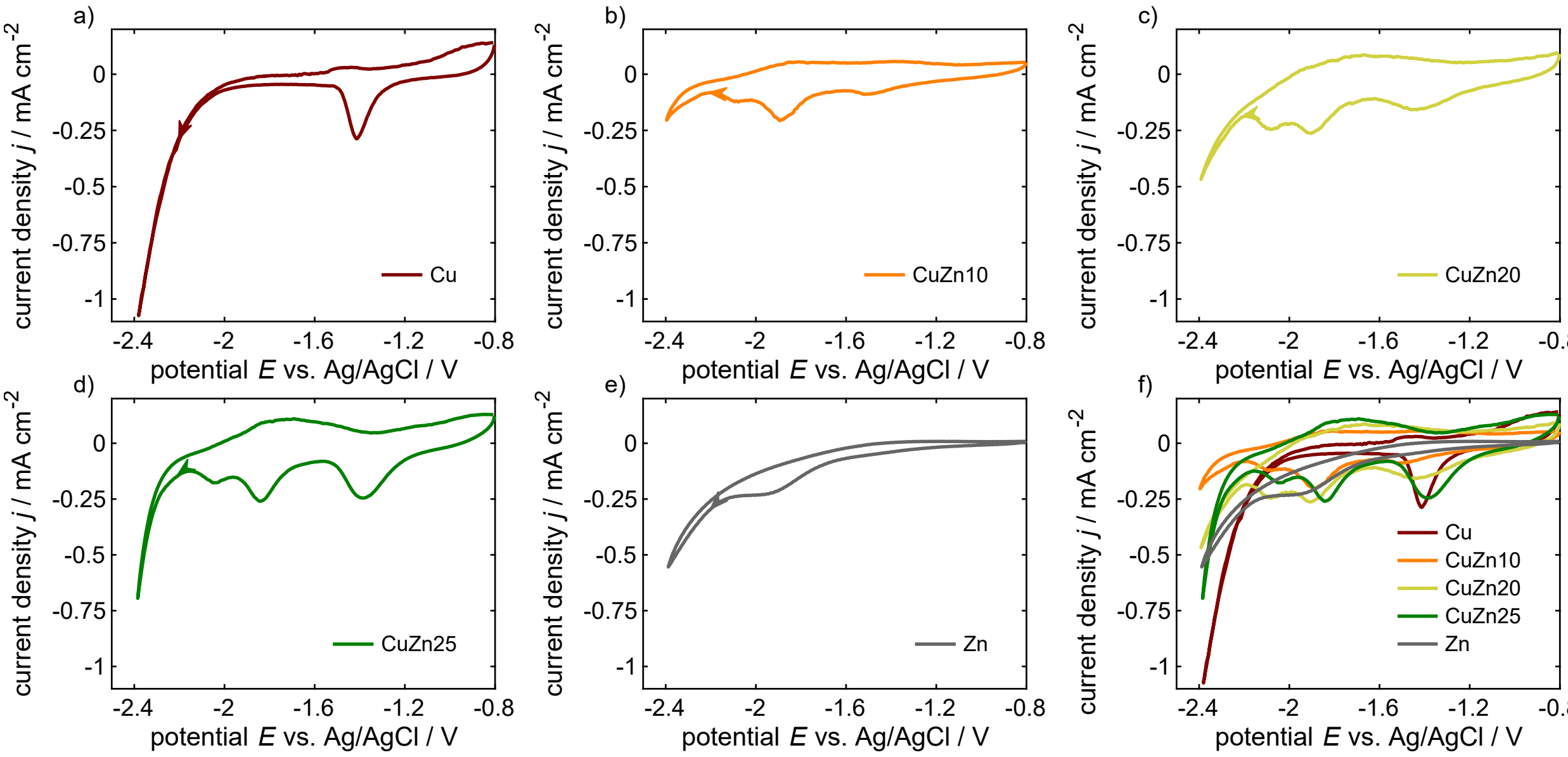

Figure S29. Experimental cyclic voltammograms (CVs) recorded under Ar atmosphere for a) Cu, b) CuZn10, c) CuZn20, d) CuZn25, and e) Zn electrodes in 0.1 mol L^{-1} $TBABF_4$ acetonitrile electrolyte at room temperature and a scan rate of 200 mV s^{-1}. Panel f) overlays all CVs to highlight differences in current response and additional decomposition-related features observed under Ar.

References

[1] E. A. Owen, G. D. Preston, *Proc. Phys. Soc.* **1923**, *36*, 49.
[2] A. M. Dudzinski, E. Diesen, K. Reuter, V. J. Bukas, *The Journal of Physical Chemistry C* **2024**, *128*, 20886.
[3] J. A. Gauthier, C. F. Dickens, S. Ringe, K. Chan, *ChemPhysChem* **2019**, *20*, 3074.
